# Projections of disability-adjusted life years for major diseases due to a change in vegetable intake in 2017–2040 in Japan

**DOI:** 10.1186/s12889-021-10772-2

**Published:** 2021-04-21

**Authors:** Shiori Tanaka, Daisuke Yoneoka, Aya Ishizuka, Peter Ueda, Keiji Nakamura, Hisayuki Uneyama, Naoki Hayashi, Kenji Shibuya, Shuhei Nomura

**Affiliations:** 1 Department of Global Health Policy, Graduate School of Medicine, The University of Tokyo, 7-3-1, Hongo, Bunkyo-ku, Tokyo, 113-0033 Japan; 2 Epidemiology and Prevention Group, Center for Public Health Sciences, National Cancer Center, 5-1-1 Tsukiji, Chuo-ku, Tokyo, 104-0045 Japan; 3 Graduate School of Public Health, St. Luke’s International University, Tokyo, Japan; 4 Department of Health Policy and Management, School of Medicine, Keio University, Tokyo, Japan; 5 Clinical Epidemiology Division, Department of Medicine, Solna, Karolinska Institute, Stockholm, Sweden; 6 Graduate School of Environmental and Information Studies, Tokyo City University, Yokohama, Japan; 7 Ajinomoto Co., Inc., Tokyo, Japan; 8 Department of Applied Biological Chemistry, Graduate School of Agriculture and Life Sciences, The University of Tokyo, Tokyo, Japan; 9 Institute for Population Health, King’s College London, London, UK

**Keywords:** Disability-adjusted life years, Vegetable consumption, Japan, Future projection, Cardiovascular diseases, Cancer, Diabetes and kidney diseases

## Abstract

**Background:**

Low vegetable intake is one of the key dietary risk factors known to be associated with a range of health problems, including cardiovascular diseases (CVDs), cancer, and diabetes and kidney diseases (DKDs). Using data from Japan’s National Health and Nutrition Surveys and the Global Burden of Diseases study in 2017, this study aimed to forecast the impact of change in vegetable intake on disability-adjusted life years (DALYs) between 2017 and 2040 for three diseases.

**Methods:**

We generated a three-component model of cause-specific DALYs, including changes in major behavioural and metabolic risk predictors, the socio-demographic index and an autoregressive integrated moving average model to project future DALY rates for 2017–2040 using the data between 1990 and 2016. Data on Vegetable consumption and risk predictors, and DALY rate were obtained from Japan’s National Health and Nutrition Surveys and the Global Burden of Diseases Study in 2017. We also modelled three scenarios of better, moderate and worse cases to evaluate the impact of change in vegetable consumption on the DALY rates for three diseases (CVDs, cancer, and DKDs).

**Results:**

Projected mean vegetable intake in the total population showed a decreasing trend through 2040 to 237.7 g/day. A significant difference between the reference scenario and the better case scenario was observed with un-overlapped 95% prediction intervals of DALY rates in females aged 20–49 years (− 8.0%) for CVDs, the total population for cancer (− 5.6%), and in males (− 8.2%) and females (− 13.7%) for DKDs.

**Conclusions:**

Our analysis indicates that increased vegetable consumption would have a significant reduction in the burdens of CVDs, cancer and DKDs in Japan. By estimating the disease burden attributable to low vegetable intake under different scenarios of future vegetable consumption, our study can inform the design of targeted interventions for public health challenges.

**Supplementary Information:**

The online version contains supplementary material available at 10.1186/s12889-021-10772-2.

## Introduction

Epidemiological research has shown that increased vegetable is associated with a reduced risk of several NCDs, including CVDs, high blood pressure, diabetes, cancer and metabolic syndromes [1–5]. Furthermore, it has been reported that an optimal amount of vegetable consumption reduces the disease burden of CVDs, cancer and diabetes [6]. In the 2017 Global Burden of Disease (GBD) analysis in Japan, assessing the impact of 67 risk factors, including behavioural, metabolic, environmental and occupational factors, on disease burden measured as disability-adjusted life years (DALYs), low vegetable intake was the fifth most significant dietary risk factor affecting DALYs, following diets high in sodium, low in whole grains, fruits, nuts and seeds [7, 8].

The recommended amount of vegetable consumption varies per country [9]. In Japan, the government has set a consumption target for an average of 350 g of vegetables per capita by 2023 in their ten-year national health promotion plan “The second term of National Health Promotion Movement in the twenty-first century” (Health Japan 21 (the second term)) [10]. According to the National Health and Nutrition Survey (NHNS), however, the average vegetable consumption per capita for Japanese adults has steadily been below 300 g/day since 1947 (with an exception for 2006) [11, 12]. Moreover, the trend points towards a decrease in vegetable consumption in recent years [13], and the consumption target is thus unlikely to be attained.

This study aims to predict the future trend in vegetable intake and to estimate the disease burden of CVDs, cancer, DKDs under several scenarios of vegetable intake in Japan. By doing so, we aim to provide an empirical basis for future interventions and policies to improve the health of the people in Japan and other countries with low vegetable consumption.

## Methods

### Overall forecasting model structure

We followed the methodological approach of Nomura and Yoneoka et al. [14]. GBD data from 1990 to 2016 were used to predict the future values from 2017 to 2040 for DALYs attributable to three disease groups that have been identified to be associated with low vegetable intake. DALYs is a comprehensive measure of disease burden, comprised of years lived with disability and years of life lost due to premature death. The three disease groups were neoplasms, CVDs and DKDs from level 2 in the GBD hierarchical causal structure. The GBD hierarchy causal structure ranges from level 1 of the three basic groups (communicable; maternal and neonatal conditions, and nutritional deficiencies; non-communicable diseases; and injuries) to level 4 of the most detailed 359 diseases groups.

Following the GBD’s prediction methodology [15], we developed a three-component model of cause-specific DALYs for the three disease groups. This model included a component explained by changes in major behavioural and metabolic risk predictors including vegetable consumption, which is the main predictor of interest in this project, socio-demographic index (SDI), and an autoregressive integrated moving average (ARIMA) model that captures the unexplained components over time. Separate projections models were developed by sex and three age groups (20–49, 50–69, and ≥ 70 years,and all ages). The 0–19 years age group was not included in the model because of a lack of data on risk predictors including smoking and alcohol intake. Further detailed information on data and model formula is described below.

### DALYs and SDI data, 1990–2016

We used the estimated DALY rates per 100,000 population for CVDs, all cancers, and DKDs, as well as SDI in Japan for the years 1990–2016 reported in GBD 2017 [16]. SDI is a composite indicator of development status strongly correlated with health outcomes, wherein a 0 to 1 index value was determined for each of the original three covariate inputs, including total fertility rate under the age of 25, mean education for those aged 15 and older, and lag distributed income [16]. An index score of 0 represents the minimum level of each covariate input, while an index score of 1 represents the maximum level of each covariate.

### Behavioural and metabolic risk predictor data, 1990–2016

We used consecutive nationwide data from NHNS to characterise sex- and age-specific average daily vegetable intake and prevalence of current smoker, current alcohol drinker, and obesity (defined according to the Japanese definition of a body mass index (BMI) of 25 kg/m^2^ or over) following the design of a previous study [14]. The individual data from NHNS were categorised according to the GBD’s category and combined with DALYs rate by age, sex and survey year. The NHNS is a nationally representative household survey, which is conducted annually by the Japanese Ministry of Health, Labour and Welfare in order to capture the distribution of dietary habits, nutrition intake and lifestyle at a population level [17]. Residents aged ≥1 years-old in all households from Census enumeration areas are selected using a stratified single-stage cluster sample design. The NHNS consists of three parts: 1) physical examination including a blood test performed by a medical team at community centres; 2) an in-person survey of a weighted single-day dietary record of households investigated by registered dieticians who visit and check the participant’s survey compliance; 3) a self-reported lifestyle questionnaire such as smoking status and alcohol consumption. Only those aged 20 years or older were subjects to the lifestyle questionnaire. Details of survey design and procedures of NHNS are available elsewhere [11, 17]. The dietary intake survey was conducted on a single designated day by household representatives, usually by those who are responsible for food preparation. Trained interviewers (mainly registered dietitians) instructed household representatives on the measurement of food and beverages consumed by the household members and verified the survey compliance. The proportion of shared dishes, food waste, and foods that were eaten out were also recorded. The nutrient intake and food consumption were estimated using the dietary record and the corresponding food composition list of the Japanese Standard Tables of Food Composition [18] We also obtained individual-level data on the intake of green and yellow vegetables, other vegetables, vegetable juices and salted vegetables. The data on food consumption from 1995 was used because individual food consumption values before 1994 were not available.

In this study, vegetable intake was defined as the intake of green and yellow vegetables and other vegetables by referring to GBD 2017 definition as follows: average daily consumption of vegetables (fresh, frozen, cooked, canned or dried vegetables excluding legumes and salted or pickled vegetables, juices, nuts and seeds, and starched vegetables such as potatoes or corn) [7].

This study was performed in accordance with the Declaration of Helsinki. The Research Ethics Committee of the Graduate School of Medicine, The University of Tokyo approved this study (authorization number 11964) and waived the need for informed consent as this study was a secondary analysis of anonymised data that is collected routinely by the MHLW. Data of GBD 2017 are also secondary, aggregated estimates by country, sex, and age groups.

### ARIMA model for forecast

The ARIMA model, one of the most classic methods of time series analysis, was employed to forecast future DALY rates adjusting for several risk predictors. It is a moving average (MA) model combined with an autoregression (AR) model to fit the temporal dependence structure of a time series using the shift and lag of historical information.

The Box–Jenkins methodology was adopted to fit the ARIMA (p, d, q) model with orders p, d, q:

1 $$ \left(1-\sum \limits_{i=1}^p{\alpha}_i{L}^i\right){\left(1-L\right)}^d{y}_t=\left(1+\sum \limits_{i=1}^q{\beta}_i{L}^d\right){\varepsilon}_t, $$

where *y*_*t*_ is the outcome of interest, *ε*_*t*_ is an (white noise) error term with an intensity of *σ*^2^ at time *t*, *L* is time lag operator defined as *L*^*k*^*y*_*t*_ = *y*_*t* − *k*_, and *α*_*i*_ and *β*_*i*_ are the coefficient parameters [19]. Before constructing the model, the stationary state of observed data in the series must be identified using Dickey-Fuller test [19]. All variables were log-transformed. If non-stationary is assumed to be plausible, the data was transformed into a stationary time series by taking a suitable difference with order d. The autocorrelation function and the partial autocorrelation function was used to identify the stationary status and the search range for orders of the model. The model estimation was carried out after an initial model has been identified; generally, model parameters are estimated by using maximum likelihood methods. Akaike’s Information Criterion (AIC) was calculated to select optimal models with the orders.

We used a two-step approach to forecast the DALY rates: the first step was to independently forecast the values of each predictor from 1995 until 2040 using Eq. (1), and then the second step was to forecast the log-scaled DALY rates by using the Eq. (2) after inserting the predicted values of the above predictors into the model as illustrated below:

2 $$ \left(1-\sum \limits_{i=1}^p{\alpha}_i{L}^i\right){\left(1-L\right)}^d{y}_t=\sum \limits_{j=1}^4{\upgamma}_j{L}^d{x}_{tj}+\left(1+\sum \limits_{i=1}^q{\beta}_i{L}^d\right){\varepsilon}_{t,} $$

where *y*_*t*_ was the DALY rate at time *t*, *x*_*tj*_ was the value of *j* th predictor at time *t* and *γ*_*j*_ was a coefficient parameter for *j* th predictor. All analyses were conducted by STATA version 16 using the ARIMA procedure. The parameters in Eq. (2) were separately estimated by age and sex groups.

### Future scenario analysis for vegetable consumption

We assumed three scenarios of better, moderate and worse cases to evaluate the impact of change in vegetable consumption on the DALY rates for three diseases (CVDs, cancer, and DKDs) for 2017–2040 using the smoothed data between 1995 and 2016. A reference forecast was set as the current trend was maintained, namely the projected vegetable consumption during 1995–2040 derived from the ARIMA model in Eq. (1). The better case scenario considered achieving the target daily vegetable consumption (350 g/ day) in 2023 as per Health Japan 21 (the second term), which the guideline provides as the recommendation and goals of lifestyle to improve population health defined by the Japanese government. This scenario assumed a constant monotonic increasing function from 2017 to 2023 and a constant level of 350 g/day after 2023. The moderate case scenario assumed that the target vegetable consumption would be achieved in 2040 rather than in 2023 with the monotonic increase function. As such, vegetable consumption as of 2040 was the same values in the better and moderate case scenarios. The worse case scenario assumed a constant monotonic decrease in vegetable consumption from 2017 to 2040 by the level of 2004 when the lowest consumption was recorded in decades because of higher vegetable prices. By inserting values derived from assumed alternative scenarios into the Eq. (2) as a predictor, we predicted the final value of DALY rates until 2040 for each scenario. The DALY rates in the better and the moderate case scenarios mathematically converged to the almost same values in 2040 since both scenarios would meet the same level of vegetable consumption by 2040.

## Results

The sex- and age- group specific characteristics of the study are as shown in Table 1. The mean vegetable intake, the prevalence of current smokers and the prevalence of current alcohol drinkers declined over time, whereas SDI and the prevalence of obesity increased.

**Table 1:** Sex- and age- group specific DALY rates, SDI, and behavioural and metabolic risk predictor data

Year	Sex	GBD 2017 data				NHNS data				
		All ages DALY rates per 100,000 population [16]			SDI (%)	Number of NHNS participant	Mean age (SD)	Obesity (%)	Current smoker (%)	Current alcohol drinker (%)
		Cardiovascular diseases	Cancer	Diabetes and kidney diseases						
1990	Male	4649.6	5130.2	950.8	80.3	6182	47.4 (16.1)	22.3	53.1	52.1
	Female	3674.8	3280.9	828.1	80.3	7025	48.7 (16.8)	21.8	9.7	6.1
	Total population	4154.1	4190.1	888.4	80.3	13,207	48.1 (16.5)	22.0	28.5	26.0
1995	Male	4626.0	5832.0	1014.7	82.3	4976	47.8 (16.5)	23.9	52.7	54.4
	Female	3573.7	3578.8	839.4	82.3	5766	49.0 (17.3)	20.9	10.7	7.4
	Total population	4090.4	4685.2	925.5	82.3	10,742	48.4 (17.0)	22.2	28.2	27.0
2000	Male	4523.5	6185.2	1058.0	83.4	4513	50.1 (17.0)	26.8	47.4	50.8
	Female	3344.3	3745.2	844.3	83.4	5149	51.7 (17.5)	21.3	11.5	9.0
	Total population	3922.2	4940.9	949.0	83.4	9662	50.8 (17.2)	23.8	27.0	27.0
2005	Male	4646.2	6454.1	1097.6	84.4	3591	52.5 (17.5)	28.6	39.3	36.7
	Female	3313.6	3866.9	841.7	84.4	4155	54.5 (17.8)	22.0	11.4	7.4
	Total population	3964.9	5131.4	966.8	84.4	7746	53.6 (17.7)	24.9	24.3	20.9
2010	Male	4664.1	6549.9	1228.5	85.3	3740	53.9 (17.5)	30.4	32.2	35.4
	Female	3300.7	3954.5	934.3	85.3	4239	55.6 (17.8)	21.1	8.4	7.0
	Total population	3965.6	5220.2	1077.8	85.3	7746	54.8 (17.7)	25.3	19.5	20.3
2016	Male	4590.3	6449.7	1235.0	86.3	12,132	56.6 (17.6)	31.7	30.5	34.0
	Female	3400.8	3957.2	1003.6	86.3	14,010	58.1 (18.0)	21.3	7.6	7.5
	Total population	3980.4	5163.1	1116.4	86.3	26,142	57.4 (17.8)	25.9	18.2	19.8

*GBD* Global Burden of Disease study, *NHNS* National Health and Nutrition Survey of Japan, *DALYs* Disability-adjusted life years, *SDI* Socio-demographic index, *SD* Standard deviation. Note that we used data for each year, but the table lists only selected years

The *observed* (1995–2016) and *predicted* (2017–2040) vegetable consumption for the reference and different scenarios were as summarised in Table 2. There was a marked difference in the vegetable intake by sex- and age- groups. In particular, younger females (20–49 years old) had the lowest vegetable intake; conversely, older males (≥70 years old) were more likely to consume an increased amount of vegetables over time. A projected mean vegetable consumption of the total population showed a decreasing trend through 2040 to 237.7 g/day, which was lower than the lowest level of consumption observed in 2004 (Table 2). Similarly, the levels of vegetable consumption in the reference forecast were lower than those of the worse scenario forecasts for males aged 20–49 years, males aged 50–69 years, females aged 20–49 years, and females aged 50–69 years.

**Table 2:** Observed and predicted vegetable consumption (g/day) for reference forecast and three alternative scenarios

Age category		20–49											
Sex		Male				Female				Total population			
Scenario		R	1	2	3	R	1	2	3	R	1	2	3
Observed period^*1^	1995	269.8	269.8	269.8	269.8	252.8	252.8	252.8	252.8	263.9	263.9	263.9	263.9
	2000	279.1	279.1	279.1	279.1	254.7	254.7	254.7	254.7	264.5	264.5	264.5	264.5
	2004	228.2	228.2	228.2	228.2	207.4	207.4	207.4	207.4	217.0	217.0	217.0	217.0
	2010	245.2	245.2	245.2	245.2	217.6	217.6	217.6	217.6	228.9	228.9	228.9	228.9
	2016	240.0	240.0	240.0	240.0	217.1	217.1	217.1	217.1	229.5	229.5	229.5	229.5
Prediction period*1	2023	231.9	350.0	269.7	234.1	203.2	350.0	254.6	213.0	221.6	350.0	263.0	224.2
	2030	216.3	350.0	302.7	231.7	192.3	350.0	293.9	210.7	202.2	350.0	298.8	221.2
	2035	208.9	350.0	326.4	229.9	184.9	350.0	321.9	209.0	198.2	350.0	324.4	219.1
	2040	199.6	350.0	350.0	228.1	177.8	350.0	350.0	207.4	189.8	350.0	350.0	217.0
Age category		50–69											
Sex		Male				Female				Total population			
Scenario		R	1	2	3	R	1	2	3	R	1	2	3
Observed period*1	1995	311.2	311.2	311.2	311.2	289.5	289.5	289.5	289.5	299.0	299.0	299.0	299.0
	2000	324.8	324.8	324.8	324.8	324.9	324.9	324.9	324.9	327.5	327.5	327.5	327.5
	2004	267.0	267.0	267.0	267.0	259.1	259.1	259.1	259.1	262.8	262.8	262.8	262.8
	2010	277.7	277.7	277.7	277.7	278.5	278.5	278.5	278.5	280.9	280.9	280.9	280.9
	2016	273.4	273.4	273.4	273.4	267.3	267.3	267.3	267.3	271.2	271.2	271.2	271.2
Prediction period*1	2023	294.4	350.0	302.5	278.3	267.4	350.0	294.3	268.9	262.0	350.0	292.4	267.0
	2030	295.7	350.0	322.1	273.6	248.1	350.0	316.0	265.0	245.8	350.0	316.1	265.2
	2035	295.9	350.0	336.0	270.3	247.0	350.0	331.4	262.3	240.6	350.0	333.1	264.0
	2040	295.9	350.0	350.0	267.0	238.1	350.0	346.9	259.6	229.9	350.0	350.0	262.8
Age category		≥70											
Sex		Male				Female				Total population			
Scenario		R	1	2	3	R	1	2	3	R	1	2	3
Observed period*1	1995	260.9	260.9	260.9	260.9	250.7	250.7	250.7	250.7	253.8	253.8	253.8	253.8
	2000	324.3	324.3	324.3	324.3	303.0	303.0	303.0	303.0	301.9	301.9	301.9	301.9
	2004	252.2	252.2	252.2	252.2	240.0	240.0	240.0	240.0	245.3	245.3	245.3	245.3
	2010	283.1	283.1	283.1	283.1	272.8	272.8	272.8	272.8	276.8	276.8	276.8	276.8
	2016	287.0	287.0	287.0	287.0	272.5	272.5	272.5	272.5	279.1	279.1	279.1	279.1
Prediction period*1	2023	287.3	350.0	303.2	274.7	271.1	350.0	291.7	259.6	279.8	350.0	296.9	266.4
	2030	290.3	350.0	322.5	265.4	268.6	350.0	315.7	251.5	277.1	350.0	318.8	257.7
	2035	289.3	350.0	336.2	258.8	266.8	350.0	332.8	245.7	274.5	350.0	334.4	251.5
	2040	284.8	350.0	350.0	252.2	267.6	350.0	350.0	240.0	275.7	350.0	350.0	245.3
Age category		All ages											
Sex		Male				Female				Total population			
Scenario		R	1	2	3	R	1	2	3	R	1	2	3
Observed period*1	1995	284.1	284.1	284.1	284.1	265.8	265.8	265.8	265.8	272.2	272.2	272.2	272.2
	2000	302.4	302.4	302.4	302.4	287.0	287.0	287.0	287.0	294.2	294.2	294.2	294.2
	2004	247.9	247.9	247.9	247.9	233.7	233.7	233.7	233.7	240.2	240.2	240.2	240.2
	2010	264.9	264.9	264.9	264.9	255.5	255.5	255.5	255.5	259.9	259.9	259.9	259.9
	2016	266.6	266.6	266.6	266.6	253.2	253.2	253.2	253.2	259.3	259.3	259.3	259.3
Prediction period*1	2023	256.5	350.0	284.9	255.1	251.9	350.0	282.3	248.4	252.4	350.0	285.3	253.2
	2030	246.4	350.0	311.7	252.1	236.4	350.0	310.2	242.3	246.2	350.0	311.9	247.9
	2035	239.6	350.0	330.8	250.0	235.2	350.0	330.1	238.0	241.9	350.0	331.0	244.1
	2040	230.9	350.0	350.0	247.9	229.3	350.0	350.0	233.7	237.7	350.0	350.0	240.2

^*^^1^Observed: 1990–2016; predicted: 2017–2040

R: reference forecast; 1: better case scenario; 2: moderate case scenario; 3: worse case scenario

### Future trends of DALY rates for cardiovascular disease, cancer, and diabetes and kidney diseases

#### Cardiovascular disease

**Fig. 1:**
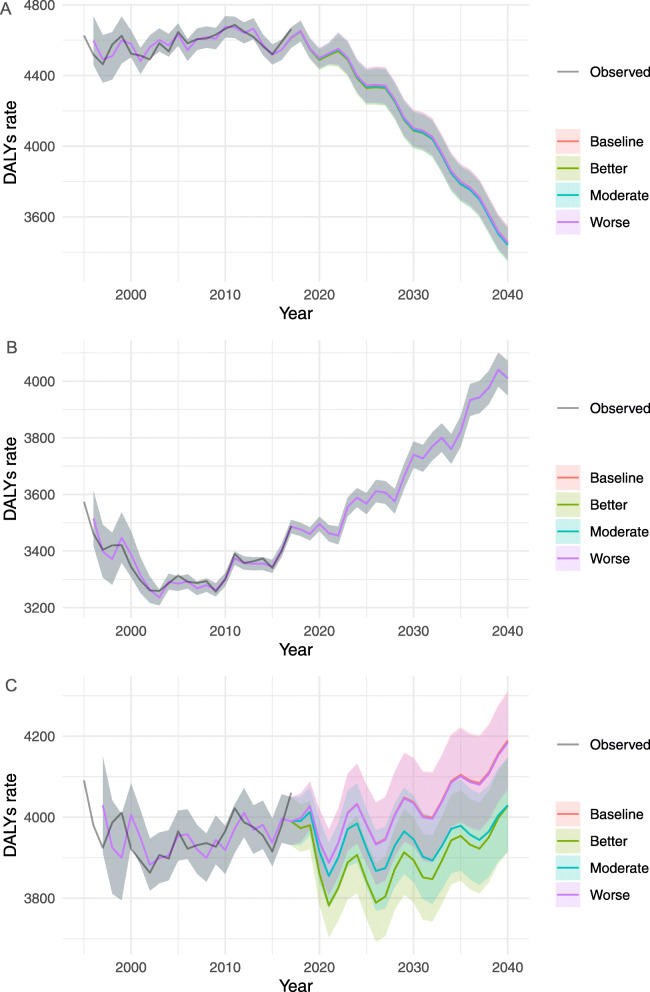
Observed and projected all-age DALY rates (per 100,000 population) for cardiovascular diseases, 1990–2040: (**a**) male, (**b**) female, and (**c**) total population. The black lines represent the observed values, and the pink lines and the grey areas before 2016 represent the smoothed lines and their projection intervals

The best combination of parameters (p, d, and q) in the ARIMA models derived from Eq. (2) with corresponding AIC values are provided in Supplementary Table 1. Estimated DALY rates of CVDs for all ages by sex and scenarios are shown in Fig. 1 and for age-sex specific groups are shown in Supplementary figure 1, 2 and 3, respectively. In all four scenarios, the projected DALY rates during 2017–2040 continued to increase among the total population, all females, and females aged ≥70, whereas the rate decreased for the rest of the groups. The difference in the trends between the age group-specific estimates and all-age estimates is that the latter was greatly affected by the ageing of the population.

The DALY rates of sex-age specific groups for the cardiovascular disease under each scenario between 2017 and 2040 are presented in Supplementary Table 2. In the reference forecast, the greatest decline in DALY rates was forecasted in the 20–49 years old age group, with an average decline rate of 35.6, 40.4 and 30.9% for males, females and sex-combined during 2017–2040, respectively. Significant differences between scenarios were observed among younger females (20–49 years old) with un-overlapped 95% prediction intervals of DALY rates (95% PIs) between 298.8 (290.5–307.4) for reference and 274.8 (267.2–282.7) for both better and moderate case scenarios with a decline rate of 8.0%. Similarly, significant differences were shown in the 50–69 years old group between 2027 and 2033 with the most decline of 85.8 (84.6–87.1) per 100,000. Meanwhile, there was an increasing trend for all-age DALY rates in contrast to the trend for each age group, suggesting that the population ageing may influence future DALY rates.

#### Cancer

**Fig. 2:**
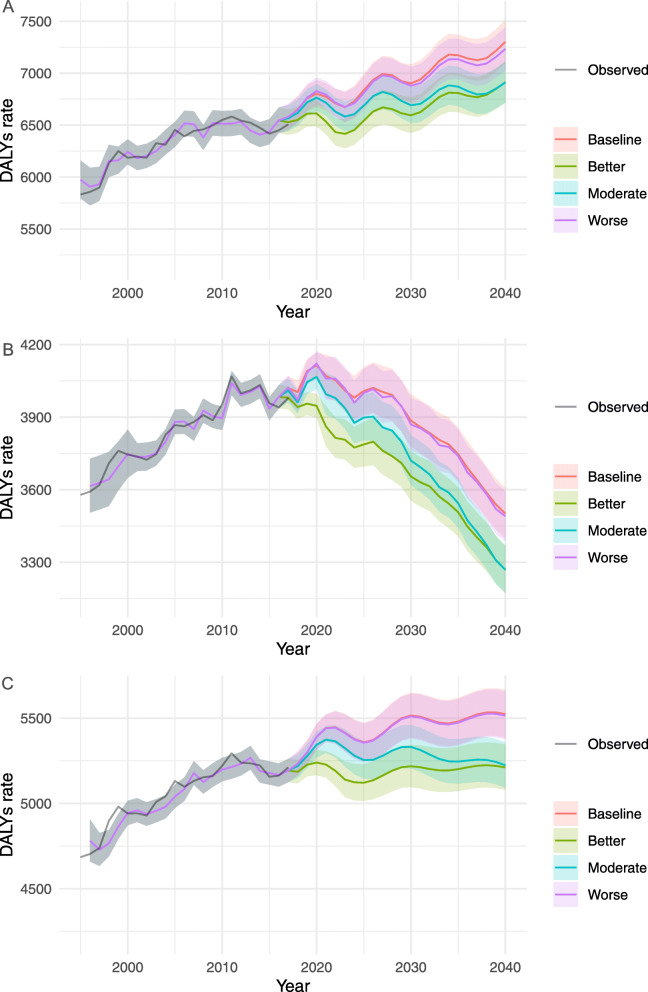
Observed and projected all-ages DALY rates (per 100,000 population) for cancer, 1990–2040: (**a**) male, (**b**) female, and (**c**) total population. The black lines represent the observed values, and the pink lines and the grey areas before 2016 represent the smoothed lines and their projection intervals

The DALY rates of cancer for by sex and scenarios are shown in Fig. 2. In all scenarios, there were upward trends in the DALY rates among males and the total population, while the rate decreased among females. In contrast to the trend for each age-group, there is an upward trend for all-age DALY rates, suggesting that the ageing of the population may have significant effects on cancer. Significant differences between scenarios were observed among the total population with un-overlapped 95% PIs of DALY rates (95% PIs) between 5510.8 (5372.1–5653.2) for reference and 5201.5 (5070.5–5335.9) for both better and moderate case scenarios. The forecasted DALY rates in the better case scenario dropped sharply in 2023 compared to the reference forecast. Overall, a decline of 5.6% in DALY rates was forecasted for the total population in both the better and the moderate case scenarios in comparison to the reference forecast. The DALY rates by sex-age specific groups for cancer under each scenario between 2017 and 2040 are presented in Supplementary Table 3.

DALY rates by age- and sex- groups show a continuous decline to 2040 in most groups except for females aged 50–69 (Supplementary figures 4, 5 and 6). In the reference forecast, the greatest expected decline in DALY rates was observed in the group aged 20–49, with an average decline of 40.2, 44.2 and 11.4% for males, females and sex-combined groups during 2017–2040, respectively. Significant differences between the reference and the better and moderate case scenarios were found in females aged 20–49, females aged 50–69, and both sexes aged 50–69. Compared to the reference forecast, the largest decline rate was observed in females aged 20–49 (− 14.3%), while the most change in DALY rates was observed in males aged 70 and older with a decline of 852.7 (828.1–878.0) per 100,000 from 2017 to 2040 in both the better and the moderate case scenarios.

#### Diabetes and kidney diseases

**Fig. 3:**
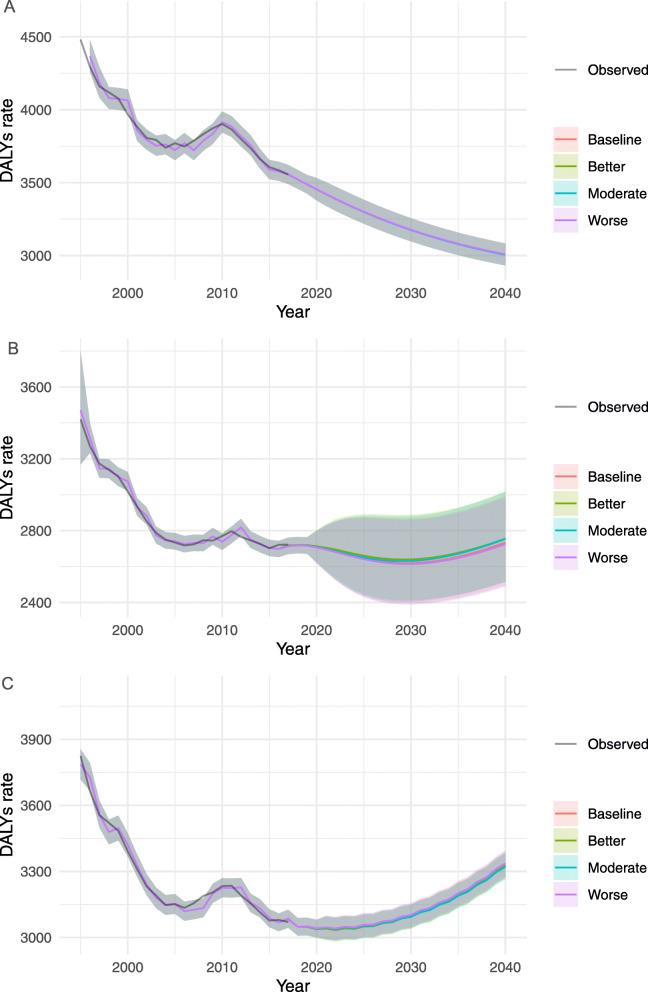
Observed and projected all ages DALY rates (per 100,000 population) for diabetes and kidney diseases, 1990–2040: (**a**) male, (**b**) female, and (**c**) total population. The black lines represent the observed values, and the pink lines and the grey areas before 2016 represent the smoothed lines and their projection intervals

Figure 3 show the trends of DALY rates for DKD through 2040 by sex and different scenarios. The DALY rates increased through 2040 among females and the total population, while the rate decreased among males. Significant differences between the reference and the better and moderate case scenarios were observed in both sex-specific groups with un-overlapped 95% PIs of DALY rates (95% PIs) between 1965.9 (1928.3–2004.4) and 1804.2 (1769.6–1839.5) for males, and DALY rates (95% PIs) between 2765.0 (2727.3–2803.3) and 2386.0 (2353.4–2419.0) for females, that is, 8.2 and 13.7% of decline rate in males and females, respectively. The DALY rates by sex-age specific groups for diabetes and kidney diseases under each scenario between 2017 and 2040 are presented in Supplementary Table 4.

DALY rates by age- and sex- groups were plotted in Supplementary figures 7, 8 and 9. In the reference forecast, a significant upward trend in DALY rates was observed for most age- and sex-specific groups, except in males aged 50–69 and ≥ 70. In particular, the DALY rates in 2040 showed 1.5 times increase than that of 2017 for the younger group (20–49). The largest decline was observed in females aged 20–49 (− 35.7%), while the most change in the DALY rates was observed in females aged 50–69 with a decline of 213.1 (210.7–215.5) per 100,000 from 2017 to 2040.

## Discussion

Based on a future forecast of DALY rates and probable scenarios, we estimated a ‘case for change’ in the level of vegetable consumption on the disease burden attributable to the different disease through 2040. In Japan, the current level of vegetable consumption is far from the target of the government guidelines and will not be achieved by 2040 if the current trend in vegetable consumption continues. Our analysis indicates that increased vegetable consumption would lead to a significant reduction in the burden of CVDs, cancer and DKDs.

In the reference forecast, all-age DALY rates were forecasted to continue increasing for CVDs, cancer, and DKDs, while age group-specific DALY rates showed different trends. There was no difference between the reference forecast and the worse case scenario in the DALY rates for all sex and age groups of the three diseases with overlapping PIs given that there was little difference in the vegetable intake of those scenarios. On the other hand, one of the greatest gaps of the estimated DALY rates between the reference and scenario forecasts was found among females aged 20–49, specifically between the reference forecast and both better and moderate case scenarios for all three diseases. This is in part due to the projection of younger females consuming fewer vegetables in the future, which also resulted in the widest gap between the estimated and the ideal level of vegetable consumption. By targeting this group for a public health intervention related to vegetable consumption, we can expect the greatest impact of reducing disease burden in Japan.

In accordance with previous studies, our study showed the benefits against CVDs by increased vegetable intake among younger female (20–49 years) and 50–69 years old age group [20, 21]. Various nutrients in vegetables also demonstrate to protect against CVDs through a variety of mechanisms; a decrease of atherosclerosis in the blood vessel and blood pressure, and a lowering of the risk of oxidative damage [22]. However, increasing vegetable intake was expected to benefit to a limited age group in this study. Another study suggested that 400 ± 30 g/ day of vegetable intake was set as a minimum theoretical risk of exposure to low vegetable intake against CVDs, indicating that our scenarios’ vegetable consumption level might be slightly short for obtaining significant health benefits [23].

By increasing the vegetable intake at the total population level, the DALY rates for cancer decreased by 5.6%. Our estimates suggest 50–69 years old age group was expected to have the greatest benefits on reducing the burden from cancers if this age group consumed the ideal level of vegetables. Around 70% of total cancer cases were reported from over 65 years [24], indicating a vigorous intervention to the middle-aged population may effectively help to reduce the burden from cancers. A report from World Cancer Research Fund suggests that low vegetable intake is associated with specific cancer risks including aerodigestive cancer (probable), and lung, breast, and colorectal cancers (limited suggestive) [25]. Vegetables are also protective against certain types of cancers by blocking the action of and/ or suppressing carcinogens. For example, cruciferous vegetable and types of yellow vegetables contain protease inhibitors, isothiocyanates and carotenoids prevent the initial formation of cancer and/or oxidative damage of deoxyribonucleic acid [6, 22].

Similar to the scenarios in cancer, DALY rates of DKD can possibility decrease by 8% for males and 13% for females. Although very limited evidence suggests a possible link between vegetable intake and type 2 diabetes in Japan [26], there is a growing body of evidence on various type of diets that may prevent adult-onset diabetes [27].

Internationally, Japan was ranked 62nd in the world by vegetable intake per capita in 2011 [28]. China was ranked first in the world, consuming over three times more vegetables than Japan on a per capita basis. The tendencies that the younger population compared to older people, and females compared to males consume less vegetable were, however, similar to other countries [6, 29]. WHO’s report found barriers to increasing vegetable intake as social, environmental, and economic reasons, including a lack of knowledge of the recommended dietary intake, personal and family eating habits, limited availability of vegetables, a lack of required vegetable for cooking due to less time for preparation, and a lack of intervention for promoting healthy eating [30, 31]. Another study indicated that low socio-economic status consumed fewer quantities and varieties of vegetables in relation to vegetable prices [32]. These factors influence food choice and dietary intake across individuals, countries and cultures.

The barriers above mentioned are relevant in the Japanese context as well. Japanese society today faces a general increase in preference for westernised diet and lifestyle, an increase in the availability of cooked and processed foods, and an increase the vegetable prices due to a consumption tax increase and repeated natural disasters in the recent years [33, 34]. In addition, there is a reduced vegetable supply attributable to the ageing population of farmers and decrease in farming areas from urbanisation, resulting in the number of farmer decrease over the last decades [33, 35]. It should be noted that climate change is also the main driver to influence the reduction in vegetable supply [36]. In Japan, the multiple natural disasters damaged farms and led to a soaring price of vegetables in 2004, resulting in the lowest vegetable consumption in all-age-sex groups [37].

The government enacted the Dietary Guidelines for the Japanese to enhance the balanced diet and promote nutritional education at the community level [38]. This guideline was effective to translate the knowledge on the daily recommended diet to the general population. However, to tackle the diseases associated with low vegetable consumption, a combination of social, environmental and economic barriers must be addressed. For instance, it would be important to invest in policies which drive an increase in consumption first, including interventions to increase supply and access to vegetable. A report from Australia simulated that the high vegetable intake would reduce CVDs and some cancers would result in long-term benefits by saving government health expenditure of about one million dollars and translating it into producer return [39]. As such, a comprehensive package and frames of policies are needed to encourage people to eat adequate quantities of vegetables. These include improvements in the food environment, food systems, and behaviours change communication across the life course.

Several limitations should be noted as in any forecasting study. First, our models may not have addressed enough to adjust possible risk predictors associated with the target disease. For example, the level of physical exercise and socio-economic status, which are well-known factors to influence the disease burden of NCDs, were unavailable. However, we put SDI into the models as a substitute for individual SES data. Second, although a change in vegetable intake and health outcomes may be explained in part by health performance, social determinants and environmental factors, including the price change of vegetables [33], were not assessed. Other dietary factors, which may also have influenced the change in vegetable consumption [40], were not included in the models to avoid over-adjustment. Third, while DALYs is an excellent measure to capture disease burden at the population level and a piece of key information for policymaking, DALYs itself cannot indicate what kind of and how much investment are needed to improve health outcomes [41]. Lastly, because the dietary data from the NHNS was based on a weighted single-day dietary record, our analysis may not have captured the real trend of long-term nor seasonal changes in dietary patterns. Despite some limitations, our study uses the best available data that represents the Japanese population’s dietary pattern over time. Simple models like ours have advantages in allowing for a prompt exploration of dietary risk factors and relevant disease burden forecasts.

## Conclusions

This study provided a ‘case for change’ of the level of vegetable intake accompanying with associated disease burden by different scenarios, allowing for a better understanding of possible target populations and a certain qualification of the range of policy impacts. A key challenge remains to translate these findings into public health policy. We believe our findings may open-up a discussion on effective, targeted interventions for public health challenges attributable to low vegetable diet in Japan.

## Supplementary Information

**Additional file 1: Figure S1.** Observed and projected DALY rate (per 100,000 population) in the 20–49 age group for cardiovascular diseases for reference forecast and three alternative scenarios, 1990–2040: (A) male, (B) female, and (C) total population. The black lines represent the observed values, and the pink lines and the grey areas before 2016 represent the smoothed lines and their projection intervals. **Figure S2.** Observed and projected DALY rates (per 100,000 population) in the 50–69 age group for cardiovascular diseases for reference forecast and three alternative scenarios, 1990–2040: (A) male, (B) female, and (C) total population. The black lines represent the observed values, and the pink lines and the grey areas before 2016 represent the smoothed lines and their projection intervals. **Figure S3.** Observed and projected DALY rates (per 100,000 population) in the ≥70 age group for cardiovascular diseases for reference forecast and three alternative scenarios, 1990–2040: (A) male, (B) female, and (C) total population. The black lines represent the observed values, and the pink lines and the grey areas before 2016 represent the smoothed lines and their projection intervals. **Figure S4.** Observed and projected DALY rates (per 100,000 population) for cancer in the 20–49 age group for reference forecast and three alternative scenarios, 1990–2040: (A) male, (B) female, and (C) total population. The black lines represent the observed values, and the pink lines and the grey areas before 2016 represent the smoothed lines and their projection intervals. **Figure S5.** Observed and projected DALY rates (per 100,000 population) in the 50–69 age group for cancer for reference forecast and three alternative scenarios, 1990–2040: (A) male, (B) female, and (C) total population. The black lines represent the observed values, and the pink lines and the grey areas before 2016 represent the smoothed lines and their projection intervals. **Figure S6.** Observed and projected DALY rates (per 100,000 population) in the ≥70 age group for cancer for reference forecast and three alternative scenarios, 1990–2040: (A) male, (B) female, and (C) total population. The black lines represent the observed values, and the pink lines and the grey areas before 2016 represent the smoothed lines and their projection intervals. **Figure S7.** Observed and projected DALY rates (per 100,000 population) in the 20–49 age group for diabetes and kidney diseases for reference forecast and three alternative scenarios, 1990–2040: (A) male, (B) female, and (C) total population. The black lines represent the observed values, and the pink lines and the grey areas before 2016 represent the smoothed lines and their projection intervals. **Figure S8.** Observed and projected DALY rates (per 100,000 population) in the 50–69 age group for diabetes and kidney diseases for reference forecast and three alternative scenarios, 1990–2040: (A) male, (B) female, and (C) total population. The black lines represent the observed values, and the pink lines and the grey areas before 2016 represent the smoothed lines and their projection intervals. **Figure S9.** Observed and projected DALY rates (per 100,000 population) in the ≥70 age group for diabetes and kidney diseases for reference forecast and three alternative scenarios, 1990–2040: (A) male, (B) female, and (C) total population. The black lines represent the observed values, and the pink lines and the grey areas before 2016 represent the smoothed lines and their projection intervals. **Table S1.** Estimated sets of parameters in ARIMA (p, d, q) and Akaike Information Criteria (AIC). **Table S2.** Predicted DALY rates (95% prediction intervals) for cardiovascular disease by age groups, sex (male, female), and total population, 2017–2040. **Table S3.** Predicted DALY rates (95% prediction intervals) for cancer by age groups, sex (male, female), and total population, 2017–2040. **Table S4.** Predicted DALY rates (95% prediction intervals) for diabetes and kidney diseases by age groups, sex (male, female), and total population, 2017–2040.

## Data Availability

All data generated or analysed during this study are included in this published article [and its supplementary information files].

## Abbreviations

AIC: Akaike’s Information Criterion
ARIMA: Autoregressive integrated moving average
BMI: Body mass index
CVDs: Cardiovascular diseases
DALYs: Disability-adjusted life years
DKDs: Diabetes and kidney diseases
GBD: Global Burden of Disease
NHNS: National Health and Nutrition Survey
PIs: Prediction intervals
SDI: Socio-demographic index
WHO: World Health Organization
