# Supplementary material for: Projections of disability-adjusted life years for major diseases due to a change in vegetable intake in 2017–2040 in Japan
Source: BMC Public Health. 2021 Apr 21;21:770. doi: 10.1186/s12889-021-10772-2 (PMC8061031; doi:10.1186/s12889-021-10772-2)
Supplement: Supplementary file 1 — Additional file 1: Figure S1. Observed and projected DALY rate (per 100,000 population) in the 20–49 age group for cardiovascular diseases for reference forecast and three alternative scenarios, 1990–2040: (A) male, (B) female, and (C) total population. The black lines represent the observed values, and the pink lines and the grey areas before 2016 represent the smoothed lines and their projection intervals. Figure S2. Observed and projected DALY rates (per 100,000 population) in the 50–69 age group for cardiovascular diseases for reference forecast and three alternative scenarios, 1990–2040: (A) male, (B) female, and (C) total population. The black lines represent the observed values, and the pink lines and the grey areas before 2016 represent the smoothed lines and their projection intervals. Figure S3. Observed and projected DALY rates (per 100,000 population) in the ≥70 age group for cardiovascular diseases for reference forecast and three alternative scenarios, 1990–2040: (A) male, (B) female, and (C) total population. The black lines represent the observed values, and the pink lines and the grey areas before 2016 represent the smoothed lines and their projection intervals. Figure S4. Observed and projected DALY rates (per 100,000 population) for cancer in the 20–49 age group for reference forecast and three alternative scenarios, 1990–2040: (A) male, (B) female, and (C) total population. The black lines represent the observed values, and the pink lines and the grey areas before 2016 represent the smoothed lines and their projection intervals. Figure S5. Observed and projected DALY rates (per 100,000 population) in the 50–69 age group for cancer for reference forecast and three alternative scenarios, 1990–2040: (A) male, (B) female, and (C) total population. The black lines represent the observed values, and the pink lines and the grey areas before 2016 represent the smoothed lines and their projection intervals. Figure S6. Observed and projected DALY rates (pe [file 12889_2021_10772_MOESM1_ESM.docx]

**Supplementary information**

Projections of disability-adjusted life years for major diseases due to a change in vegetable intake in 2017-2040 in Japan

**Authors:** Shiori Tanaka^1,2#†^, Daisuke Yoneoka^1,3,4#^, Aya Ishizuka^1^, Ueda Peter^1,5^, Keiji Nakamura^6,7^, Hisayuki Uneyama^7^, Naoki Hayashi^7,8^, Kenji Shibuya^1,9^, Shuhei Nomura^1,4^

# Shared first authorship

**Affiliations:**

1. Department of Global Health Policy, Graduate School of Medicine, The University of Tokyo, Tokyo, Japan
2. Epidemiology and Prevention Group, Center for Public Health Sciences, National Cancer Center, 5-1-1 Tsukiji, Chuo-ku, Tokyo 104-0045, Japan
3. Graduate School of Public Health, St. Luke's International University, Tokyo, Japan
4. Department of Health Policy and Management, School of Medicine, Keio University, Tokyo, Japan
5. Clinical Epidemiology Division, Department of Medicine, Solna, Karolinska Institutet, Stockholm, Sweden
6. Graduate School of Environmental and Information Studies, Tokyo City University, Yokohama, Japan
7. Ajinomoto Co., Inc., Tokyo, Japan
8. Department of Applied Biological Chemistry, Graduate School of Agriculture and Life Sciences,
9. The University of Tokyo, Tokyo, JapanInstitute for Population Health, King's College London, London, UK; Department of Global Health Policy

**Supplementary figure 1: Observed and projected 20–49 age group DALYs rate (per 100,000) for cardiovascular diseases for reference forecast and three alternative scenarios, 1995–2040: (A) male, (B) female, and (C) total population**. The black lines represent the observed values, and the pink lines and the grey areas before 2016 represent the smoothed lines and their projection intervals.


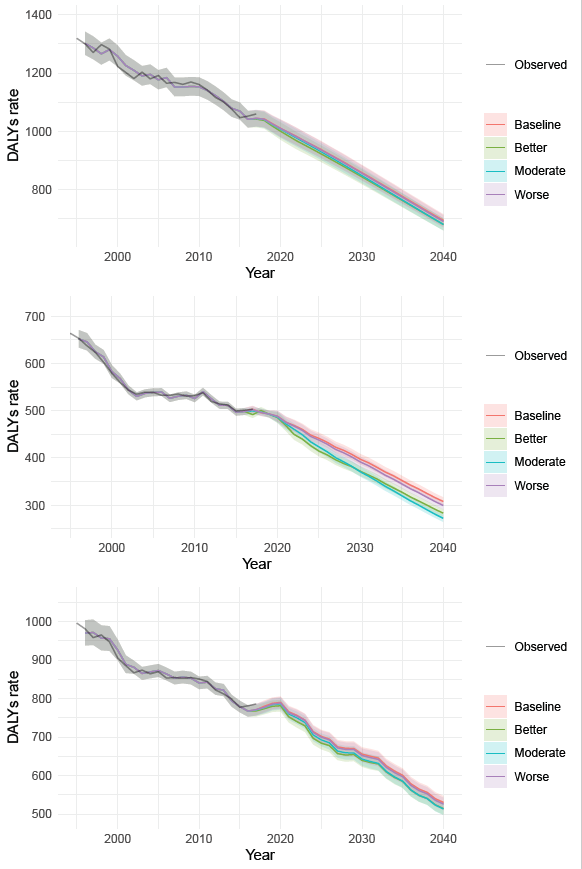


C

B

A

**Supplementary figure 2: Observed and projected 50–69 age group DALYs rate (per 100,000) for cardiovascular diseases for reference forecast and three alternative scenarios, 1990–2040: (A) male, (B) female, and (C) total population**. The black lines represent the observed values, and the pink lines and the grey areas before 2016 represent the smoothed lines and their projection intervals.

A


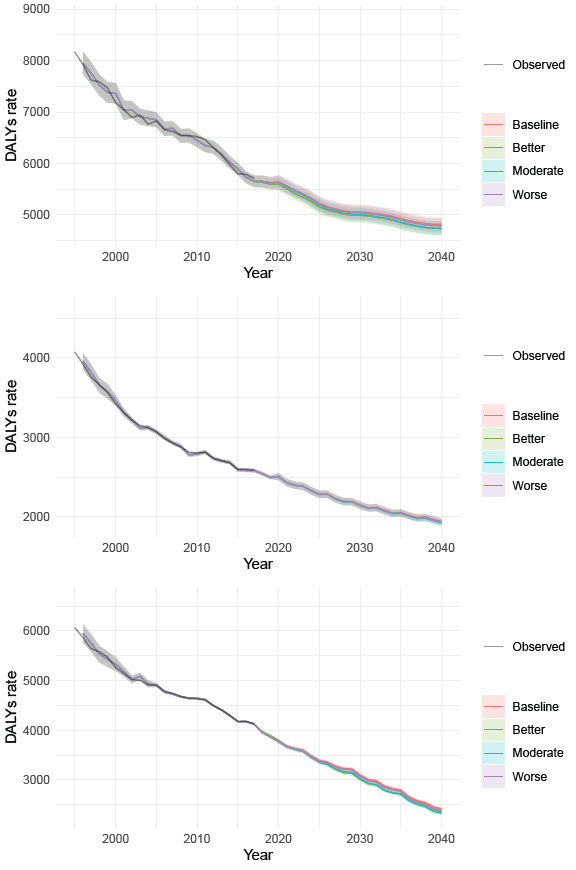


C

B

**Supplementary figure 3: Observed and projected ≥70** **age group DALYs rate (per 100,000) for cardiovascular diseases for reference forecast and three alternative scenarios, 1990–2040: (A) male, (B) female, and (C) total population**. The black lines represent the observed values, and the pink lines and the grey areas before 2016 represent the smoothed lines and their projection intervals.

A


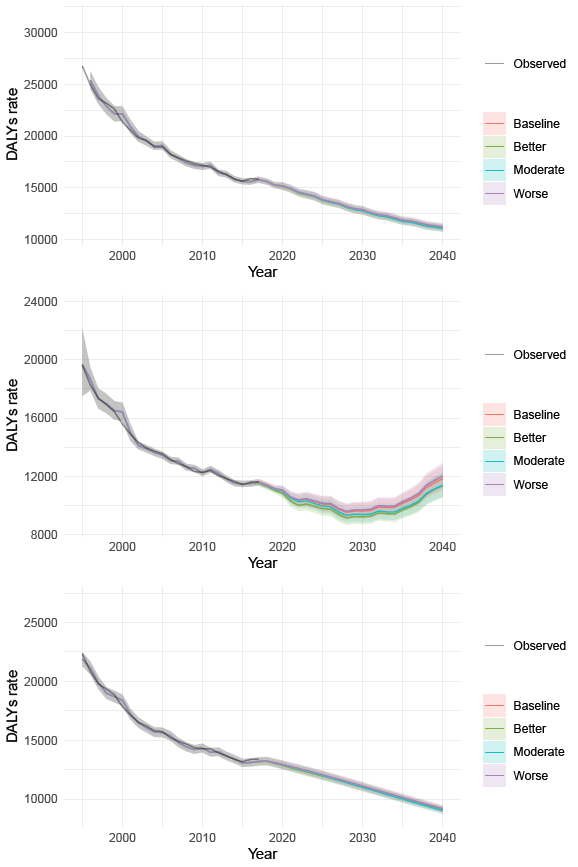


C

B

**Supplementary figure 4: Observed and projected 20–49 age group DALYs rate (per 100,000) for cancer for reference forecast and three alternative scenarios, 1990–2040: (A) male, (B) female, and (C) total population**. The black lines represent the observed values, and the pink lines and the grey areas before 2016 represent the smoothed lines and their projection intervals.

A


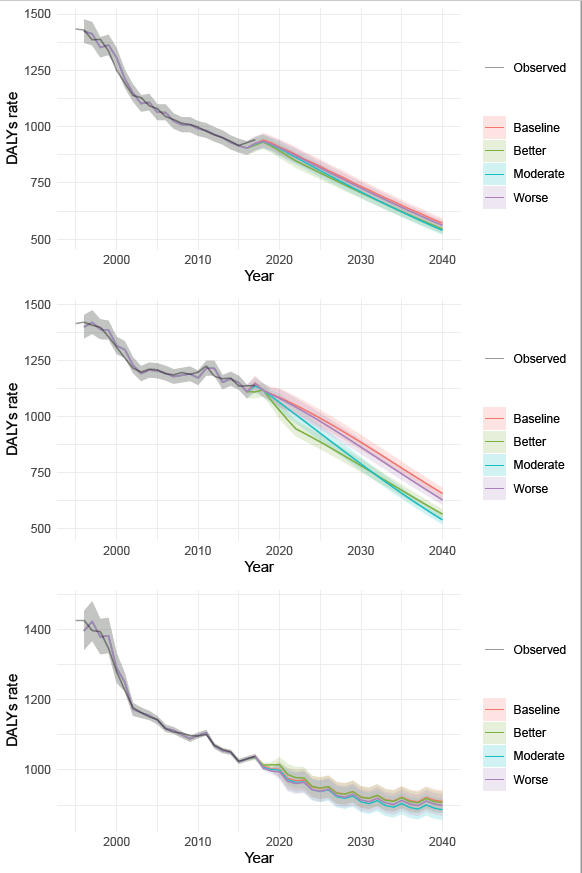


C

B

**Supplementary figure 5: Observed and projected 50–69 age group DALYs rate (per 100,000) for cancer for reference forecast and three alternative scenarios, 1990–2040: (A) male, (B) female, and (C) total population**. The black lines represent the observed values, and the pink lines and the grey areas before 2016 represent the smoothed lines and their projection intervals.

A


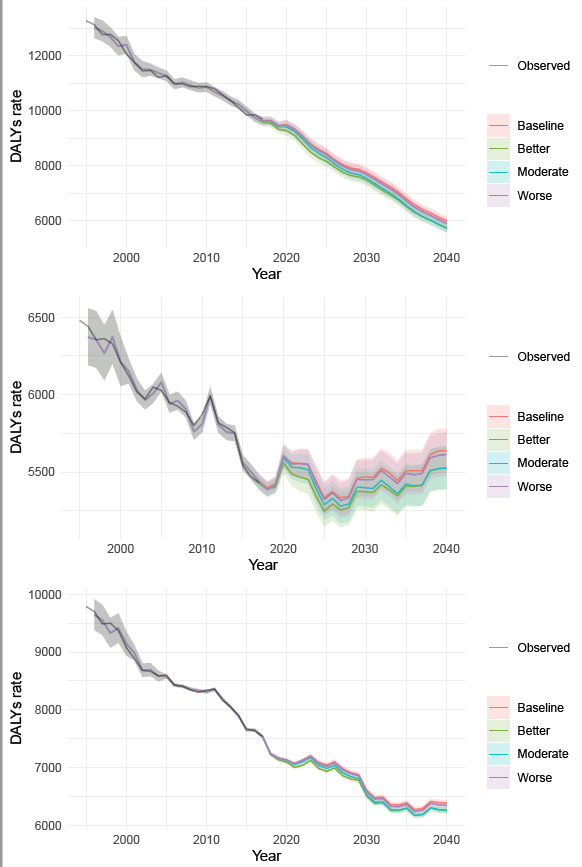


C

B

**Supplementary figure 6: Observed and projected ≥70** **age group DALYs rate (per 100,000) for cancer for reference forecast and three alternative scenarios, 1990–2040: (A) male, (B) female, and (C) total population**. The black lines represent the observed values, and the pink lines and the grey areas before 2016 represent the smoothed lines and their projection intervals.


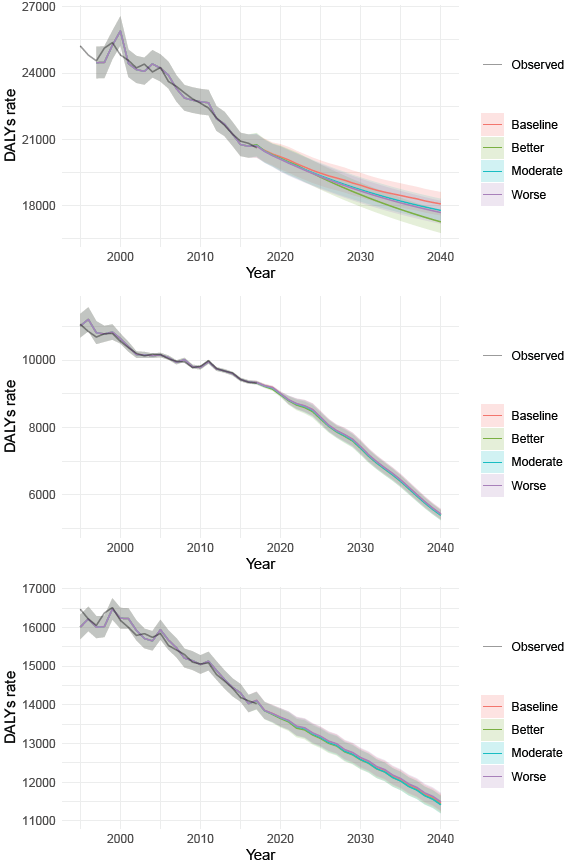


C

B

A

**Supplementary figure 7: Observed and projected 20–49 age group DALYs rate (per 100,000) for diabetes and kidney diseases for reference forecast and three alternative scenarios, 1990–2040: (A) male, (B) female, and (C) total population**. The black lines represent the observed values, and the pink lines and the grey areas before 2016 represent the smoothed lines and their projection intervals.


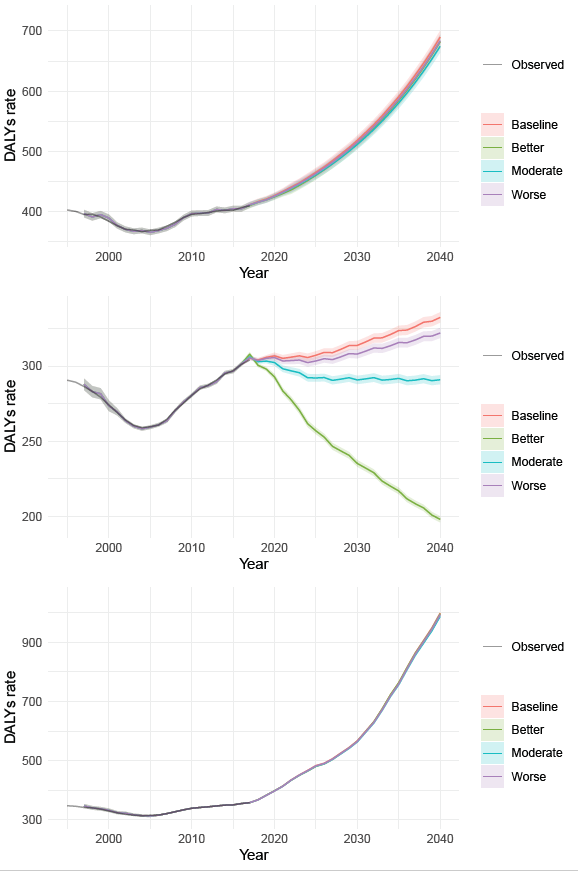


C

B

A

**Supplementary figure 8: Observed and projected 50–69 age group DALYs rate (per 100,000) for diabetes and kidney diseases for reference forecast and three alternative scenarios, 1990–2040: (A) male, (B) female, and (C) total population**. The black lines represent the observed values, and the pink lines and the grey areas before 2016 represent the smoothed lines and their projection intervals.


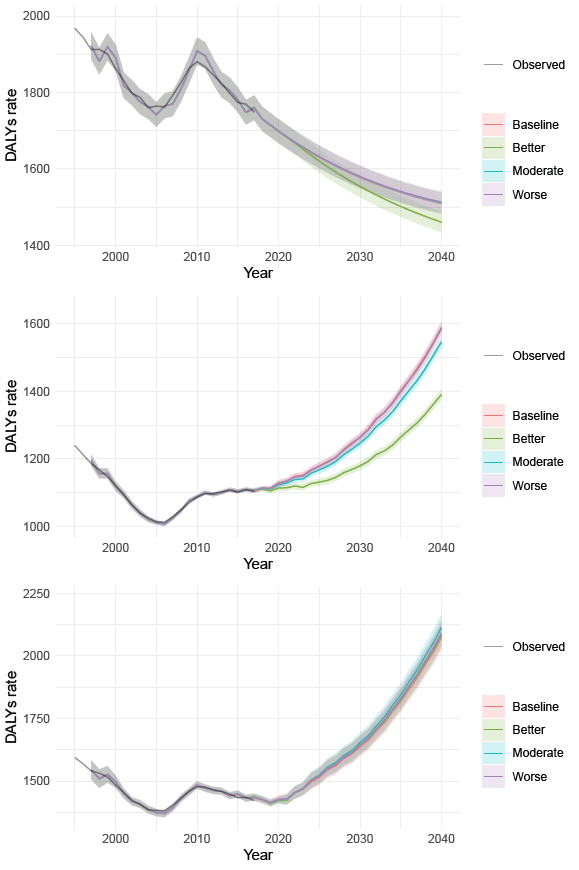


C

B

A

**Supplementary figure 9: Observed and projected ≥70** **age group DALYs rate (per 100,000) for diabetes and kidney diseases for reference forecast and three alternative scenarios, 1990–2040: (A) male, (B) female, and (C) total population**. The black lines represent the observed values, and the pink lines and the grey areas before 2016 represent the smoothed lines and their projection intervals.


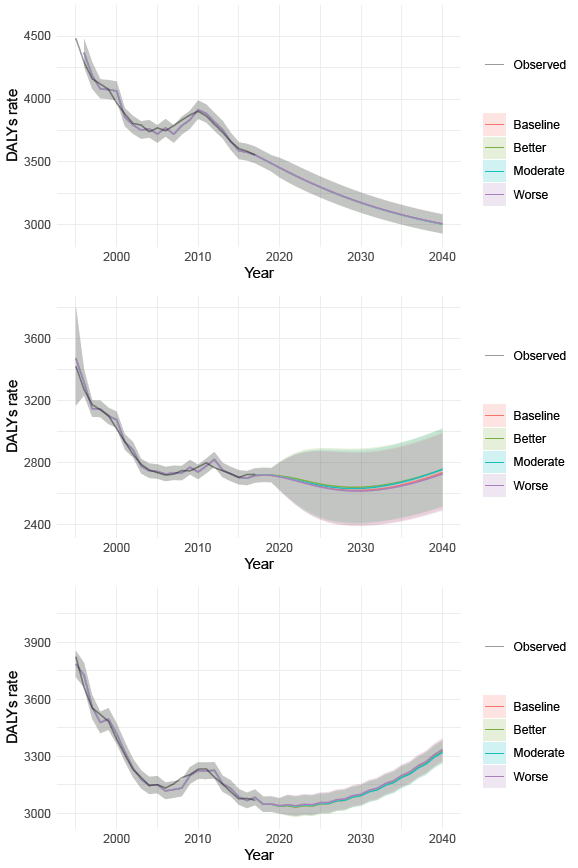


C

B

A

**Supplemental Table 1: Estimated sets of parameters in ARIMA(p,d,q) and Akaike Information Criteria (AIC)**

| Disease | Age group | Sex | p | d | q | AIC |
| --- | --- | --- | --- | --- | --- | --- |
| Cardiovascular diseases | 20–49 | Male | 1 | 1 | 1 | -106.6 |
|  |  | Female | 4 | 1 | 1 | -124.0 |
|  |  | Total population | 5 | 1 | 0 | -112.5 |
|  | 50–69 | Male | 9 | 1 | 0 | -139.5 |
|  |  | Female | 3 | 1 | 1 | -139.5 |
|  |  | Total population | 8 | 1 | 2 | -149.1 |
|  | ≥70 | Male | 5 | 1 | 3 | -104.9 |
|  |  | Female | 7 | 0 | 0 | -115.7 |
|  |  | Total population | 1 | 1 | 1 | -138.2 |
|  | All ages | Male | 4 | 1 | 2 | -128.1 |
|  |  | Female | 6 | 1 | 3 | -138.4 |
|  |  | Total population | 3 | 1 | 1 | -124.4 |
| Cancer | 20–49 | Male | 1 | 1 | 0 | -101.2 |
|  |  | Female | 0 | 1 | 1 | -108.3 |
|  |  | Total population | 10 | 2 | 0 | -182.3 |
|  | 50–69 | Male | 9 | 1 | 0 | -139.5 |
|  |  | Female | 4 | 1 | 3 | -139.5 |
|  |  | Total population | 8 | 1 | 0 | -158.5 |
|  | ≥70 | Male | 2 | 2 | 1 | -102.8 |
|  |  | Female | 8 | 1 | 2 | -154.1 |
|  |  | Total population | 5 | 1 | 1 | -154.5 |
|  | All ages | Male | 6 | 0 | 0 | -121.8 |
|  |  | Female | 4 | 1 | 2 | -126.0 |
|  |  | Total population | 11 | 0 | 0 | -161.6 |
| Diabetes and kidney diseases | 20–49 | Male | 0 | 2 | 4 | -134.9 |
|  |  | Female | 5 | 2 | 1 | -162.8 |
|  |  | Total population | 4 | 1 | 3 | -138.6 |
|  | 50–69 | Male | 3 | 2 | 3 | -157.1 |
|  |  | Female | 7 | 2 | 2 | -146.9 |
|  |  | Total population | 3 | 2 | 3 | -133.2 |
|  | ≥70 | Male | 1 | 1 | 0 | -126.3 |
|  |  | Female | 2 | 0 | 0 | -138.1 |
|  |  | Total population | 1 | 2 | 3 | -162.4 |
|  | All ages | Male | 1 | 2 | 2 | -128.5 |
|  |  | Female | 5 | 2 | 1 | -145.7 |
|  |  | Total population | 0 | 2 | 3 | -140.6 |

**Supplementary Table 2: Predicted DALY rates (95% prediction intervals) for cardiovascular disease by age groups, sex (male, female), and total population, 2017–2040.**

| Year | Reference | Better scenario | Moderate scenario | Worse scenario |
| --- | --- | --- | --- | --- |
| Male, 20-49y | |  |  |  |
| 2017 | 1042.6 (1014.6－1071.4) | 1037.2 (1009.3－1065.8) | 1040.0 (1012.0－1068.7) | 1041.1 (1013.1－1069.9) |
| 2018 | 1026.3 (998.7－1054.6) | 1017.9 (990.6－1046.0) | 1022.9 (995.4－1051.1) | 1024.9 (997.4－1053.2) |
| 2019 | 1010.1 (980.7－1040.4) | 1000.3 (971.2－1030.3) | 1007.0 (977.7－1037.2) | 1009.9 (980.5－1040.1) |
| 2020 | 996.1 (966.5－1026.6) | 983.4 (954.2－1013.5) | 991.6 (962.1－1022.0) | 995.1 (965.6－1025.6) |
| 2021 | 981.7 (952.4－1012.0) | 966.8 (937.9－996.6) | 976.3 (947.1－1006.4) | 980.5 (951.1－1010.7) |
| 2022 | 966.0 (937.1－995.9) | 952.1 (923.6－981.5) | 960.9 (932.1－990.5) | 965.7 (936.7－995.5) |
| 2023 | 952.0 (923.4－981.4) | 937.2 (909.1－966.2) | 945.3 (916.9－974.5) | 950.6 (922.1－980.0) |
| 2024 | 937.3 (909.2－966.2) | 922.1 (894.5－950.6) | 929.5 (901.6－958.2) | 935.4 (907.3－964.3) |
| 2025 | 921.3 (893.6－949.7) | 906.8 (879.6－934.8) | 913.5 (886.1－941.7) | 919.9 (892.3－948.3) |
| 2026 | 906.1 (878.9－934.1) | 891.3 (864.5－918.8) | 897.3 (870.4－925.0) | 904.2 (877.1－932.1) |
| 2027 | 890.7 (864.0－918.2) | 875.5 (849.3－902.6) | 880.9 (854.5－908.1) | 888.2 (861.6－915.7) |
| 2028 | 874.0 (847.8－901.0) | 859.6 (833.8－886.1) | 864.4 (838.5－891.1) | 872.1 (846.0－899.1) |
| 2029 | 858.0 (832.3－884.5) | 843.5 (818.2－869.6) | 847.7 (822.3－873.9) | 855.9 (830.2－882.3) |
| 2030 | 842.1 (816.8－868.1) | 827.3 (802.5－852.9) | 831.0 (806.1－856.7) | 839.5 (814.3－865.4) |
| 2031 | 825.2 (800.5－850.7) | 811.0 (786.7－836.0) | 814.2 (789.8－839.3) | 823.0 (798.3－848.4) |
| 2032 | 808.8 (784.6－833.8) | 794.6 (770.8－819.2) | 797.3 (773.4－821.9) | 806.4 (782.2－831.3) |
| 2033 | 792.8 (769.0－817.2) | 778.2 (754.9－802.2) | 780.4 (757.0－804.5) | 789.8 (766.1－814.2) |
| 2034 | 775.8 (752.6－799.8) | 761.7 (738.9－785.3) | 763.5 (740.6－787.1) | 773.1 (749.9－797.0) |
| 2035 | 759.3 (736.5－782.7) | 745.3 (723.0－768.3) | 746.7 (724.3－769.7) | 756.5 (733.8－779.9) |
| 2036 | 743.1 (720.8－766.1) | 728.9 (707.1－751.4) | 729.9 (708.0－752.4) | 739.9 (717.7－762.7) |
| 2037 | 726.3 (704.6－748.8) | 712.6 (691.2－734.6) | 713.1 (691.8－735.2) | 723.3 (701.7－745.7) |
| 2038 | 709.9 (688.6－731.8) | 696.3 (675.4－717.8) | 696.5 (675.7－718.1) | 706.9 (685.7－728.7) |
| 2039 | 693.9 (673.1－715.3) | 680.2 (659.8－701.2) | 680.1 (659.7－701.1) | 690.5 (669.8－711.9) |
| 2040 | 677.5 (657.2－698.5) | 664.1 (644.2－684.7) | 663.7 (643.8－684.2) | 674.3 (654.1－695.1) |
| Male, 50-69y | |  |  |  |
| 2017 | 5654.6 (5543.5－5768.0) | 5636.1 (5525.3－5749.1) | 5648.2 (5537.2－5761.5) | 5652.9 (5541.8－5766.3) |
| 2018 | 5627.9 (5517.4－5740.6) | 5595.9 (5486.0－5707.9) | 5616.6 (5506.4－5729.1) | 5624.8 (5514.4－5737.4) |
| 2019 | 5644.1 (5516.2－5774.9) | 5601.0 (5474.1－5730.8) | 5628.6 (5501.1－5759.0) | 5639.7 (5511.9－5770.4) |
| 2020 | 5566.6 (5438.7－5697.5) | 5512.8 (5386.2－5642.5) | 5547.0 (5419.5－5677.4) | 5560.9 (5433.2－5691.7) |
| 2021 | 5480.2 (5354.3－5609.0) | 5411.1 (5286.8－5538.3) | 5454.8 (5329.5－5583.1) | 5472.9 (5347.2－5601.6) |
| 2022 | 5414.3 (5289.7－5541.9) | 5345.8 (5222.8－5471.7) | 5385.8 (5261.8－5512.7) | 5406.0 (5281.6－5533.4) |
| 2023 | 5326.6 (5190.8－5466.0) | 5259.1 (5125.1－5396.7) | 5295.2 (5160.2－5433.7) | 5317.3 (5181.8－5456.4) |
| 2024 | 5221.9 (5088.3－5358.9) | 5154.5 (5022.7－5289.8) | 5187.4 (5054.7－5323.5) | 5211.6 (5078.3－5348.3) |
| 2025 | 5159.2 (5026.6－5295.3) | 5092.0 (4961.1－5226.3) | 5121.7 (4990.1－5256.8) | 5147.9 (5015.6－5283.6) |
| 2026 | 5119.8 (4988.2－5254.8) | 5055.1 (4925.2－5188.5) | 5080.0 (4949.5－5214.0) | 5107.6 (4976.3－5242.3) |
| 2027 | 5080.9 (4950.2－5215.1) | 5016.1 (4887.0－5148.6) | 5038.2 (4908.5－5171.3) | 5067.7 (4937.3－5201.5) |
| 2028 | 5061.3 (4926.9－5199.3) | 4995.0 (4862.4－5131.2) | 5015.1 (4882.0－5151.8) | 5046.9 (4913.0－5184.6) |
| 2029 | 5061.3 (4926.8－5199.4) | 4993.6 (4860.9－5129.9) | 5011.6 (4878.4－5148.4) | 5045.8 (4911.7－5183.5) |
| 2030 | 5050.2 (4916.0－5188.2) | 4980.9 (4848.5－5116.9) | 4997.1 (4864.2－5133.5) | 5033.6 (4899.8－5171.1) |
| 2031 | 5027.6 (4894.0－5164.9) | 4955.2 (4823.4－5090.5) | 4970.6 (4838.5－5106.4) | 5009.8 (4876.6－5146.6) |
| 2032 | 5004.2 (4871.1－5140.9) | 4929.6 (4798.5－5064.3) | 4943.7 (4812.3－5078.8) | 4985.2 (4852.6－5121.4) |
| 2033 | 4971.6 (4838.0－5109.0) | 4895.9 (4764.3－5031.1) | 4908.2 (4776.2－5043.7) | 4951.6 (4818.6－5088.4) |
| 2034 | 4927.8 (4794.9－5064.3) | 4851.1 (4720.3－4985.5) | 4861.5 (4730.4－4996.2) | 4906.8 (4774.5－5042.8) |
| 2035 | 4888.3 (4756.5－5023.8) | 4811.1 (4681.4－4944.4) | 4819.4 (4689.4－4953.0) | 4866.5 (4735.2－5001.3) |
| 2036 | 4858.3 (4727.3－4993.0) | 4781.3 (4652.4－4913.8) | 4786.9 (4657.8－4919.6) | 4835.5 (4705.1－4969.5) |
| 2037 | 4834.1 (4703.7－4968.1) | 4757.2 (4628.9－4889.0) | 4760.2 (4631.9－4892.2) | 4810.4 (4680.7－4943.7) |
| 2038 | 4819.9 (4689.4－4953.9) | 4742.3 (4614.0－4874.2) | 4743.2 (4614.9－4875.1) | 4795.1 (4665.4－4928.5) |
| 2039 | 4818.0 (4687.4－4952.4) | 4739.7 (4611.1－4871.8) | 4738.5 (4609.9－4870.6) | 4792.3 (4662.3－4925.9) |
| 2040 | 4821.1 (4690.2－4955.6) | 4741.5 (4612.8－4873.8) | 4738.4 (4609.8－4870.6) | 4794.2 (4664.1－4928.0) |
| Male, ≥70y | |  |  |  |
| 2017 | 15574.5 (15289.6－15864.6) | 15602.3 (15316.9－15892.9) | 15607.0 (15321.5－15897.7) | 15609.8 (15324.3－15900.6) |
| 2018 | 15230.6 (14952.2－15514.1) | 15242.8 (14964.3－15526.6) | 15262.5 (14983.6－15546.7) | 15273.9 (14994.7－15558.2) |
| 2019 | 15120.4 (14775.4－15473.6) | 15123.0 (14777.9－15476.2) | 15160.8 (14814.8－15514.8) | 15182.5 (14836.0－15537.1) |
| 2020 | 14853.2 (14507.5－15207.0) | 14870.0 (14524.0－15224.3) | 14910.8 (14563.8－15266.0) | 14935.6 (14588.1－15291.4) |
| 2021 | 14504.3 (14166.2－14850.4) | 14468.3 (14131.0－14813.5) | 14530.0 (14191.3－14876.7) | 14567.3 (14227.8－14915.0) |
| 2022 | 14336.2 (13958.9－14723.8) | 14297.6 (13921.2－14684.1) | 14350.4 (13972.7－14738.4) | 14397.3 (14018.3－14786.5) |
| 2023 | 14153.2 (13776.8－14539.9) | 14114.0 (13738.6－14499.6) | 14154.0 (13777.6－14540.8) | 14208.0 (13830.1－14596.2) |
| 2024 | 13781.7 (13415.0－14158.3) | 13749.2 (13383.4－14124.9) | 13780.1 (13413.5－14156.7) | 13841.9 (13473.7－14220.2) |
| 2025 | 13598.9 (13232.0－13975.9) | 13555.3 (13189.6－13931.1) | 13586.1 (13219.6－13962.7) | 13660.4 (13291.9－14039.1) |
| 2026 | 13419.4 (13056.9－13791.9) | 13406.2 (13044.0－13778.3) | 13422.2 (13059.7－13794.8) | 13501.3 (13136.6－13876.1) |
| 2027 | 13104.0 (12741.7－13476.7) | 13094.7 (12732.6－13467.1) | 13106.1 (12743.7－13478.8) | 13193.7 (12828.8－13568.9) |
| 2028 | 12901.5 (12532.7－13281.1) | 12878.4 (12510.3－13257.4) | 12886.4 (12518.0－13265.6) | 12983.3 (12612.1－13365.3) |
| 2029 | 12784.6 (12418.6－13161.3) | 12766.9 (12401.5－13143.1) | 12767.4 (12402.0－13143.6) | 12871.8 (12503.4－13251.1) |
| 2030 | 12524.8 (12165.0－12895.2) | 12503.1 (12143.9－12872.9) | 12494.0 (12135.1－12863.5) | 12603.1 (12241.0－12975.9) |
| 2031 | 12324.2 (11969.7－12689.2) | 12286.3 (11932.9－12650.2) | 12278.4 (11925.2－12642.1) | 12397.8 (12041.2－12765.0) |
| 2032 | 12214.7 (11860.2－12579.8) | 12187.0 (11833.3－12551.3) | 12170.1 (11816.9－12533.9) | 12295.3 (11938.5－12662.8) |
| 2033 | 12000.3 (11643.6－12367.9) | 11981.3 (11625.2－12348.3) | 11958.2 (11602.8－12324.5) | 12089.1 (11729.8－12459.4) |
| 2034 | 11779.7 (11427.0－12143.2) | 11761.4 (11409.3－12124.4) | 11735.8 (11384.5－12098.0) | 11873.8 (11518.4－12240.3) |
| 2035 | 11693.8 (11343.5－12054.9) | 11673.4 (11323.7－12033.9) | 11644.3 (11295.5－12003.9) | 11790.2 (11437.0－12154.3) |
| 2036 | 11517.9 (11171.9－11874.6) | 11510.1 (11164.3－11866.5) | 11471.6 (11126.9－11826.9) | 11620.9 (11271.8－11980.8) |
| 2037 | 11330.3 (10989.5－11681.6) | 11304.1 (10964.1－11654.6) | 11265.7 (10926.9－11615.0) | 11422.5 (11079.0－11776.7) |
| 2038 | 11237.3 (10894.4－11590.9) | 11210.2 (10868.1－11563.0) | 11167.0 (10826.3－11518.5) | 11330.1 (10984.4－11686.7) |
| 2039 | 11112.7 (10769.9－11466.3) | 11089.8 (10747.7－11442.7) | 11040.5 (10700.0－11391.9) | 11208.5 (10862.8－11565.2) |
| 2040 | 10919.2 (10582.5－11266.7) | 10895.6 (10559.5－11242.3) | 10843.0 (10508.5－11188.0) | 11015.8 (10676.0－11366.3) |
| Male, all ages | |  |  |  |
| 2017 | 4652.8 (4594.9－4711.5) | 4649.0 (4591.1－4707.5) | 4651.2 (4593.4－4709.8) | 4652.1 (4594.3－4710.8) |
| 2018 | 4553.6 (4497.0－4611.0) | 4548.8 (4492.2－4606.1) | 4552.1 (4495.4－4609.4) | 4553.4 (4496.8－4610.8) |
| 2019 | 4496.9 (4440.2－4554.3) | 4487.8 (4431.3－4545.1) | 4495.1 (4438.4－4552.4) | 4498.0 (4441.4－4555.5) |
| 2020 | 4525.3 (4465.3－4586.2) | 4514.9 (4455.0－4575.6) | 4522.7 (4462.7－4583.5) | 4526.1 (4466.0－4586.9) |
| 2021 | 4551.4 (4460.8－4643.8) | 4538.4 (4448.1－4630.6) | 4547.4 (4456.9－4639.8) | 4551.4 (4460.8－4643.8) |
| 2022 | 4501.2 (4407.6－4596.8) | 4489.7 (4396.3－4585.1) | 4496.8 (4403.3－4592.4) | 4501.0 (4407.4－4596.6) |
| 2023 | 4398.3 (4306.4－4492.1) | 4384.7 (4293.1－4478.2) | 4392.1 (4300.3－4485.8) | 4397.2 (4305.4－4491.1) |
| 2024 | 4343.8 (4247.8－4441.8) | 4329.0 (4233.4－4426.8) | 4336.4 (4240.7－4434.3) | 4342.4 (4246.6－4440.5) |
| 2025 | 4347.6 (4247.1－4450.5) | 4333.3 (4233.1－4435.8) | 4340.1 (4239.7－4442.8) | 4346.7 (4246.2－4449.6) |
| 2026 | 4341.9 (4239.8－4446.4) | 4328.7 (4227.0－4432.9) | 4333.9 (4232.0－4438.2) | 4340.8 (4238.8－4445.3) |
| 2027 | 4264.9 (4164.7－4367.6) | 4252.1 (4152.2－4354.5) | 4256.4 (4156.3－4358.8) | 4263.7 (4163.5－4366.3) |
| 2028 | 4160.9 (4059.3－4265.0) | 4147.9 (4046.6－4251.7) | 4152.2 (4050.8－4256.1) | 4160.2 (4058.6－4264.3) |
| 2029 | 4101.9 (4001.4－4204.8) | 4087.7 (3987.6－4190.3) | 4092.1 (3991.9－4194.7) | 4100.8 (4000.4－4203.7) |
| 2030 | 4088.2 (3986.5－4192.6) | 4073.8 (3972.4－4177.8) | 4077.4 (3975.9－4181.4) | 4086.5 (3984.8－4190.8) |
| 2031 | 4052.9 (3950.5－4158.0) | 4039.4 (3937.4－4144.1) | 4041.7 (3939.6－4146.4) | 4051.0 (3948.7－4156.0) |
| 2032 | 3962.4 (3861.9－4065.4) | 3948.9 (3848.9－4051.6) | 3950.5 (3850.4－4053.2) | 3960.2 (3859.8－4063.2) |
| 2033 | 3859.9 (3760.1－3962.4) | 3845.8 (3746.3－3948.0) | 3847.5 (3748.0－3949.7) | 3857.7 (3757.9－3960.2) |
| 2034 | 3798.3 (3699.9－3899.2) | 3784.1 (3686.2－3884.7) | 3785.8 (3687.8－3886.4) | 3796.5 (3698.2－3897.4) |
| 2035 | 3766.9 (3668.1－3868.5) | 3753.2 (3654.7－3854.3) | 3754.0 (3655.5－3855.2) | 3765.0 (3666.2－3866.5) |
| 2036 | 3711.6 (3613.5－3812.4) | 3698.4 (3600.6－3798.8) | 3698.2 (3600.5－3798.6) | 3709.3 (3611.3－3810.0) |
| 2037 | 3614.6 (3518.6－3713.2) | 3601.6 (3506.0－3699.9) | 3601.1 (3505.5－3699.3) | 3612.4 (3516.5－3710.9) |
| 2038 | 3517.3 (3422.8－3614.5) | 3503.7 (3409.5－3600.5) | 3503.3 (3409.1－3600.1) | 3515.0 (3420.5－3612.1) |
| 2039 | 3455.7 (3362.8－3551.1) | 3441.6 (3349.1－3536.7) | 3441.1 (3348.6－3536.1) | 3453.1 (3360.2－3548.4) |
| 2040 | 3411.8 (3319.0－3507.1) | 3398.3 (3305.9－3493.2) | 3397.0 (3304.6－3491.9) | 3409.1 (3316.5－3504.4) |
| Female, 20-49y | |  |  |  |
| 2017 | 495.9 (488.8－503.0) | 500.4 (493.3－507.7) | 497.2 (490.1－504.4) | 496.3 (489.2－503.5) |
| 2018 | 492.8 (485.8－500.0) | 492.4 (485.4－499.6) | 492.3 (485.3－499.4) | 492.9 (485.9－500.1) |
| 2019 | 488.1 (476.7－499.8) | 485.4 (474.0－497.0) | 486.4 (475.0－498.0) | 488.1 (476.7－499.8) |
| 2020 | 475.8 (464.4－487.6) | 468.5 (457.2－480.0) | 472.1 (460.7－483.7) | 475.4 (464.0－487.1) |
| 2021 | 468.8 (456.9－481.0) | 449.8 (438.4－461.4) | 460.6 (448.9－472.5) | 467.4 (455.5－479.5) |
| 2022 | 460.4 (448.6－472.5) | 439.8 (428.6－451.4) | 449.1 (437.5－460.9) | 458.3 (446.5－470.3) |
| 2023 | 448.4 (436.5－460.5) | 426.0 (414.8－437.6) | 434.1 (422.6－445.9) | 445.6 (433.8－457.7) |
| 2024 | 441.4 (429.7－453.4) | 414.5 (403.5－425.8) | 423.3 (412.1－434.9) | 437.8 (426.2－449.7) |
| 2025 | 433.0 (421.5－444.9) | 406.4 (395.6－417.5) | 413.0 (402.0－424.2) | 428.9 (417.5－440.6) |
| 2026 | 422.7 (411.1－434.5) | 396.2 (385.4－407.3) | 400.8 (389.8－412.0) | 418.1 (406.7－429.9) |
| 2027 | 415.9 (404.5－427.6) | 388.0 (377.4－398.9) | 391.6 (380.9－402.6) | 410.8 (399.6－422.3) |
| 2028 | 407.1 (396.0－418.6) | 381.2 (370.8－391.9) | 381.8 (371.4－392.6) | 401.8 (390.8－413.0) |
| 2029 | 397.2 (386.3－408.4) | 371.9 (361.7－382.3) | 370.6 (360.4－381.0) | 391.5 (380.7－402.5) |
| 2030 | 389.6 (378.9－400.6) | 363.6 (353.6－373.9) | 361.3 (351.3－371.5) | 383.4 (372.9－394.3) |
| 2031 | 380.1 (369.7－390.9) | 355.4 (345.6－365.5) | 351.0 (341.3－360.9) | 373.7 (363.4－384.3) |
| 2032 | 370.2 (360.0－380.7) | 345.4 (335.9－355.2) | 339.9 (330.5－349.5) | 363.5 (353.5－373.8) |
| 2033 | 362.1 (352.0－372.4) | 336.5 (327.2－346.1) | 330.4 (321.2－339.8) | 355.0 (345.1－365.1) |
| 2034 | 352.4 (342.7－362.5) | 327.7 (318.6－337.0) | 320.1 (311.2－329.2) | 345.1 (335.5－354.9) |
| 2035 | 342.9 (333.4－352.7) | 317.9 (309.0－326.9) | 309.7 (301.1－318.5) | 335.3 (326.0－344.9) |
| 2036 | 334.6 (325.3－344.2) | 309.2 (300.6－318.0) | 300.5 (292.1－309.0) | 326.7 (317.7－336.0) |
| 2037 | 325.2 (316.2－334.5) | 300.5 (292.2－309.1) | 290.7 (282.6－299.0) | 317.2 (308.4－326.2) |
| 2038 | 316.2 (307.4－325.2) | 291.4 (283.3－299.7) | 281.1 (273.3－289.1) | 307.9 (299.4－316.7) |
| 2039 | 307.9 (299.3－316.7) | 283.2 (275.3－291.3) | 272.3 (264.7－280.1) | 299.5 (291.1－308.0) |
| 2040 | 298.8 (290.5－307.4) | 274.8 (267.2－282.7) | 263.1 (255.8－270.6) | 290.3 (282.2－298.6) |
| Female, 50-69y | |  |  |  |
| 2017 | 2545.2 (2523.4－2567.1) | 2544.8 (2523.0－2566.7) | 2543.0 (2521.2－2564.9) | 2542.3 (2520.5－2564.2) |
| 2018 | 2495.7 (2474.3－2517.2) | 2497.7 (2476.3－2519.3) | 2496.0 (2474.6－2517.5) | 2495.4 (2474.0－2517.0) |
| 2019 | 2505.8 (2463.6－2548.6) | 2507.8 (2465.6－2550.7) | 2507.2 (2465.1－2550.1) | 2507.3 (2465.1－2550.2) |
| 2020 | 2431.1 (2386.4－2476.6) | 2429.9 (2385.2－2475.4) | 2429.5 (2384.9－2475.0) | 2429.8 (2385.1－2475.3) |
| 2021 | 2398.7 (2354.1－2444.1) | 2397.6 (2353.1－2443.1) | 2398.4 (2353.8－2443.8) | 2399.2 (2354.6－2444.7) |
| 2022 | 2385.6 (2338.7－2433.4) | 2384.0 (2337.1－2431.7) | 2384.2 (2337.4－2432.0) | 2386.0 (2339.1－2433.8) |
| 2023 | 2332.9 (2286.7－2380.0) | 2329.2 (2283.1－2376.3) | 2327.9 (2281.8－2374.9) | 2330.0 (2283.9－2377.1) |
| 2024 | 2285.9 (2240.6－2332.0) | 2283.6 (2238.4－2329.8) | 2281.4 (2236.2－2327.5) | 2284.2 (2239.0－2330.4) |
| 2025 | 2286.2 (2240.2－2333.2) | 2283.7 (2237.7－2330.7) | 2281.3 (2235.4－2328.2) | 2285.2 (2239.1－2332.2) |
| 2026 | 2231.1 (2186.1－2277.1) | 2227.3 (2182.4－2273.2) | 2223.7 (2178.8－2269.5) | 2228.0 (2183.0－2273.8) |
| 2027 | 2194.1 (2149.6－2239.5) | 2192.3 (2147.9－2237.7) | 2188.0 (2143.6－2233.2) | 2192.8 (2148.4－2238.2) |
| 2028 | 2193.0 (2148.1－2238.8) | 2190.7 (2145.9－2236.4) | 2186.3 (2141.6－2232.0) | 2192.2 (2147.4－2238.0) |
| 2029 | 2150.4 (2106.4－2195.3) | 2146.5 (2102.5－2191.3) | 2141.1 (2097.2－2185.8) | 2147.3 (2103.4－2192.2) |
| 2030 | 2115.6 (2072.0－2160.2) | 2112.9 (2069.3－2157.3) | 2106.8 (2063.4－2151.2) | 2113.6 (2070.0－2158.1) |
| 2031 | 2119.2 (2075.2－2164.1) | 2115.4 (2071.5－2160.2) | 2109.4 (2065.6－2154.0) | 2117.1 (2073.2－2161.9) |
| 2032 | 2082.6 (2039.4－2126.8) | 2077.2 (2034.1－2121.2) | 2070.3 (2027.3－2114.2) | 2078.4 (2035.3－2122.4) |
| 2033 | 2049.6 (2006.8－2093.4) | 2045.7 (2002.9－2089.4) | 2038.2 (1995.5－2081.7) | 2046.7 (2003.9－2090.5) |
| 2034 | 2054.4 (2011.2－2098.4) | 2049.8 (2006.7－2093.8) | 2042.3 (1999.4－2086.1) | 2051.8 (2008.7－2095.8) |
| 2035 | 2022.3 (1979.8－2065.7) | 2016.6 (1974.2－2059.9) | 2008.3 (1966.1－2051.4) | 2018.1 (1975.7－2061.5) |
| 2036 | 1990.7 (1948.5－2033.8) | 1986.5 (1944.4－2029.5) | 1977.6 (1935.7－2020.4) | 1987.8 (1945.7－2030.8) |
| 2037 | 1997.6 (1955.2－2041.0) | 1992.5 (1950.1－2035.7) | 1983.6 (1941.4－2026.7) | 1994.7 (1952.3－2038.0) |
| 2038 | 1970.0 (1928.1－2012.8) | 1963.4 (1921.6－2006.1) | 1953.8 (1912.2－1996.3) | 1965.3 (1923.4－2008.0) |
| 2039 | 1942.0 (1900.4－1984.6) | 1936.6 (1895.1－1979.1) | 1926.4 (1885.1－1968.7) | 1938.2 (1896.7－1980.7) |
| 2040 | 1949.6 (1907.7－1992.4) | 1943.2 (1901.4－1985.9) | 1933.1 (1891.5－1975.5) | 1945.7 (1903.9－1988.4) |
| Female, ≥70y | |  |  |  |
| 2017 | 11413.4 (11208.2－11622.4) | 11315.5 (11112.1－11522.8) | 11360.6 (11156.3－11568.6) | 11382.7 (11178.0－11591.1) |
| 2018 | 11156.4 (10955.8－11360.7) | 11028.5 (10830.1－11230.4) | 11119.5 (10919.6－11323.1) | 11165.4 (10964.6－11369.9) |
| 2019 | 11002.9 (10698.1－11316.3) | 10835.9 (10535.8－11144.6) | 10977.6 (10673.5－11290.3) | 11050.6 (10744.5－11365.4) |
| 2020 | 10533.9 (10159.6－10921.9) | 10291.1 (9925.5－10670.2) | 10484.3 (10111.8－10870.5) | 10585.6 (10209.6－10975.5) |
| 2021 | 10321.4 (9896.1－10764.9) | 10000.5 (9588.5－10430.2) | 10254.7 (9832.3－10695.4) | 10390.0 (9961.9－10836.4) |
| 2022 | 10386.8 (9928.1－10866.6) | 10100.0 (9654.0－10566.6) | 10313.4 (9858.0－10789.9) | 10461.2 (9999.3－10944.5) |
| 2023 | 10245.4 (9786.9－10725.4) | 9937.3 (9492.6－10402.9) | 10131.3 (9678.0－10606.0) | 10300.2 (9839.2－10782.7) |
| 2024 | 10090.7 (9612.2－10593.1) | 9780.4 (9316.6－10267.4) | 9957.4 (9485.2－10453.1) | 10147.1 (9665.9－10652.3) |
| 2025 | 10066.8 (9564.7－10595.3) | 9741.6 (9255.7－10253.0) | 9909.2 (9414.9－10429.4) | 10124.0 (9619.0－10655.5) |
| 2026 | 9723.9 (9218.3－10257.3) | 9376.2 (8888.6－9890.5) | 9542.2 (9046.0－10065.6) | 9780.1 (9271.5－10316.6) |
| 2027 | 9527.1 (9018.2－10064.8) | 9137.1 (8648.9－9652.8) | 9322.2 (8824.2－9848.4) | 9593.6 (9081.1－10135.1) |
| 2028 | 9612.0 (9095.9－10157.3) | 9232.1 (8736.4－9755.8) | 9399.5 (8894.9－9932.8) | 9692.0 (9171.6－10241.8) |
| 2029 | 9605.4 (9083.7－10157.1) | 9214.5 (8714.0－9743.8) | 9373.4 (8864.3－9911.8) | 9689.1 (9162.8－10245.6) |
| 2030 | 9648.4 (9120.6－10206.7) | 9256.0 (8749.7－9791.6) | 9401.8 (8887.5－9945.8) | 9739.6 (9206.9－10303.2) |
| 2031 | 9885.7 (9338.2－10465.3) | 9482.3 (8957.2－10038.2) | 9614.2 (9081.8－10177.9) | 9979.8 (9427.1－10564.9) |
| 2032 | 9849.4 (9297.1－10434.5) | 9432.1 (8903.2－9992.4) | 9555.1 (9019.3－10122.7) | 9942.5 (9385.0－10533.1) |
| 2033 | 9871.0 (9312.2－10463.4) | 9403.6 (8871.3－9967.9) | 9542.7 (9002.4－10115.3) | 9965.2 (9401.1－10563.3) |
| 2034 | 10154.7 (9571.2－10773.8) | 9682.8 (9126.4－10273.1) | 9806.5 (9243.0－10404.4) | 10260.0 (9670.4－10885.5) |
| 2035 | 10387.2 (9753.8－11061.7) | 9905.8 (9301.8－10549.0) | 10018.5 (9407.6－10669.1) | 10504.2 (9863.6－11186.3) |
| 2036 | 10685.6 (10004.3－11413.3) | 10209.7 (9558.7－10904.9) | 10302.1 (9645.3－11003.7) | 10819.8 (10129.9－11556.6) |
| 2037 | 11248.5 (10500.9－12049.5) | 10782.0 (10065.3－11549.7) | 10843.4 (10122.6－11615.5) | 11401.9 (10644.0－12213.7) |
| 2038 | 11559.5 (10769.6－12407.4) | 11108.0 (10348.9－11922.8) | 11138.1 (10376.9－11955.0) | 11727.5 (10926.0－12587.7) |
| 2039 | 11839.9 (11022.9－12717.3) | 11349.9 (10566.8－12191.1) | 11380.7 (10595.4－12224.2) | 12015.3 (11186.3－12905.8) |
| 2040 | 12351.2 (11497.3－13268.5) | 11859.4 (11039.5－12740.2) | 11862.1 (11042.0－12743.1) | 12542.9 (11675.7－13474.4) |
| Female, all ages | |  |  |  |
| 2017 | 3476.4 (3453.0－3500.0) | 3476.2 (3452.7－3499.8) | 3476.3 (3452.8－3499.9) | 3476.3 (3452.8－3499.9) |
| 2018 | 3460.5 (3437.2－3484.0) | 3460.3 (3437.0－3483.8) | 3460.4 (3437.1－3483.9) | 3460.5 (3437.1－3484.0) |
| 2019 | 3496.0 (3470.9－3521.3) | 3495.8 (3470.7－3521.1) | 3495.9 (3470.9－3521.2) | 3496.0 (3470.9－3521.3) |
| 2020 | 3463.2 (3434.6－3491.9) | 3462.7 (3434.2－3491.5) | 3463.0 (3434.4－3491.7) | 3463.1 (3434.6－3491.9) |
| 2021 | 3454.9 (3423.3－3486.7) | 3454.3 (3422.7－3486.2) | 3454.6 (3423.1－3486.5) | 3454.8 (3423.3－3486.7) |
| 2022 | 3555.9 (3523.0－3589.1) | 3555.4 (3522.5－3588.6) | 3555.7 (3522.8－3588.8) | 3555.9 (3523.0－3589.1) |
| 2023 | 3589.1 (3555.6－3622.9) | 3588.6 (3555.1－3622.3) | 3588.7 (3555.2－3622.5) | 3588.9 (3555.5－3622.7) |
| 2024 | 3567.3 (3530.4－3604.7) | 3566.9 (3530.0－3604.3) | 3567.0 (3530.0－3604.4) | 3567.3 (3530.3－3604.7) |
| 2025 | 3611.6 (3572.4－3651.2) | 3611.3 (3572.2－3650.9) | 3611.2 (3572.1－3650.8) | 3611.5 (3572.4－3651.1) |
| 2026 | 3607.2 (3567.0－3647.8) | 3606.8 (3566.6－3647.4) | 3606.7 (3566.5－3647.3) | 3607.0 (3566.9－3647.6) |
| 2027 | 3575.6 (3532.5－3619.1) | 3575.2 (3532.2－3618.8) | 3575.1 (3532.1－3618.7) | 3575.5 (3532.5－3619.1) |
| 2028 | 3664.2 (3620.1－3708.9) | 3663.9 (3619.8－3708.6) | 3663.8 (3619.7－3708.4) | 3664.2 (3620.1－3708.8) |
| 2029 | 3740.6 (3694.5－3787.4) | 3740.3 (3694.2－3787.1) | 3740.0 (3693.9－3786.7) | 3740.5 (3694.4－3787.2) |
| 2030 | 3727.5 (3680.5－3775.1) | 3727.3 (3680.3－3774.9) | 3726.9 (3680.0－3774.5) | 3727.5 (3680.5－3775.1) |
| 2031 | 3770.1 (3720.9－3819.9) | 3769.9 (3720.8－3819.8) | 3769.4 (3720.3－3819.2) | 3770.0 (3720.8－3819.8) |
| 2032 | 3800.5 (3750.6－3851.1) | 3800.4 (3750.4－3851.0) | 3799.8 (3749.9－3850.4) | 3800.3 (3750.4－3850.9) |
| 2033 | 3760.6 (3708.6－3813.3) | 3760.4 (3708.4－3813.1) | 3759.8 (3707.8－3812.5) | 3760.4 (3708.5－3813.1) |
| 2034 | 3822.4 (3769.2－3876.4) | 3822.2 (3769.1－3876.2) | 3821.6 (3768.5－3875.5) | 3822.3 (3769.1－3876.2) |
| 2035 | 3933.6 (3877.4－3990.5) | 3933.4 (3877.3－3990.3) | 3932.7 (3876.5－3989.6) | 3933.4 (3877.3－3990.3) |
| 2036 | 3943.5 (3887.2－4000.6) | 3943.4 (3887.1－4000.5) | 3942.6 (3886.3－3999.7) | 3943.4 (3887.1－4000.5) |
| 2037 | 3977.9 (3919.7－4036.9) | 3977.9 (3919.7－4037.0) | 3977.0 (3918.8－4036.0) | 3977.8 (3919.6－4036.8) |
| 2038 | 4041.0 (3981.7－4101.1) | 4041.0 (3981.7－4101.1) | 4039.9 (3980.7－4100.1) | 4040.8 (3981.5－4100.9) |
| 2039 | 4011.1 (3950.7－4072.4) | 4011.0 (3950.7－4072.4) | 4010.0 (3949.6－4071.3) | 4010.9 (3950.5－4072.2) |
| 2040 | 4038.2 (3976.1－4101.2) | 4038.1 (3976.0－4101.2) | 4037.0 (3975.0－4100.1) | 4038.0 (3975.9－4101.1) |
| Total population, 20-49y | |  |  |  |
| 2017 | 780.0 (765.2－795.0) | 773.8 (759.1－788.7) | 776.3 (761.6－791.3) | 777.3 (762.7－792.3) |
| 2018 | 787.6 (772.7－802.8) | 780.0 (765.2－795.0) | 784.0 (769.2－799.2) | 785.8 (771.0－801.0) |
| 2019 | 790.1 (775.2－805.4) | 781.3 (766.5－796.4) | 786.3 (771.4－801.5) | 788.6 (773.6－803.8) |
| 2020 | 765.7 (750.8－781.0) | 752.7 (738.0－767.7) | 760.0 (745.1－775.1) | 763.4 (748.5－778.6) |
| 2021 | 756.6 (740.7－772.8) | 740.2 (724.7－756.1) | 750.0 (734.3－766.1) | 754.6 (738.8－770.8) |
| 2022 | 744.1 (727.9－760.6) | 729.2 (713.3－745.4) | 737.7 (721.7－754.1) | 743.0 (726.9－759.5) |
| 2023 | 714.3 (697.8－731.2) | 696.9 (680.8－713.4) | 705.6 (689.3－722.3) | 711.9 (695.5－728.8) |
| 2024 | 702.0 (685.7－718.7) | 684.1 (668.3－700.4) | 692.7 (676.6－709.2) | 699.9 (683.7－716.5) |
| 2025 | 694.5 (677.0－712.5) | 677.8 (660.7－695.4) | 685.0 (667.7－702.7) | 692.6 (675.1－710.5) |
| 2026 | 674.6 (657.2－692.5) | 657.1 (640.1－674.5) | 663.6 (646.5－681.2) | 671.8 (654.4－689.6) |
| 2027 | 670.8 (653.1－689.0) | 653.2 (636.0－670.9) | 659.0 (641.7－676.9) | 667.8 (650.2－685.9) |
| 2028 | 670.4 (652.7－688.5) | 654.7 (637.4－672.5) | 658.8 (641.4－676.6) | 667.7 (650.1－685.8) |
| 2029 | 655.8 (638.5－673.6) | 639.5 (622.7－656.9) | 642.8 (625.8－660.3) | 652.2 (635.0－669.9) |
| 2030 | 650.3 (631.9－669.2) | 634.0 (616.1－652.5) | 636.8 (618.7－655.3) | 646.7 (628.4－665.6) |
| 2031 | 645.3 (627.1－664.1) | 630.2 (612.4－648.6) | 631.7 (613.8－650.1) | 642.0 (623.8－660.7) |
| 2032 | 624.9 (607.2－643.1) | 609.0 (591.8－626.8) | 610.4 (593.1－628.2) | 621.0 (603.4－639.1) |
| 2033 | 611.7 (594.0－629.8) | 595.0 (577.9－612.7) | 596.5 (579.2－614.2) | 607.7 (590.1－625.7) |
| 2034 | 600.4 (583.0－618.4) | 584.7 (567.7－602.2) | 585.4 (568.4－603.0) | 596.9 (579.5－614.8) |
| 2035 | 578.6 (561.4－596.2) | 562.2 (545.5－579.4) | 562.9 (546.2－580.1) | 574.6 (557.6－592.2) |
| 2036 | 564.9 (548.2－582.2) | 548.2 (531.9－564.9) | 548.8 (532.5－565.6) | 560.9 (544.3－578.1) |
| 2037 | 556.3 (539.6－573.5) | 540.5 (524.2－557.2) | 540.3 (524.1－557.0) | 552.5 (536.0－569.6) |
| 2038 | 539.8 (523.4－556.7) | 524.0 (508.1－540.3) | 523.4 (507.5－539.7) | 535.7 (519.5－552.5) |
| 2039 | 530.2 (514.0－547.0) | 514.2 (498.5－530.5) | 513.3 (497.6－529.5) | 525.9 (509.8－542.6) |
| 2040 | 524.6 (508.5－541.1) | 509.6 (494.0－525.7) | 507.7 (492.2－523.8) | 520.4 (504.5－536.9) |
| Total population, 50-69y | |  |  |  |
| 2017 | 3961.5 (3946.7－3976.4) | 3974.0 (3959.2－3989.0) | 3955.8 (3941.0－3970.6) | 3949.3 (3934.6－3964.2) |
| 2018 | 3865.4 (3850.9－3879.9) | 3894.0 (3879.4－3908.6) | 3870.5 (3856.0－3885.0) | 3862.6 (3848.1－3877.1) |
| 2019 | 3768.6 (3734.9－3802.6) | 3794.2 (3760.2－3828.4) | 3777.0 (3743.2－3811.0) | 3772.0 (3738.3－3806.0) |
| 2020 | 3673.0 (3635.8－3710.6) | 3678.4 (3641.1－3716.1) | 3668.2 (3631.0－3705.7) | 3666.5 (3629.4－3704.0) |
| 2021 | 3633.4 (3596.1－3671.0) | 3610.6 (3573.6－3648.1) | 3621.2 (3584.0－3658.7) | 3628.1 (3590.9－3665.7) |
| 2022 | 3598.8 (3561.7－3636.4) | 3570.7 (3533.9－3608.0) | 3578.7 (3541.7－3616.0) | 3590.7 (3553.6－3628.2) |
| 2023 | 3487.2 (3449.8－3525.0) | 3460.8 (3423.7－3498.4) | 3453.5 (3416.5－3491.0) | 3465.5 (3428.3－3503.1) |
| 2024 | 3392.4 (3356.0－3429.2) | 3357.7 (3321.6－3394.2) | 3354.7 (3318.7－3391.2) | 3373.5 (3337.3－3410.1) |
| 2025 | 3357.4 (3319.8－3395.4) | 3309.2 (3272.2－3346.7) | 3314.1 (3277.0－3351.6) | 3341.0 (3303.6－3378.8) |
| 2026 | 3277.1 (3239.6－3315.0) | 3210.2 (3173.4－3247.3) | 3221.7 (3184.8－3259.0) | 3255.7 (3218.5－3293.4) |
| 2027 | 3229.4 (3190.8－3268.5) | 3142.1 (3104.5－3180.2) | 3169.0 (3131.1－3207.3) | 3213.4 (3175.0－3252.3) |
| 2028 | 3220.0 (3180.1－3260.4) | 3130.4 (3091.6－3169.7) | 3155.7 (3116.6－3195.3) | 3204.5 (3164.8－3244.7) |
| 2029 | 3097.0 (3054.3－3140.3) | 3013.7 (2972.2－3055.8) | 3025.6 (2983.9－3067.9) | 3072.8 (3030.4－3115.7) |
| 2030 | 3006.0 (2964.5－3048.0) | 2924.1 (2883.8－2965.0) | 2933.0 (2892.5－2974.0) | 2982.3 (2941.2－3024.0) |
| 2031 | 2979.7 (2937.6－3022.3) | 2896.9 (2856.0－2938.3) | 2902.1 (2861.1－2943.6) | 2954.2 (2912.5－2996.5) |
| 2032 | 2876.1 (2835.5－2917.2) | 2789.2 (2749.8－2829.1) | 2791.5 (2752.1－2831.4) | 2844.9 (2804.8－2885.7) |
| 2033 | 2825.4 (2785.5－2865.9) | 2739.6 (2700.9－2778.8) | 2741.2 (2702.5－2780.4) | 2797.7 (2758.2－2837.7) |
| 2034 | 2794.7 (2755.2－2834.7) | 2720.7 (2682.3－2759.7) | 2711.8 (2673.6－2750.7) | 2768.0 (2728.9－2807.6) |
| 2035 | 2661.7 (2624.1－2699.9) | 2603.9 (2567.1－2641.3) | 2579.9 (2543.4－2616.9) | 2631.5 (2594.3－2669.2) |
| 2036 | 2583.1 (2545.9－2620.9) | 2535.8 (2499.3－2572.9) | 2504.1 (2468.1－2540.8) | 2554.9 (2518.1－2592.3) |
| 2037 | 2542.5 (2503.8－2581.9) | 2492.0 (2454.0－2530.6) | 2459.9 (2422.4－2497.9) | 2513.1 (2474.8－2552.0) |
| 2038 | 2456.1 (2418.3－2494.6) | 2398.9 (2361.9－2436.4) | 2367.4 (2330.9－2404.4) | 2421.9 (2384.6－2459.8) |
| 2039 | 2416.7 (2379.4－2454.6) | 2355.5 (2319.1－2392.4) | 2326.0 (2290.1－2362.4) | 2383.5 (2346.8－2420.9) |
| 2040 | 2379.6 (2342.9－2417.0) | 2315.9 (2280.2－2352.2) | 2286.5 (2251.2－2322.3) | 2346.2 (2310.0－2383.0) |
| Total population, ≥70y | |  |  |  |
| 2017 | 13236.6 (12897.7－13584.3) | 13205.1 (12867.1－13552.0) | 13224.4 (12885.9－13571.9) | 13234.3 (12895.5－13582.0) |
| 2018 | 13094.7 (12759.4－13438.7) | 13029.7 (12696.1－13372.0) | 13066.9 (12732.4－13410.2) | 13086.6 (12751.6－13430.4) |
| 2019 | 12920.9 (12580.3－13270.7) | 12847.7 (12509.1－13195.5) | 12901.6 (12561.5－13250.9) | 12930.8 (12590.0－13280.9) |
| 2020 | 12736.7 (12392.7－13090.3) | 12660.0 (12318.1－13011.5) | 12729.3 (12385.5－13082.7) | 12767.9 (12423.1－13122.4) |
| 2021 | 12561.1 (12214.8－12917.2) | 12467.5 (12123.8－12821.0) | 12551.0 (12205.0－12906.9) | 12598.9 (12251.5－12956.1) |
| 2022 | 12379.4 (12032.1－12736.7) | 12298.0 (11953.0－12653.0) | 12367.5 (12020.5－12724.5) | 12424.3 (12075.8－12783.0) |
| 2023 | 12200.8 (11853.3－12558.4) | 12123.4 (11778.2－12478.8) | 12179.5 (11832.7－12536.5) | 12245.1 (11896.4－12604.0) |
| 2024 | 12025.7 (11678.9－12382.8) | 11944.4 (11599.9－12299.1) | 11987.8 (11642.1－12343.8) | 12061.9 (11714.0－12420.1) |
| 2025 | 11839.0 (11493.7－12194.5) | 11761.6 (11418.7－12114.9) | 11793.1 (11449.2－12147.3) | 11875.4 (11529.1－12232.1) |
| 2026 | 11646.4 (11303.5－11999.6) | 11575.7 (11235.0－11926.8) | 11595.9 (11254.5－11947.6) | 11686.2 (11342.1－12040.6) |
| 2027 | 11450.8 (11110.9－11801.1) | 11387.4 (11049.4－11735.7) | 11396.8 (11058.5－11745.4) | 11494.8 (11153.6－11846.4) |
| 2028 | 11248.7 (10912.5－11595.3) | 11197.0 (10862.3－11542.1) | 11196.3 (10861.7－11541.3) | 11301.7 (10963.9－11650.0) |
| 2029 | 11048.5 (10716.2－11391.1) | 11005.2 (10674.2－11346.5) | 10995.1 (10664.3－11336.0) | 11107.5 (10773.4－11452.0) |
| 2030 | 10851.8 (10523.6－11190.2) | 10812.4 (10485.4－11149.6) | 10793.3 (10466.9－11129.9) | 10912.6 (10582.5－11252.9) |
| 2031 | 10654.9 (10331.1－10988.8) | 10619.1 (10296.4－10951.9) | 10591.6 (10269.7－10923.5) | 10717.3 (10391.7－11053.1) |
| 2032 | 10460.0 (10140.9－10789.2) | 10425.5 (10107.4－10753.6) | 10390.2 (10073.2－10717.2) | 10522.1 (10201.0－10853.2) |
| 2033 | 10265.5 (9951.1－10589.8) | 10232.2 (9918.9－10555.4) | 10189.4 (9877.4－10511.3) | 10327.2 (10011.0－10653.5) |
| 2034 | 10068.8 (9759.5－10387.9) | 10039.3 (9730.9－10357.5) | 9989.7 (9682.8－10306.3) | 10133.1 (9821.8－10454.2) |
| 2035 | 9872.7 (9568.6－10186.5) | 9847.2 (9543.9－10160.2) | 9791.2 (9489.6－10102.4) | 9939.9 (9633.7－10255.8) |
| 2036 | 9677.6 (9378.8－9986.0) | 9656.2 (9358.0－9963.9) | 9594.2 (9297.9－9899.8) | 9747.9 (9446.9－10058.4) |
| 2037 | 9483.8 (9190.3－9786.6) | 9466.5 (9173.6－9768.8) | 9398.9 (9108.0－9699.0) | 9557.3 (9261.5－9862.5) |
| 2038 | 9293.1 (9005.0－9590.4) | 9278.3 (8990.6－9575.1) | 9205.5 (8920.1－9500.0) | 9368.3 (9077.9－9668.0) |
| 2039 | 9105.1 (8822.4－9397.0) | 9091.8 (8809.4－9383.1) | 9014.1 (8734.2－9303.0) | 9181.2 (8896.1－9475.4) |
| 2040 | 8919.1 (8641.7－9205.4) | 8907.1 (8630.1－9193.0) | 8825.0 (8550.6－9108.3) | 8996.0 (8716.2－9284.7) |
| Total population, all ages | |  |  |  |
| 2017 | 3998.1 (3939.0－4058.0) | 3972.6 (3913.9－4032.2) | 3990.5 (3931.5－4050.3) | 3997.9 (3938.8－4057.9) |
| 2018 | 4027.5 (3968.1－4087.8) | 3980.0 (3921.3－4039.6) | 4013.0 (3953.8－4073.1) | 4027.1 (3967.7－4087.5) |
| 2019 | 3939.7 (3861.4－4019.6) | 3861.0 (3784.3－3939.4) | 3915.3 (3837.4－3994.7) | 3939.1 (3860.7－4019.0) |
| 2020 | 3888.1 (3807.6－3970.3) | 3782.1 (3703.8－3862.1) | 3854.6 (3774.8－3936.1) | 3887.2 (3806.7－3969.4) |
| 2021 | 3935.9 (3852.5－4021.1) | 3825.1 (3744.0－3907.9) | 3900.0 (3817.3－3984.4) | 3934.9 (3851.5－4020.1) |
| 2022 | 4011.1 (3917.2－4107.4) | 3888.0 (3796.9－3981.2) | 3970.2 (3877.1－4065.4) | 4009.9 (3916.0－4106.1) |
| 2023 | 4033.0 (3935.8－4132.5) | 3906.7 (3812.7－4003.1) | 3984.4 (3888.4－4082.7) | 4031.5 (3934.5－4131.0) |
| 2024 | 3982.3 (3886.4－4080.6) | 3842.6 (3750.0－3937.4) | 3923.3 (3828.8－4020.1) | 3980.6 (3884.7－4078.9) |
| 2025 | 3934.1 (3834.1－4036.7) | 3788.7 (3692.4－3887.5) | 3867.2 (3768.9－3968.0) | 3932.2 (3832.2－4034.7) |
| 2026 | 3946.1 (3844.8－4050.1) | 3804.0 (3706.3－3904.2) | 3873.8 (3774.4－3975.9) | 3944.0 (3842.8－4047.9) |
| 2027 | 4006.4 (3902.8－4112.7) | 3871.4 (3771.4－3974.2) | 3929.5 (3827.9－4033.8) | 4004.1 (3900.6－4110.4) |
| 2028 | 4048.7 (3940.9－4159.5) | 3913.3 (3809.1－4020.3) | 3965.0 (3859.4－4073.5) | 4046.3 (3938.5－4157.0) |
| 2029 | 4037.3 (3929.7－4147.9) | 3893.8 (3790.0－4000.5) | 3945.1 (3839.9－4053.2) | 4034.6 (3927.1－4145.2) |
| 2030 | 4002.5 (3894.5－4113.4) | 3851.0 (3747.2－3957.8) | 3902.2 (3796.9－4010.4) | 3999.6 (3891.7－4110.4) |
| 2031 | 3999.1 (3889.7－4111.6) | 3847.1 (3741.8－3955.3) | 3892.7 (3786.2－4002.2) | 3996.0 (3886.7－4108.4) |
| 2032 | 4040.3 (3929.8－4153.9) | 3892.3 (3785.8－4001.8) | 3928.5 (3821.0－4039.0) | 4037.0 (3926.6－4150.6) |
| 2033 | 4089.1 (3975.9－4205.5) | 3942.8 (3833.6－4055.0) | 3971.0 (3861.1－4084.0) | 4085.7 (3972.6－4201.9) |
| 2034 | 4104.7 (3990.5－4222.2) | 3953.6 (3843.6－4066.7) | 3978.9 (3868.2－4092.8) | 4101.1 (3987.0－4218.4) |
| 2035 | 4090.3 (3976.3－4207.6) | 3932.0 (3822.4－4044.7) | 3956.8 (3846.5－4070.2) | 4086.5 (3972.6－4203.6) |
| 2036 | 4083.9 (3969.2－4202.0) | 3922.1 (3811.9－4035.5) | 3943.7 (3832.9－4057.7) | 4079.9 (3965.3－4197.9) |
| 2037 | 4110.9 (3995.3－4229.9) | 3950.4 (3839.3－4064.7) | 3964.7 (3853.2－4079.4) | 4106.7 (3991.2－4225.6) |
| 2038 | 4157.0 (4039.7－4277.7) | 3998.1 (3885.3－4114.2) | 4004.4 (3891.4－4120.7) | 4152.7 (4035.5－4273.3) |
| 2039 | 4189.0 (4070.2－4311.2) | 4027.6 (3913.4－4145.1) | 4029.1 (3914.8－4146.6) | 4184.5 (4065.8－4306.6) |
| 2040 | 4194.3 (4075.4－4316.7) | 4027.1 (3912.9－4144.6) | 4026.8 (3912.7－4144.4) | 4189.6 (4070.8－4311.9) |

DALY, disability-adjusted life year; y: year

**Supplementary Table 3: Predicted DALY rates (95% prediction intervals) for cancer by age groups, sex (male, female), and total population, 2017–2040.**

| Year | Reference | Better scenario | Moderate scenario | Worse scenario |
| --- | --- | --- | --- | --- |
| Male, 20-49y | |  |  |  |
| 2017 | 940.9 (910.5－972.4) | 931.7 (901.6－962.9) | 934.4 (904.1－965.6) | 935.5 (905.2－966.8) |
| 2018 | 928.0 (898.0－959.1) | 913.6 (884.0－944.2) | 919.7 (889.9－950.5) | 922.4 (892.5－953.2) |
| 2019 | 909.5 (877.4－942.7) | 892.4 (860.9－925.0) | 901.9 (870.1－934.9) | 906.3 (874.3－939.4) |
| 2020 | 894.0 (862.0－927.2) | 870.1 (838.9－902.4) | 882.9 (851.3－915.7) | 888.9 (857.1－921.9) |
| 2021 | 876.9 (845.4－909.6) | 847.6 (817.2－879.2) | 863.4 (832.4－895.6) | 871.0 (839.8－903.5) |
| 2022 | 856.5 (825.8－888.4) | 829.9 (800.1－860.8) | 843.8 (813.5－875.2) | 852.9 (822.3－884.7) |
| 2023 | 840.8 (810.6－872.1) | 812.1 (783.0－842.4) | 824.2 (794.6－854.9) | 834.8 (804.8－865.9) |
| 2024 | 824.0 (794.4－854.7) | 794.4 (765.8－824.0) | 804.8 (775.8－834.7) | 816.6 (787.3－847.0) |
| 2025 | 804.5 (775.6－834.5) | 776.7 (748.8－805.6) | 785.4 (757.2－814.7) | 798.5 (769.9－828.3) |
| 2026 | 787.8 (759.5－817.1) | 759.1 (731.8－787.3) | 766.3 (738.8－794.9) | 780.5 (752.5－809.6) |
| 2027 | 771.1 (743.4－799.9) | 741.6 (714.9－769.2) | 747.4 (720.5－775.2) | 762.7 (735.3－791.1) |
| 2028 | 751.8 (724.8－779.8) | 724.2 (698.2－751.2) | 728.7 (702.5－755.8) | 744.9 (718.1－772.7) |
| 2029 | 734.9 (708.5－762.3) | 706.9 (681.5－733.3) | 710.2 (684.6－736.6) | 727.3 (701.2－754.4) |
| 2030 | 718.5 (692.7－745.3) | 689.9 (665.1－715.6) | 691.9 (667.0－717.7) | 709.8 (684.3－736.3) |
| 2031 | 700.2 (675.1－726.3) | 672.9 (648.8－698.0) | 673.9 (649.7－699.0) | 692.5 (667.7－718.3) |
| 2032 | 683.5 (659.0－709.0) | 656.2 (632.7－680.7) | 656.1 (632.5－680.5) | 675.4 (651.2－700.6) |
| 2033 | 667.8 (643.9－692.7) | 639.7 (616.7－663.5) | 638.6 (615.6－662.4) | 658.5 (634.9－683.1) |
| 2034 | 650.4 (627.0－674.6) | 623.4 (601.0－646.6) | 621.4 (599.0－644.5) | 641.8 (618.8－665.7) |
| 2035 | 634.0 (611.3－657.7) | 607.3 (585.5－629.9) | 604.4 (582.7－626.9) | 625.4 (602.9－648.7) |
| 2036 | 618.7 (596.5－641.8) | 591.4 (570.2－613.4) | 587.8 (566.6－609.6) | 609.1 (587.2－631.8) |
| 2037 | 602.1 (580.5－624.6) | 575.8 (555.1－597.2) | 571.4 (550.9－592.7) | 593.1 (571.8－615.2) |
| 2038 | 586.3 (565.2－608.1) | 560.4 (540.3－581.3) | 555.3 (535.4－576.0) | 577.4 (556.6－598.9) |
| 2039 | 571.6 (551.1－592.9) | 545.3 (525.7－565.6) | 539.6 (520.2－559.7) | 561.9 (541.7－582.8) |
| 2040 | 556.0 (536.1－576.7) | 530.5 (511.4－550.2) | 524.2 (505.4－543.7) | 546.7 (527.1－567.1) |
| Male, 50-69y | |  |  |  |
| 2017 | 9652.2 (9506.5－9800.1) | 9556.0 (9411.8－9702.5) | 9619.4 (9474.2－9766.8) | 9643.7 (9498.2－9791.5) |
| 2018 | 9466.8 (9324.1－9611.6) | 9324.3 (9183.7－9466.9) | 9416.4 (9274.5－9560.5) | 9452.8 (9310.3－9597.4) |
| 2019 | 9497.0 (9315.8－9681.7) | 9268.7 (9091.9－9449.0) | 9416.0 (9236.3－9599.1) | 9474.9 (9294.1－9659.2) |
| 2020 | 9340.3 (9162.0－9522.2) | 9098.6 (8924.9－9275.8) | 9251.5 (9074.9－9431.6) | 9314.6 (9136.7－9495.9) |
| 2021 | 9088.5 (8890.6－9290.7) | 8792.0 (8600.6－8987.6) | 8978.6 (8783.1－9178.4) | 9056.7 (8859.6－9258.2) |
| 2022 | 8795.9 (8590.0－9006.7) | 8495.9 (8297.0－8699.5) | 8670.3 (8467.3－8878.1) | 8759.3 (8554.3－8969.2) |
| 2023 | 8597.0 (8390.5－8808.6) | 8295.1 (8095.8－8499.2) | 8456.9 (8253.8－8665.0) | 8555.8 (8350.3－8766.3) |
| 2024 | 8443.6 (8240.0－8652.2) | 8156.9 (7960.2－8358.4) | 8295.0 (8095.0－8499.9) | 8398.8 (8196.3－8606.3) |
| 2025 | 8219.1 (8020.9－8422.3) | 7954.2 (7762.4－8150.9) | 8065.3 (7870.8－8264.7) | 8171.7 (7974.6－8373.7) |
| 2026 | 8041.0 (7842.6－8244.5) | 7766.6 (7574.9－7963.1) | 7872.4 (7678.1－8071.6) | 7989.1 (7791.9－8191.2) |
| 2027 | 7918.1 (7715.4－8126.0) | 7646.3 (7450.6－7847.1) | 7738.6 (7540.5－7941.8) | 7862.3 (7661.1－8068.8) |
| 2028 | 7870.2 (7667.1－8078.6) | 7590.4 (7394.5－7791.4) | 7675.9 (7477.9－7879.2) | 7809.6 (7608.2－8016.5) |
| 2029 | 7742.6 (7542.6－7948.0) | 7471.4 (7278.4－7669.6) | 7540.4 (7345.6－7740.4) | 7678.9 (7480.5－7882.6) |
| 2030 | 7572.4 (7376.4－7773.5) | 7286.4 (7097.8－7479.9) | 7356.0 (7165.6－7551.4) | 7504.5 (7310.3－7703.8) |
| 2031 | 7392.1 (7199.1－7590.2) | 7098.5 (6913.2－7288.8) | 7164.6 (6977.6－7356.7) | 7320.7 (7129.6－7517.0) |
| 2032 | 7227.2 (7034.2－7425.4) | 6930.3 (6745.2－7120.4) | 6990.3 (6803.7－7182.1) | 7152.7 (6961.7－7348.9) |
| 2033 | 7026.7 (6837.6－7221.1) | 6738.7 (6557.3－6925.1) | 6785.8 (6603.1－6973.5) | 6950.3 (6763.2－7142.6) |
| 2034 | 6783.4 (6600.4－6971.5) | 6503.5 (6328.1－6683.8) | 6539.8 (6363.4－6721.1) | 6705.7 (6524.8－6891.6) |
| 2035 | 6570.6 (6392.7－6753.4) | 6292.6 (6122.2－6467.6) | 6322.4 (6151.2－6498.3) | 6491.1 (6315.4－6671.7) |
| 2036 | 6403.8 (6230.3－6582.2) | 6134.2 (5968.0－6305.1) | 6152.7 (5986.0－6324.1) | 6322.9 (6151.5－6499.0) |
| 2037 | 6270.1 (6100.1－6444.9) | 6007.6 (5844.7－6175.1) | 6015.3 (5852.2－6182.9) | 6187.4 (6019.6－6359.8) |
| 2038 | 6121.2 (5954.5－6292.5) | 5866.4 (5706.6－6030.7) | 5863.7 (5704.0－6027.9) | 6037.0 (5872.6－6206.0) |
| 2039 | 5984.7 (5820.9－6153.0) | 5726.1 (5569.4－5887.2) | 5721.1 (5564.6－5882.1) | 5898.4 (5737.0－6064.3) |
| 2040 | 5864.8 (5703.6－6030.6) | 5604.0 (5450.0－5762.5) | 5595.7 (5441.8－5753.8) | 5776.5 (5617.7－5939.8) |
| Male, ≥70 | |  |  |  |
| 2017 | 20501.2 (19989.1－21026.4) | 20474.4 (19962.9－20998.9) | 20458.8 (19947.8－20983.0) | 20450.3 (19939.5－20974.2) |
| 2018 | 20333.4 (19825.5－20854.3) | 20292.3 (19785.4－20812.1) | 20272.3 (19765.9－20791.6) | 20260.0 (19754.0－20779.1) |
| 2019 | 20205.8 (19648.4－20779.0) | 20140.8 (19585.2－20712.2) | 20112.1 (19557.3－20682.7) | 20093.5 (19539.2－20663.5) |
| 2020 | 20067.1 (19499.1－20651.6) | 19975.6 (19410.2－20557.4) | 19943.5 (19379.0－20524.4) | 19920.6 (19356.7－20500.8) |
| 2021 | 19894.2 (19325.7－20479.4) | 19827.9 (19261.3－20411.1) | 19790.8 (19225.3－20373.0) | 19762.4 (19197.7－20343.7) |
| 2022 | 19742.9 (19176.3－20326.3) | 19636.4 (19072.8－20216.6) | 19635.8 (19072.3－20216.1) | 19603.1 (19040.5－20182.3) |
| 2023 | 19591.2 (19027.6－20171.5) | 19458.6 (18898.8－20035.0) | 19492.2 (18931.5－20069.6) | 19454.5 (18894.8－20030.7) |
| 2024 | 19464.9 (18904.2－20042.2) | 19280.9 (18725.4－19852.8) | 19349.2 (18791.8－19923.1) | 19307.2 (18751.0－19879.9) |
| 2025 | 19344.8 (18787.1－19919.1) | 19113.4 (18562.4－19680.8) | 19214.9 (18661.0－19785.3) | 19168.3 (18615.7－19737.4) |
| 2026 | 19248.1 (18692.9－19819.8) | 18948.1 (18401.5－19510.8) | 19082.9 (18532.4－19649.6) | 19032.2 (18483.2－19597.4) |
| 2027 | 19146.4 (18594.0－19715.2) | 18790.9 (18248.7－19349.2) | 18957.8 (18410.9－19521.1) | 18902.9 (18357.5－19464.5) |
| 2028 | 19021.3 (18472.4－19586.5) | 18636.9 (18099.1－19190.7) | 18835.9 (18292.3－19395.6) | 18777.0 (18235.1－19334.9) |
| 2029 | 18920.1 (18374.0－19482.4) | 18489.7 (17956.1－19039.2) | 18719.9 (18179.6－19276.2) | 18657.0 (18118.5－19211.4) |
| 2030 | 18797.2 (18254.7－19355.9) | 18346.2 (17816.7－18891.5) | 18607.4 (18070.3－19160.4) | 18540.7 (18005.5－19091.7) |
| 2031 | 18695.0 (18155.4－19250.7) | 18208.6 (17683.0－18749.8) | 18500.1 (17966.1－19049.9) | 18429.5 (17897.6－18977.3) |
| 2032 | 18607.7 (18070.6－19160.8) | 18074.8 (17553.1－18612.1) | 18396.4 (17865.4－18943.2) | 18322.2 (17793.4－18866.8) |
| 2033 | 18530.5 (17995.6－19081.3) | 17946.3 (17428.3－18479.7) | 18297.3 (17769.2－18841.2) | 18219.5 (17693.6－18761.1) |
| 2034 | 18455.6 (17922.9－19004.2) | 17821.6 (17307.2－18351.3) | 18201.9 (17676.5－18742.9) | 18120.6 (17597.6－18659.2) |
| 2035 | 18376.5 (17846.0－18922.7) | 17701.6 (17190.6－18227.8) | 18110.7 (17587.9－18649.0) | 18025.9 (17505.6－18561.7) |
| 2036 | 18304.9 (17776.6－18849.0) | 17585.4 (17077.8－18108.1) | 18023.0 (17502.7－18558.7) | 17934.8 (17417.1－18467.9) |
| 2037 | 18213.4 (17687.6－18754.7) | 17473.4 (16969.0－17992.7) | 17939.1 (17421.2－18472.3) | 17847.6 (17332.4－18378.1) |
| 2038 | 18140.8 (17617.2－18680.0) | 17365.0 (16863.8－17881.1) | 17858.5 (17343.1－18389.4) | 17763.8 (17251.1－18291.8) |
| 2039 | 18075.2 (17553.4－18612.4) | 17260.4 (16762.2－17773.5) | 17781.5 (17268.3－18310.1) | 17683.6 (17173.1－18209.2) |
| 2040 | 18012.0 (17492.1－18547.4) | 17159.3 (16664.0－17669.4) | 17707.7 (17196.6－18234.0) | 17606.6 (17098.4－18129.9) |
| Male, all ages | |  |  |  |
| 2017 | 6651.8 (6527.8－6778.1) | 6549.5 (6427.4－6673.9) | 6617.3 (6493.9－6743.0) | 6644.1 (6520.3－6770.3) |
| 2018 | 6758.7 (6632.7－6887.0) | 6610.0 (6486.8－6735.5) | 6719.5 (6594.3－6847.1) | 6764.1 (6638.0－6892.5) |
| 2019 | 6802.4 (6674.7－6932.6) | 6613.1 (6488.9－6739.6) | 6764.5 (6637.5－6894.0) | 6827.7 (6699.5－6958.3) |
| 2020 | 6772.1 (6644.5－6902.1) | 6533.4 (6410.3－6658.9) | 6715.6 (6589.0－6844.5) | 6793.5 (6665.5－6924.0) |
| 2021 | 6709.1 (6574.9－6846.1) | 6432.2 (6303.5－6563.5) | 6629.0 (6496.3－6764.3) | 6715.8 (6581.5－6852.9) |
| 2022 | 6671.8 (6527.2－6819.6) | 6416.0 (6276.9－6558.1) | 6581.9 (6439.2－6727.7) | 6674.4 (6529.7－6822.2) |
| 2023 | 6728.8 (6581.0－6879.8) | 6452.6 (6310.9－6597.4) | 6605.1 (6460.1－6753.3) | 6710.2 (6562.9－6860.8) |
| 2024 | 6835.7 (6680.7－6994.3) | 6542.1 (6393.8－6693.8) | 6688.2 (6536.6－6843.3) | 6809.3 (6654.9－6967.2) |
| 2025 | 6936.3 (6771.9－7104.8) | 6631.4 (6474.2－6792.5) | 6779.8 (6619.0－6944.4) | 6920.5 (6756.4－7088.6) |
| 2026 | 6992.9 (6822.8－7167.1) | 6670.7 (6508.5－6837.0) | 6819.7 (6653.9－6989.6) | 6979.1 (6809.4－7153.0) |
| 2027 | 6980.4 (6810.7－7154.4) | 6654.0 (6492.2－6819.8) | 6792.7 (6627.6－6962.0) | 6965.4 (6796.0－7139.0) |
| 2028 | 6922.2 (6749.3－7099.5) | 6614.2 (6449.0－6783.6) | 6732.4 (6564.3－6904.9) | 6913.2 (6740.5－7090.2) |
| 2029 | 6901.6 (6723.5－7084.4) | 6594.3 (6424.2－6769.0) | 6691.0 (6518.3－6868.2) | 6879.4 (6701.9－7061.6) |
| 2030 | 6939.4 (6759.6－7124.0) | 6624.7 (6453.1－6801.0) | 6704.5 (6530.8－6882.9) | 6903.4 (6724.6－7087.1) |
| 2031 | 7019.8 (6837.0－7207.5) | 6695.9 (6521.5－6874.9) | 6768.2 (6592.0－6949.2) | 6982.8 (6800.9－7169.5) |
| 2032 | 7117.9 (6928.2－7312.9) | 6770.7 (6590.2－6956.1) | 6842.2 (6659.8－7029.6) | 7075.4 (6886.8－7269.2) |
| 2033 | 7179.0 (6984.9－7378.5) | 6812.2 (6628.0－7001.5) | 6882.1 (6696.0－7073.3) | 7132.7 (6939.9－7331.0) |
| 2034 | 7172.4 (6978.5－7371.7) | 6809.1 (6625.0－6998.3) | 6870.5 (6684.7－7061.4) | 7134.1 (6941.2－7332.4) |
| 2035 | 7141.6 (6946.5－7342.2) | 6782.3 (6597.0－6972.7) | 6827.7 (6641.2－7019.5) | 7100.1 (6906.2－7299.5) |
| 2036 | 7124.7 (6926.9－7328.0) | 6768.3 (6580.5－6961.5) | 6795.0 (6606.4－6988.9) | 7074.9 (6878.5－7276.9) |
| 2037 | 7144.3 (6945.2－7349.0) | 6791.4 (6602.2－6986.1) | 6802.5 (6613.0－6997.5) | 7092.8 (6895.2－7296.1) |
| 2038 | 7214.8 (7013.5－7422.0) | 6848.3 (6657.2－7044.9) | 6850.6 (6659.4－7047.2) | 7155.4 (6955.7－7360.9) |
| 2039 | 7301.9 (7095.8－7514.0) | 6912.4 (6717.3－7113.1) | 6911.1 (6716.1－7111.8) | 7233.5 (7029.3－7443.5) |
| 2040 | 7354.7 (7145.4－7570.2) | 6953.5 (6755.6－7157.2) | 6949.2 (6751.4－7152.7) | 7288.2 (7080.8－7501.7) |
| Female, 20-49y | |  |  |  |
| 2017 | 1116.4 (1086.9－1146.7) | 1118.5 (1089.0－1148.9) | 1116.7 (1087.2－1146.9) | 1116.9 (1087.4－1147.1) |
| 2018 | 1100.5 (1071.5－1130.3) | 1070.4 (1042.2－1099.4) | 1089.2 (1060.4－1118.7) | 1098.6 (1069.7－1128.4) |
| 2019 | 1084.8 (1045.4－1125.7) | 1026.5 (989.2－1065.2) | 1062.5 (1023.9－1102.6) | 1080.7 (1041.4－1121.4) |
| 2020 | 1067.7 (1028.9－1108.0) | 984.7 (948.9－1021.8) | 1035.2 (997.6－1074.3) | 1061.5 (1023.0－1101.5) |
| 2021 | 1050.0 (1011.9－1089.6) | 945.2 (910.9－980.8) | 1007.9 (971.3－1045.9) | 1041.7 (1003.9－1081.0) |
| 2022 | 1031.4 (994.0－1070.3) | 926.1 (892.5－961.0) | 980.4 (944.8－1017.3) | 1021.2 (984.1－1059.7) |
| 2023 | 1012.1 (975.4－1050.3) | 906.5 (873.5－940.6) | 952.8 (918.2－988.7) | 1000.0 (963.7－1037.7) |
| 2024 | 992.2 (956.1－1029.6) | 886.3 (854.1－919.7) | 925.1 (891.5－960.0) | 978.3 (942.8－1015.2) |
| 2025 | 971.6 (936.3－1008.2) | 865.7 (834.3－898.3) | 897.5 (864.9－931.3) | 956.0 (921.3－992.1) |
| 2026 | 950.5 (916.0－986.3) | 844.7 (814.0－876.6) | 869.9 (838.3－902.7) | 933.4 (899.4－968.5) |
| 2027 | 928.9 (895.2－963.9) | 823.4 (793.5－854.5) | 842.5 (811.9－874.2) | 910.3 (877.2－944.6) |
| 2028 | 906.9 (874.0－941.1) | 801.9 (772.8－832.1) | 815.2 (785.5－845.9) | 886.9 (854.7－920.3) |
| 2029 | 884.6 (852.5－917.9) | 780.1 (751.8－809.5) | 788.1 (759.5－817.8) | 863.3 (831.9－895.8) |
| 2030 | 862.0 (830.7－894.5) | 758.3 (730.7－786.8) | 761.3 (733.6－790.0) | 839.5 (809.0－871.2) |
| 2031 | 839.2 (808.7－870.8) | 736.3 (709.5－764.1) | 734.8 (708.1－762.5) | 815.6 (786.0－846.4) |
| 2032 | 816.2 (786.6－847.0) | 714.3 (688.4－741.2) | 708.6 (682.9－735.3) | 791.7 (762.9－821.5) |
| 2033 | 793.2 (764.4－823.1) | 692.4 (667.2－718.5) | 682.8 (658.0－708.6) | 767.8 (739.9－796.7) |
| 2034 | 770.2 (742.2－799.2) | 670.5 (646.2－695.8) | 657.5 (633.6－682.3) | 743.9 (716.9－772.0) |
| 2035 | 747.1 (720.0－775.3) | 648.9 (625.3－673.3) | 632.7 (609.7－656.5) | 720.2 (694.1－747.4) |
| 2036 | 724.2 (697.9－751.5) | 627.4 (604.6－651.0) | 608.3 (586.2－631.3) | 696.7 (671.4－723.0) |
| 2037 | 701.5 (676.0－727.9) | 606.1 (584.1－628.9) | 584.5 (563.3－606.5) | 673.5 (649.0－698.8) |
| 2038 | 679.0 (654.3－704.6) | 585.1 (563.9－607.2) | 561.3 (540.9－582.4) | 650.5 (626.9－675.0) |
| 2039 | 656.7 (632.8－681.4) | 564.5 (543.9－585.7) | 538.6 (519.0－558.9) | 627.8 (605.0－651.5) |
| 2040 | 634.7 (611.6－658.6) | 544.2 (524.4－564.7) | 516.5 (497.8－536.0) | 605.6 (583.6－628.4) |
| Female, 50-69y | |  |  |  |
| 2017 | 5398.5 (5351.8－5445.6) | 5388.6 (5342.0－5435.6) | 5386.8 (5340.2－5433.8) | 5386.3 (5339.7－5433.3) |
| 2018 | 5423.3 (5376.5－5470.6) | 5403.0 (5356.3－5450.1) | 5415.3 (5368.5－5462.5) | 5420.8 (5373.9－5468.0) |
| 2019 | 5602.5 (5529.8－5676.1) | 5560.0 (5487.9－5633.1) | 5592.6 (5520.1－5666.1) | 5606.8 (5534.0－5680.4) |
| 2020 | 5560.2 (5484.1－5637.2) | 5486.9 (5411.9－5563.0) | 5531.6 (5455.9－5608.3) | 5551.3 (5475.4－5628.2) |
| 2021 | 5556.3 (5463.7－5650.4) | 5466.2 (5375.2－5558.8) | 5527.5 (5435.5－5621.1) | 5554.8 (5462.3－5648.8) |
| 2022 | 5551.0 (5456.3－5647.4) | 5453.2 (5360.1－5547.9) | 5515.8 (5421.6－5611.6) | 5548.7 (5454.0－5645.0) |
| 2023 | 5440.3 (5338.3－5544.2) | 5341.5 (5241.4－5443.5) | 5391.2 (5290.1－5494.1) | 5423.6 (5322.0－5527.2) |
| 2024 | 5332.4 (5230.4－5436.5) | 5247.0 (5146.6－5349.4) | 5288.1 (5186.9－5391.3) | 5321.6 (5219.8－5425.4) |
| 2025 | 5373.8 (5263.6－5486.4) | 5293.8 (5185.2－5404.7) | 5329.6 (5220.3－5441.2) | 5365.9 (5255.9－5478.3) |
| 2026 | 5333.5 (5221.8－5447.5) | 5254.5 (5144.5－5366.8) | 5279.4 (5168.9－5392.3) | 5315.9 (5204.6－5429.5) |
| 2027 | 5342.8 (5230.3－5457.8) | 5269.6 (5158.7－5383.0) | 5293.2 (5181.8－5407.1) | 5333.9 (5221.6－5448.7) |
| 2028 | 5459.3 (5338.5－5582.8) | 5375.9 (5257.0－5497.5) | 5404.1 (5284.5－5526.4) | 5452.1 (5331.5－5575.5) |
| 2029 | 5468.3 (5347.2－5592.2) | 5372.5 (5253.5－5494.2) | 5398.1 (5278.6－5520.3) | 5449.7 (5329.0－5573.1) |
| 2030 | 5465.5 (5339.9－5594.0) | 5368.1 (5244.8－5494.3) | 5394.7 (5270.7－5521.6) | 5451.4 (5326.1－5579.6) |
| 2031 | 5524.6 (5397.6－5654.5) | 5418.4 (5293.9－5545.8) | 5447.2 (5322.0－5575.3) | 5510.0 (5383.4－5639.6) |
| 2032 | 5493.8 (5366.2－5624.4) | 5384.0 (5258.9－5512.0) | 5404.6 (5279.1－5533.1) | 5468.3 (5341.3－5598.3) |
| 2033 | 5444.5 (5316.0－5576.0) | 5345.7 (5219.6－5474.9) | 5360.5 (5234.1－5490.0) | 5425.9 (5297.9－5557.0) |
| 2034 | 5508.8 (5376.6－5644.3) | 5408.9 (5279.1－5542.0) | 5421.9 (5291.8－5555.2) | 5491.8 (5360.0－5626.9) |
| 2035 | 5508.0 (5375.1－5644.2) | 5406.6 (5276.1－5540.3) | 5411.6 (5281.0－5545.5) | 5482.7 (5350.4－5618.3) |
| 2036 | 5508.6 (5375.6－5644.9) | 5413.4 (5282.7－5547.3) | 5415.2 (5284.4－5549.1) | 5489.5 (5356.9－5625.3) |
| 2037 | 5612.0 (5474.2－5753.3) | 5506.9 (5371.7－5645.5) | 5511.3 (5376.0－5650.0) | 5592.5 (5455.2－5733.3) |
| 2038 | 5636.4 (5498.0－5778.2) | 5521.4 (5385.8－5660.3) | 5522.2 (5386.6－5661.2) | 5606.6 (5469.0－5747.8) |
| 2039 | 5639.3 (5498.5－5783.7) | 5526.9 (5389.0－5668.4) | 5525.9 (5388.0－5667.4) | 5614.2 (5474.0－5757.9) |
| 2040 | 5715.7 (5573.0－5862.1) | 5594.4 (5454.7－5737.7) | 5595.3 (5455.5－5738.5) | 5689.9 (5547.8－5835.6) |
| Female, ≥70y | |  |  |  |
| 2017 | 9244.9 (9195.8－9294.2) | 9222.3 (9173.4－9271.5) | 9234.3 (9185.3－9283.5) | 9239.9 (9190.9－9289.2) |
| 2018 | 9188.1 (9139.3－9237.1) | 9136.3 (9087.8－9185.0) | 9168.9 (9120.2－9217.8) | 9184.2 (9135.5－9233.2) |
| 2019 | 9000.7 (8937.9－9063.9) | 8967.5 (8905.0－9030.5) | 8992.1 (8929.4－9055.2) | 9004.2 (8941.4－9067.4) |
| 2020 | 8816.7 (8695.1－8940.0) | 8798.1 (8676.8－8921.1) | 8814.9 (8693.3－8938.1) | 8823.9 (8702.2－8947.3) |
| 2021 | 8701.3 (8551.1－8854.2) | 8666.0 (8516.3－8818.3) | 8694.5 (8544.4－8847.3) | 8709.6 (8559.1－8862.6) |
| 2022 | 8637.2 (8464.1－8813.9) | 8591.3 (8419.1－8767.1) | 8623.7 (8450.8－8800.1) | 8644.1 (8470.8－8820.9) |
| 2023 | 8529.9 (8351.0－8712.6) | 8471.0 (8293.4－8652.5) | 8507.4 (8329.0－8689.6) | 8533.1 (8354.2－8715.9) |
| 2024 | 8301.3 (8122.3－8484.4) | 8249.7 (8071.7－8431.6) | 8279.7 (8101.1－8462.2) | 8305.6 (8126.4－8488.7) |
| 2025 | 8065.1 (7885.6－8248.7) | 8035.0 (7856.2－8217.9) | 8050.1 (7871.0－8233.4) | 8072.2 (7892.6－8255.9) |
| 2026 | 7899.2 (7721.7－8080.7) | 7866.5 (7689.8－8047.3) | 7881.5 (7704.4－8062.6) | 7906.3 (7728.7－8088.0) |
| 2027 | 7781.7 (7586.8－7981.5) | 7738.4 (7544.6－7937.1) | 7759.0 (7564.7－7958.2) | 7789.3 (7594.2－7989.3) |
| 2028 | 7642.2 (7450.4－7838.8) | 7596.8 (7406.2－7792.3) | 7617.4 (7426.3－7813.5) | 7650.5 (7458.5－7847.3) |
| 2029 | 7419.2 (7225.8－7617.8) | 7377.0 (7184.6－7574.4) | 7394.3 (7201.5－7592.3) | 7428.1 (7234.4－7626.9) |
| 2030 | 7177.6 (6990.0－7370.3) | 7143.0 (6956.2－7334.7) | 7154.6 (6967.6－7346.7) | 7187.7 (6999.8－7380.7) |
| 2031 | 6975.4 (6792.8－7162.8) | 6943.5 (6761.8－7130.0) | 6951.9 (6770.0－7138.7) | 6985.5 (6802.7－7173.2) |
| 2032 | 6796.3 (6616.7－6980.7) | 6762.9 (6584.2－6946.5) | 6770.8 (6591.9－6954.6) | 6806.0 (6626.1－6990.7) |
| 2033 | 6624.3 (6449.0－6804.5) | 6590.2 (6415.8－6769.4) | 6597.6 (6423.0－6777.0) | 6634.3 (6458.7－6814.7) |
| 2034 | 6432.8 (6262.4－6607.9) | 6397.0 (6227.5－6571.1) | 6404.5 (6234.9－6578.8) | 6442.8 (6272.1－6618.1) |
| 2035 | 6218.9 (6054.0－6388.3) | 6183.2 (6019.3－6351.7) | 6190.0 (6025.9－6358.6) | 6229.1 (6064.0－6398.8) |
| 2036 | 6002.0 (5842.5－6165.9) | 5969.7 (5811.0－6132.7) | 5974.0 (5815.2－6137.1) | 6013.0 (5853.1－6177.2) |
| 2037 | 5788.2 (5634.3－5946.4) | 5761.3 (5608.0－5918.7) | 5761.8 (5608.6－5919.2) | 5799.9 (5645.6－5958.4) |
| 2038 | 5594.5 (5445.6－5747.6) | 5570.6 (5422.3－5723.0) | 5568.7 (5420.4－5721.1) | 5606.5 (5457.2－5759.9) |
| 2039 | 5422.2 (5276.9－5571.6) | 5394.8 (5250.2－5543.5) | 5394.5 (5249.8－5543.1) | 5433.7 (5288.0－5583.5) |
| 2040 | 5244.6 (5103.7－5389.4) | 5213.1 (5073.0－5357.0) | 5214.6 (5074.5－5358.6) | 5255.4 (5114.2－5400.5) |
| Female, all ages | |  |  |  |
| 2017 | 4003.9 (3955.6－4052.7) | 3941.5 (3894.0－3989.6) | 3961.5 (3913.7－4009.8) | 3970.6 (3922.8－4019.1) |
| 2018 | 4090.4 (4041.3－4140.0) | 3955.8 (3908.3－4003.8) | 4044.7 (3996.1－4093.8) | 4084.6 (4035.6－4134.2) |
| 2019 | 4114.8 (4064.0－4166.1) | 3946.8 (3898.1－3996.0) | 4066.4 (4016.3－4117.2) | 4121.8 (4070.9－4173.2) |
| 2020 | 4069.9 (3982.6－4159.0) | 3860.8 (3778.1－3945.3) | 3994.9 (3909.3－4082.4) | 4059.0 (3972.1－4147.9) |
| 2021 | 4052.6 (3965.1－4142.1) | 3814.1 (3731.7－3898.3) | 3978.7 (3892.7－4066.5) | 4059.1 (3971.4－4148.8) |
| 2022 | 4009.9 (3922.9－4098.9) | 3806.6 (3724.0－3891.1) | 3937.7 (3852.2－4025.1) | 4018.7 (3931.5－4107.8) |
| 2023 | 3980.3 (3892.0－4070.6) | 3774.0 (3690.3－3859.7) | 3876.4 (3790.4－3964.3) | 3959.3 (3871.5－4049.1) |
| 2024 | 4008.2 (3911.0－4107.8) | 3787.3 (3695.4－3881.4) | 3898.0 (3803.5－3994.9) | 3999.9 (3902.9－4099.3) |
| 2025 | 4021.1 (3918.6－4126.2) | 3798.6 (3701.8－3897.8) | 3902.1 (3802.7－4004.1) | 4015.8 (3913.5－4120.8) |
| 2026 | 4004.1 (3898.4－4112.8) | 3761.1 (3661.8－3863.2) | 3857.5 (3755.6－3962.1) | 3981.8 (3876.7－4089.9) |
| 2027 | 3991.8 (3886.3－4100.1) | 3738.6 (3639.9－3840.1) | 3844.2 (3742.7－3948.5) | 3986.8 (3881.5－4095.0) |
| 2028 | 3943.6 (3839.3－4050.6) | 3710.0 (3611.9－3810.7) | 3797.6 (3697.3－3900.7) | 3945.3 (3841.0－4052.4) |
| 2029 | 3885.6 (3778.7－3995.4) | 3654.7 (3554.2－3758.0) | 3719.9 (3617.6－3825.1) | 3869.2 (3762.8－3978.6) |
| 2030 | 3859.1 (3751.9－3969.3) | 3630.6 (3529.9－3734.3) | 3693.1 (3590.6－3798.6) | 3853.9 (3746.9－3963.9) |
| 2031 | 3833.9 (3725.2－3945.7) | 3613.3 (3510.9－3718.7) | 3662.1 (3558.3－3768.9) | 3829.1 (3720.5－3940.8) |
| 2032 | 3805.8 (3697.6－3917.2) | 3571.3 (3469.7－3675.8) | 3610.5 (3507.8－3716.2) | 3784.2 (3676.6－3895.0) |
| 2033 | 3787.4 (3679.3－3898.7) | 3542.3 (3441.2－3646.5) | 3588.0 (3485.6－3693.5) | 3776.5 (3668.7－3887.5) |
| 2034 | 3746.4 (3639.4－3856.6) | 3506.8 (3406.6－3609.9) | 3544.0 (3442.8－3648.2) | 3739.4 (3632.6－3849.3) |
| 2035 | 3689.3 (3581.5－3800.5) | 3446.7 (3345.9－3550.5) | 3471.6 (3370.0－3576.1) | 3670.0 (3562.7－3780.6) |
| 2036 | 3639.7 (3533.3－3749.4) | 3402.3 (3302.8－3504.8) | 3424.4 (3324.3－3527.6) | 3631.2 (3525.0－3740.6) |
| 2037 | 3587.9 (3482.7－3696.4) | 3362.2 (3263.6－3463.8) | 3371.6 (3272.7－3473.5) | 3581.5 (3476.4－3689.7) |
| 2038 | 3538.6 (3434.6－3645.8) | 3309.0 (3211.7－3409.3) | 3307.5 (3210.3－3407.7) | 3520.1 (3416.6－3626.7) |
| 2039 | 3501.1 (3397.7－3607.7) | 3268.8 (3172.2－3368.3) | 3268.3 (3171.7－3367.7) | 3490.1 (3387.0－3596.3) |
| 2040 | 3457.3 (3354.9－3562.9) | 3227.1 (3131.5－3325.6) | 3220.6 (3125.2－3318.9) | 3447.6 (3345.5－3552.8) |
| Total population, 20-49y | |  |  |  |
| 2017 | 1010.4 (1003.6－1017.2) | 1013.7 (1006.9－1020.5) | 1007.4 (1000.7－1014.2) | 1005.1 (998.4－1011.9) |
| 2018 | 1003.1 (996.4－1009.9) | 1014.4 (1007.6－1021.2) | 1001.3 (994.6－1008.0) | 996.6 (990.0－1003.3) |
| 2019 | 1001.3 (980.3－1022.9) | 1014.5 (993.1－1036.3) | 999.6 (978.5－1021.1) | 994.5 (973.6－1015.9) |
| 2020 | 975.8 (947.1－1005.4) | 986.7 (957.7－1016.6) | 972.2 (943.6－1001.6) | 967.5 (939.0－996.8) |
| 2021 | 969.4 (939.6－1000.1) | 977.9 (947.9－1008.9) | 965.0 (935.3－995.5) | 961.1 (931.6－991.6) |
| 2022 | 971.0 (940.8－1002.1) | 977.0 (946.7－1008.3) | 966.3 (936.3－997.2) | 964.6 (934.6－995.5) |
| 2023 | 951.3 (921.8－981.8) | 953.0 (923.4－983.5) | 943.6 (914.3－973.8) | 943.5 (914.2－973.7) |
| 2024 | 946.8 (916.6－977.9) | 948.2 (918.0－979.4) | 938.2 (908.4－969.1) | 939.2 (909.3－970.1) |
| 2025 | 952.5 (921.9－984.1) | 952.3 (921.7－984.0) | 943.2 (912.8－974.5) | 945.6 (915.2－977.0) |
| 2026 | 935.2 (905.1－966.3) | 933.7 (903.7－964.7) | 923.9 (894.2－954.6) | 927.2 (897.4－958.0) |
| 2027 | 931.0 (900.9－962.2) | 931.0 (900.8－962.2) | 919.0 (889.2－949.7) | 922.5 (892.6－953.3) |
| 2028 | 938.0 (907.6－969.4) | 938.2 (907.8－969.6) | 925.8 (895.8－956.8) | 930.4 (900.2－961.5) |
| 2029 | 923.0 (892.9－954.1) | 922.1 (892.0－953.2) | 909.1 (879.4－939.7) | 914.3 (884.5－945.1) |
| 2030 | 918.7 (888.7－949.7) | 919.1 (889.1－950.1) | 904.1 (874.6－934.6) | 909.6 (879.9－940.3) |
| 2031 | 927.9 (897.6－959.2) | 927.7 (897.4－959.0) | 912.7 (883.0－943.5) | 919.4 (889.4－950.4) |
| 2032 | 915.4 (885.4－946.4) | 913.7 (883.7－944.6) | 898.7 (869.2－929.1) | 906.1 (876.4－936.8) |
| 2033 | 911.7 (881.7－942.6) | 910.6 (880.7－941.5) | 894.0 (864.6－924.3) | 901.8 (872.1－932.4) |
| 2034 | 922.6 (892.3－954.0) | 921.0 (890.8－952.3) | 904.4 (874.7－935.1) | 913.4 (883.3－944.4) |
| 2035 | 912.7 (882.6－943.8) | 909.6 (879.6－940.6) | 893.0 (863.5－923.4) | 902.7 (872.9－933.5) |
| 2036 | 908.9 (878.9－940.0) | 906.7 (876.8－937.7) | 888.4 (859.1－918.8) | 898.4 (868.7－929.1) |
| 2037 | 921.5 (891.0－953.0) | 918.9 (888.5－950.3) | 900.3 (870.6－931.1) | 911.4 (881.3－942.6) |
| 2038 | 913.7 (883.4－945.0) | 909.8 (879.6－941.0) | 891.3 (861.7－921.8) | 903.1 (873.2－934.1) |
| 2039 | 910.1 (879.9－941.3) | 907.0 (876.8－938.1) | 886.8 (857.3－917.2) | 898.8 (869.0－929.7) |
| 2040 | 923.9 (893.2－955.6) | 920.4 (889.8－952.1) | 899.9 (870.0－930.8) | 913.0 (882.7－944.4) |
| Total population, 50-69y | |  |  |  |
| 2017 | 7244.3 (7218.1－7270.6) | 7223.8 (7197.7－7250.1) | 7231.5 (7205.4－7257.8) | 7234.6 (7208.4－7260.9) |
| 2018 | 7164.0 (7138.1－7190.0) | 7131.0 (7105.2－7156.9) | 7153.7 (7127.8－7179.6) | 7162.6 (7136.7－7188.6) |
| 2019 | 7127.3 (7100.3－7154.5) | 7092.4 (7065.5－7119.4) | 7119.9 (7092.9－7147.0) | 7131.2 (7104.2－7158.4) |
| 2020 | 7070.1 (7041.9－7098.5) | 7002.4 (6974.4－7030.5) | 7047.3 (7019.1－7075.6) | 7065.7 (7037.4－7094.1) |
| 2021 | 7127.6 (7097.0－7158.3) | 7041.1 (7010.9－7071.4) | 7099.7 (7069.2－7130.3) | 7124.0 (7093.4－7154.7) |
| 2022 | 7201.9 (7166.3－7237.7) | 7123.0 (7087.8－7158.4) | 7169.8 (7134.3－7205.4) | 7195.3 (7159.8－7231.1) |
| 2023 | 7087.1 (7050.9－7123.5) | 6985.2 (6949.5－7021.1) | 7035.9 (6999.9－7072.0) | 7068.2 (7032.1－7104.5) |
| 2024 | 7039.9 (6999.7－7080.3) | 6933.7 (6894.1－6973.5) | 6985.2 (6945.3－7025.3) | 7023.2 (6983.0－7063.5) |
| 2025 | 7096.2 (7051.7－7141.0) | 6992.3 (6948.4－7036.4) | 7039.5 (6995.3－7083.9) | 7081.4 (7036.9－7126.1) |
| 2026 | 6977.5 (6932.5－7022.8) | 6859.5 (6815.2－6904.0) | 6909.8 (6865.2－6954.6) | 6957.5 (6912.7－7002.7) |
| 2027 | 6909.4 (6864.7－6954.3) | 6802.5 (6758.6－6846.7) | 6844.5 (6800.3－6889.0) | 6894.0 (6849.5－6938.8) |
| 2028 | 6873.4 (6826.3－6920.9) | 6778.8 (6732.3－6825.7) | 6808.5 (6761.8－6855.5) | 6858.4 (6811.3－6905.7) |
| 2029 | 6603.6 (6557.4－6650.1) | 6504.3 (6458.8－6550.1) | 6529.0 (6483.3－6575.0) | 6580.2 (6534.1－6626.5) |
| 2030 | 6474.8 (6426.3－6523.7) | 6378.3 (6330.5－6426.5) | 6398.4 (6350.5－6446.8) | 6451.7 (6403.3－6500.4) |
| 2031 | 6487.0 (6438.4－6536.1) | 6390.8 (6342.8－6439.0) | 6405.8 (6357.7－6454.1) | 6461.8 (6413.4－6510.6) |
| 2032 | 6357.6 (6309.9－6405.7) | 6257.6 (6210.7－6305.0) | 6268.4 (6221.3－6315.8) | 6326.2 (6278.7－6374.0) |
| 2033 | 6350.3 (6302.4－6398.5) | 6253.8 (6206.6－6301.2) | 6260.9 (6213.7－6308.4) | 6321.7 (6274.1－6369.7) |
| 2034 | 6387.2 (6339.0－6435.7) | 6296.9 (6249.4－6344.8) | 6296.6 (6249.1－6344.4) | 6359.4 (6311.5－6407.7) |
| 2035 | 6269.0 (6221.7－6316.7) | 6172.6 (6126.0－6219.5) | 6171.3 (6124.8－6218.2) | 6236.8 (6189.8－6284.3) |
| 2036 | 6291.9 (6243.5－6340.7) | 6183.2 (6135.6－6231.2) | 6188.8 (6141.2－6236.8) | 6261.3 (6213.1－6309.8) |
| 2037 | 6412.8 (6363.2－6462.7) | 6299.3 (6250.7－6348.4) | 6302.4 (6253.7－6351.4) | 6379.5 (6330.3－6429.2) |
| 2038 | 6391.7 (6337.9－6445.8) | 6267.8 (6215.1－6320.9) | 6270.7 (6218.0－6323.8) | 6351.6 (6298.2－6405.5) |
| 2039 | 6382.7 (6329.1－6436.9) | 6253.9 (6201.3－6307.0) | 6258.0 (6205.4－6311.0) | 6343.4 (6290.0－6397.2) |
| 2040 | 6380.6 (6326.7－6435.1) | 6260.8 (6207.8－6314.2) | 6254.4 (6201.5－6307.8) | 6340.1 (6286.4－6394.1) |
| Total population, ≥70y | |  |  |  |
| 2017 | 13855.6 (13638.0－14076.7) | 13846.9 (13629.4－14067.8) | 13852.2 (13634.7－14073.2) | 13855.0 (13637.4－14076.0) |
| 2018 | 13775.8 (13559.5－13995.6) | 13749.8 (13533.9－13969.2) | 13764.9 (13548.7－13984.5) | 13772.7 (13556.5－13992.5) |
| 2019 | 13673.9 (13443.6－13908.1) | 13645.8 (13415.9－13879.5) | 13666.2 (13436.1－13900.3) | 13677.2 (13446.8－13911.5) |
| 2020 | 13586.9 (13356.4－13821.4) | 13558.2 (13328.1－13792.2) | 13583.7 (13353.2－13818.1) | 13597.8 (13367.1－13832.5) |
| 2021 | 13437.8 (13202.6－13677.1) | 13395.5 (13161.1－13634.1) | 13430.9 (13195.8－13670.1) | 13450.5 (13215.1－13690.1) |
| 2022 | 13392.3 (13151.7－13637.3) | 13352.3 (13112.4－13596.6) | 13384.0 (13143.6－13628.9) | 13407.6 (13166.7－13652.9) |
| 2023 | 13260.5 (13013.4－13512.3) | 13221.4 (12975.0－13472.4) | 13248.7 (13001.8－13500.2) | 13275.6 (13028.3－13527.7) |
| 2024 | 13174.5 (12928.7－13424.9) | 13129.7 (12884.8－13379.3) | 13155.1 (12909.7－13405.1) | 13186.5 (12940.6－13437.2) |
| 2025 | 13051.4 (12806.4－13301.1) | 13004.4 (12760.3－13253.2) | 13027.7 (12783.1－13276.9) | 13063.4 (12818.1－13313.3) |
| 2026 | 12980.6 (12735.7－13230.2) | 12936.5 (12692.5－13185.3) | 12955.3 (12710.9－13204.4) | 12994.0 (12748.9－13243.9) |
| 2027 | 12829.4 (12586.4－13077.1) | 12785.0 (12542.8－13031.8) | 12801.6 (12559.2－13048.8) | 12844.2 (12601.0－13092.2) |
| 2028 | 12749.9 (12505.6－12999.0) | 12707.3 (12463.8－12955.6) | 12721.7 (12477.9－12970.2) | 12768.1 (12523.5－13017.6) |
| 2029 | 12616.8 (12374.4－12864.0) | 12576.7 (12335.0－12823.1) | 12587.8 (12346.0－12834.5) | 12637.5 (12394.6－12885.1) |
| 2030 | 12526.4 (12285.6－12772.0) | 12487.0 (12246.8－12731.8) | 12495.1 (12254.8－12740.1) | 12547.9 (12306.6－12794.0) |
| 2031 | 12384.7 (12146.3－12627.8) | 12343.8 (12106.2－12586.1) | 12350.3 (12112.6－12592.7) | 12406.8 (12168.0－12650.3) |
| 2032 | 12306.3 (12069.3－12548.0) | 12265.7 (12029.5－12506.6) | 12269.0 (12032.7－12510.0) | 12328.6 (12091.1－12570.7) |
| 2033 | 12162.8 (11927.5－12402.8) | 12121.7 (11887.1－12360.8) | 12122.6 (11888.1－12361.8) | 12185.1 (11949.3－12425.5) |
| 2034 | 12071.5 (11837.4－12310.2) | 12030.5 (11797.3－12268.4) | 12029.2 (11796.0－12267.1) | 12094.9 (11860.4－12334.1) |
| 2035 | 11933.4 (11702.0－12169.4) | 11892.9 (11662.3－12128.1) | 11889.5 (11659.0－12124.6) | 11958.1 (11726.2－12194.6) |
| 2036 | 11842.6 (11613.0－12076.8) | 11803.8 (11574.9－12037.3) | 11797.6 (11568.9－12030.9) | 11868.9 (11638.7－12103.6) |
| 2037 | 11696.9 (11470.0－11928.3) | 11658.4 (11432.2－11889.0) | 11650.6 (11424.6－11881.0) | 11724.6 (11497.2－11956.6) |
| 2038 | 11607.5 (11381.9－11837.5) | 11569.6 (11344.8－11798.9) | 11559.4 (11334.8－11788.5) | 11636.3 (11410.1－11866.9) |
| 2039 | 11466.9 (11243.7－11694.5) | 11429.1 (11206.7－11655.9) | 11417.0 (11194.8－11643.6) | 11496.3 (11272.5－11724.4) |
| 2040 | 11372.2 (11150.8－11598.0) | 11334.8 (11114.2－11559.8) | 11320.5 (11100.1－11545.2) | 11402.3 (11180.4－11628.7) |
| Total population, all ages | |  |  |  |
| 2017 | 5232.5 (5164.0－5301.9) | 5185.4 (5117.5－5254.2) | 5218.5 (5150.1－5287.7) | 5232.2 (5163.7－5301.6) |
| 2018 | 5300.0 (5230.7－5370.3) | 5226.7 (5158.3－5296.0) | 5277.5 (5208.5－5347.5) | 5299.4 (5230.1－5369.6) |
| 2019 | 5390.4 (5314.3－5467.6) | 5239.6 (5165.7－5314.7) | 5343.5 (5268.0－5420.0) | 5389.2 (5313.1－5466.3) |
| 2020 | 5442.3 (5363.0－5522.8) | 5227.6 (5151.4－5304.9) | 5374.3 (5296.0－5453.8) | 5440.5 (5361.2－5520.9) |
| 2021 | 5446.1 (5351.2－5542.6) | 5190.8 (5100.4－5282.9) | 5363.7 (5270.2－5458.8) | 5443.8 (5348.9－5540.3) |
| 2022 | 5414.8 (5306.2－5525.5) | 5139.9 (5036.9－5245.0) | 5324.1 (5217.4－5433.0) | 5412.2 (5303.7－5522.9) |
| 2023 | 5376.9 (5265.1－5490.9) | 5123.8 (5017.3－5232.5) | 5280.3 (5170.6－5392.4) | 5374.0 (5262.4－5488.1) |
| 2024 | 5358.4 (5247.0－5472.1) | 5121.1 (5014.6－5229.8) | 5254.4 (5145.2－5365.9) | 5355.3 (5244.0－5469.0) |
| 2025 | 5371.4 (5255.5－5489.9) | 5135.9 (5025.1－5249.2) | 5255.7 (5142.3－5371.6) | 5368.0 (5252.2－5486.4) |
| 2026 | 5411.1 (5288.1－5537.0) | 5163.1 (5045.7－5283.3) | 5279.1 (5159.1－5402.0) | 5407.2 (5284.2－5533.0) |
| 2027 | 5460.3 (5333.2－5590.4) | 5191.9 (5071.1－5315.6) | 5309.5 (5185.9－5436.0) | 5455.8 (5328.9－5585.8) |
| 2028 | 5499.1 (5371.1－5630.2) | 5211.8 (5090.5－5336.0) | 5330.1 (5206.0－5457.2) | 5494.2 (5366.3－5625.2) |
| 2029 | 5515.3 (5385.7－5648.0) | 5217.9 (5095.3－5343.5) | 5331.1 (5205.8－5459.4) | 5509.9 (5380.4－5642.5) |
| 2030 | 5508.7 (5376.3－5644.2) | 5212.1 (5086.9－5340.4) | 5313.1 (5185.5－5443.9) | 5503.0 (5370.8－5638.4) |
| 2031 | 5489.9 (5356.1－5627.0) | 5201.4 (5074.7－5331.3) | 5285.6 (5156.8－5417.6) | 5483.9 (5350.3－5620.9) |
| 2032 | 5473.2 (5339.6－5610.1) | 5193.6 (5066.8－5323.5) | 5260.6 (5132.2－5392.2) | 5467.0 (5333.5－5603.7) |
| 2033 | 5469.1 (5335.4－5606.3) | 5193.3 (5066.3－5323.5) | 5246.6 (5118.3－5378.1) | 5462.6 (5329.1－5599.6) |
| 2034 | 5480.2 (5345.0－5618.8) | 5200.7 (5072.4－5332.3) | 5245.1 (5115.7－5377.8) | 5473.3 (5338.3－5611.8) |
| 2035 | 5500.8 (5363.9－5641.2) | 5211.7 (5082.0－5344.7) | 5251.3 (5120.6－5385.3) | 5493.6 (5356.9－5633.8) |
| 2036 | 5521.5 (5383.8－5662.6) | 5220.8 (5090.6－5354.3) | 5256.9 (5125.8－5391.3) | 5513.8 (5376.4－5654.8) |
| 2037 | 5533.5 (5395.5－5675.0) | 5223.8 (5093.6－5357.4) | 5255.1 (5124.0－5389.5) | 5525.5 (5387.7－5666.8) |
| 2038 | 5533.5 (5395.0－5675.5) | 5219.9 (5089.3－5353.9) | 5243.2 (5112.0－5377.7) | 5525.2 (5386.9－5667.0) |
| 2039 | 5523.8 (5385.0－5666.2) | 5211.2 (5080.2－5345.5) | 5223.6 (5092.3－5358.2) | 5515.2 (5376.6－5657.4) |
| 2040 | 5510.8 (5372.1－5653.2) | 5201.5 (5070.5－5335.9) | 5201.6 (5070.7－5336.0) | 5502.1 (5363.5－5644.2) |

DALY, disability-adjusted life year; y: year

**Supplementary Table 4: Predicted DALY rates (95% prediction intervals) for diabetes and kidney diseases by age groups, sex (male, female), and total population, 2017–2040.**

| Year | Reference | Better scenario | Moderate scenario | Worse scenario |
| --- | --- | --- | --- | --- |
| Male, 20-49y | |  |  |  |
| 2017 | 416.5 (411.8－421.3) | 415.3 (410.5－420.0) | 415.4 (410.7－420.2) | 415.5 (410.8－420.2) |
| 2018 | 420.7 (416.0－425.6) | 419.2 (414.5－424.0) | 419.3 (414.5－424.1) | 419.4 (414.6－424.2) |
| 2019 | 426.5 (421.5－431.5) | 424.3 (419.4－429.3) | 425.1 (420.2－430.1) | 425.6 (420.7－430.6) |
| 2020 | 433.4 (428.3－438.5) | 429.9 (424.9－435.0) | 431.3 (426.3－436.4) | 432.1 (427.0－437.2) |
| 2021 | 440.6 (433.8－447.5) | 435.9 (429.2－442.8) | 438.0 (431.2－444.8) | 439.1 (432.3－445.9) |
| 2022 | 447.7 (440.8－454.7) | 443.5 (436.6－450.5) | 445.1 (438.2－452.1) | 446.5 (439.6－453.6) |
| 2023 | 456.3 (449.2－463.4) | 451.6 (444.6－458.7) | 452.8 (445.8－459.9) | 454.6 (447.5－461.7) |
| 2024 | 465.2 (458.0－472.6) | 460.2 (453.1－467.5) | 461.0 (453.9－468.3) | 463.1 (456.0－470.4) |
| 2025 | 474.2 (466.9－481.7) | 469.5 (462.2－476.8) | 469.9 (462.6－477.2) | 472.3 (465.0－479.7) |
| 2026 | 484.4 (476.9－492.0) | 479.3 (471.9－486.8) | 479.3 (471.8－486.8) | 482.1 (474.6－489.6) |
| 2027 | 495.3 (487.6－503.0) | 489.8 (482.2－497.5) | 489.3 (481.7－497.0) | 492.5 (484.9－500.2) |
| 2028 | 506.1 (498.2－514.0) | 501.0 (493.2－508.8) | 500.0 (492.3－507.9) | 503.6 (495.8－511.5) |
| 2029 | 518.2 (510.2－526.4) | 512.8 (504.9－520.9) | 511.4 (503.5－519.5) | 515.4 (507.4－523.5) |
| 2030 | 531.3 (523.1－539.6) | 525.5 (517.4－533.8) | 523.6 (515.5－531.8) | 527.9 (519.8－536.2) |
| 2031 | 544.5 (536.1－553.1) | 538.9 (530.6－547.4) | 536.5 (528.2－545.0) | 541.3 (532.9－549.8) |
| 2032 | 559.1 (550.4－567.8) | 553.2 (544.7－561.9) | 550.3 (541.8－558.9) | 555.5 (546.9－564.3) |
| 2033 | 574.8 (565.9－583.8) | 568.4 (559.7－577.4) | 564.9 (556.2－573.8) | 570.6 (561.8－579.6) |
| 2034 | 590.8 (581.7－600.1) | 584.6 (575.6－593.8) | 580.5 (571.5－589.6) | 586.7 (577.6－595.9) |
| 2035 | 608.2 (598.8－617.8) | 601.8 (592.5－611.2) | 597.1 (587.8－606.5) | 603.8 (594.4－613.3) |
| 2036 | 627.0 (617.3－636.9) | 620.1 (610.5－629.8) | 614.7 (605.2－624.3) | 621.9 (612.3－631.7) |
| 2037 | 646.4 (636.4－656.5) | 639.5 (629.6－649.5) | 633.4 (623.6－643.4) | 641.2 (631.3－651.3) |
| 2038 | 667.2 (656.9－677.7) | 660.1 (649.9－670.5) | 653.4 (643.3－663.6) | 661.8 (651.5－672.2) |
| 2039 | 689.7 (679.1－700.6) | 682.1 (671.6－692.8) | 674.6 (664.1－685.2) | 683.6 (673.0－694.4) |
| 2040 | 713.2 (702.2－724.4) | 705.5 (694.6－716.6) | 697.1 (686.4－708.1) | 706.9 (695.9－718.0) |
| Male, 50-69y | |  |  |  |
| 2017 | 1730.5 (1699.1－1762.5) | 1730.4 (1699.0－1762.5) | 1730.6 (1699.2－1762.7) | 1730.7 (1699.2－1762.7) |
| 2018 | 1714.7 (1683.6－1746.5) | 1714.5 (1683.3－1746.2) | 1715.0 (1683.8－1746.7) | 1715.1 (1683.9－1746.8) |
| 2019 | 1698.6 (1667.8－1730.1) | 1698.1 (1667.2－1729.6) | 1699.0 (1668.1－1730.5) | 1699.2 (1668.3－1730.6) |
| 2020 | 1683.9 (1653.2－1715.0) | 1682.9 (1652.3－1714.1) | 1684.4 (1653.7－1715.5) | 1684.5 (1653.9－1715.7) |
| 2021 | 1669.4 (1639.1－1700.3) | 1668.0 (1637.7－1698.9) | 1670.0 (1639.7－1701.0) | 1670.3 (1639.9－1701.2) |
| 2022 | 1655.9 (1625.8－1686.5) | 1651.5 (1621.5－1682.1) | 1656.6 (1626.4－1687.2) | 1656.9 (1626.8－1687.6) |
| 2023 | 1642.9 (1613.0－1673.3) | 1635.6 (1605.9－1665.9) | 1643.6 (1613.8－1674.1) | 1644.0 (1614.2－1674.5) |
| 2024 | 1630.6 (1600.9－1660.8) | 1620.5 (1591.0－1650.5) | 1631.4 (1601.8－1661.6) | 1631.9 (1602.2－1662.1) |
| 2025 | 1618.9 (1589.4－1648.8) | 1606.0 (1576.8－1635.7) | 1619.8 (1590.3－1649.8) | 1620.4 (1590.9－1650.4) |
| 2026 | 1607.8 (1578.5－1637.5) | 1592.2 (1563.2－1621.6) | 1608.7 (1579.5－1638.5) | 1609.4 (1580.2－1639.2) |
| 2027 | 1597.3 (1568.2－1626.8) | 1578.9 (1550.2－1608.2) | 1598.3 (1569.2－1627.9) | 1599.1 (1570.0－1628.7) |
| 2028 | 1587.3 (1558.5－1616.7) | 1566.3 (1537.8－1595.3) | 1588.4 (1559.5－1617.8) | 1589.3 (1560.4－1618.7) |
| 2029 | 1577.9 (1549.3－1607.2) | 1554.3 (1526.0－1583.0) | 1579.0 (1550.3－1608.3) | 1580.0 (1551.3－1609.3) |
| 2030 | 1569.1 (1540.6－1598.1) | 1542.8 (1514.7－1571.3) | 1570.2 (1541.7－1599.3) | 1571.3 (1542.8－1600.4) |
| 2031 | 1560.7 (1532.3－1589.6) | 1531.8 (1504.0－1560.2) | 1561.9 (1533.5－1590.8) | 1563.1 (1534.7－1592.1) |
| 2032 | 1552.9 (1524.6－1581.6) | 1521.4 (1493.7－1549.5) | 1554.0 (1525.8－1582.8) | 1555.4 (1527.1－1584.2) |
| 2033 | 1545.4 (1517.4－1574.1) | 1511.4 (1483.9－1539.4) | 1546.6 (1518.5－1575.3) | 1548.1 (1520.0－1576.8) |
| 2034 | 1538.5 (1510.5－1567.0) | 1501.9 (1474.6－1529.7) | 1539.7 (1511.7－1568.2) | 1541.3 (1513.3－1569.9) |
| 2035 | 1532.0 (1504.1－1560.3) | 1492.9 (1465.7－1520.5) | 1533.1 (1505.3－1561.5) | 1534.9 (1507.0－1563.4) |
| 2036 | 1525.8 (1498.1－1554.1) | 1484.2 (1457.3－1511.7) | 1527.0 (1499.2－1555.3) | 1528.9 (1501.1－1557.2) |
| 2037 | 1520.1 (1492.4－1548.2) | 1476.0 (1449.2－1503.3) | 1521.2 (1493.6－1549.4) | 1523.3 (1495.6－1551.5) |
| 2038 | 1514.7 (1487.1－1542.7) | 1468.1 (1441.5－1495.3) | 1515.8 (1488.3－1543.9) | 1518.1 (1490.5－1546.2) |
| 2039 | 1509.6 (1482.2－1537.6) | 1460.6 (1434.1－1487.7) | 1510.7 (1483.3－1538.7) | 1513.2 (1485.6－1541.2) |
| 2040 | 1504.9 (1477.5－1532.7) | 1453.4 (1427.0－1480.4) | 1506.0 (1478.6－1533.9) | 1508.6 (1481.1－1536.5) |
| Male, ≥70y | |  |  |  |
| 2017 | 3521.6 (3457.3－3587.2) | 3522.1 (3457.7－3587.6) | 3522.2 (3457.8－3587.7) | 3522.2 (3457.9－3587.8) |
| 2018 | 3487.7 (3424.0－3552.6) | 3488.0 (3424.3－3553.0) | 3488.3 (3424.6－3553.3) | 3488.5 (3424.8－3553.4) |
| 2019 | 3453.8 (3377.7－3531.6) | 3454.1 (3378.0－3531.9) | 3454.6 (3378.5－3532.4) | 3454.9 (3378.8－3532.7) |
| 2020 | 3420.6 (3340.1－3503.1) | 3420.9 (3340.3－3503.4) | 3421.6 (3341.0－3504.1) | 3422.1 (3341.5－3504.6) |
| 2021 | 3389.0 (3306.9－3473.1) | 3388.5 (3306.5－3472.6) | 3389.5 (3307.5－3473.6) | 3390.1 (3308.1－3474.3) |
| 2022 | 3358.1 (3275.8－3442.5) | 3357.7 (3275.4－3442.0) | 3358.5 (3276.2－3442.9) | 3359.3 (3277.0－3443.7) |
| 2023 | 3328.6 (3246.6－3412.7) | 3328.0 (3246.0－3412.1) | 3328.7 (3246.6－3412.8) | 3329.6 (3247.6－3413.8) |
| 2024 | 3300.1 (3218.5－3383.7) | 3299.5 (3218.0－3383.1) | 3300.1 (3218.5－3383.7) | 3301.2 (3219.6－3384.8) |
| 2025 | 3272.8 (3191.8－3355.8) | 3272.3 (3191.3－3355.3) | 3272.7 (3191.7－3355.7) | 3274.0 (3193.0－3357.0) |
| 2026 | 3246.5 (3166.1－3328.9) | 3246.3 (3165.9－3328.6) | 3246.5 (3166.2－3328.9) | 3248.0 (3167.6－3330.4) |
| 2027 | 3221.5 (3141.8－3303.3) | 3221.5 (3141.7－3303.2) | 3221.6 (3141.9－3303.4) | 3223.2 (3143.4－3305.1) |
| 2028 | 3198.1 (3118.9－3279.2) | 3197.8 (3118.7－3279.0) | 3197.9 (3118.7－3279.1) | 3199.7 (3120.5－3280.9) |
| 2029 | 3175.6 (3096.9－3256.2) | 3175.4 (3096.8－3256.0) | 3175.3 (3096.7－3255.9) | 3177.3 (3098.6－3257.9) |
| 2030 | 3154.5 (3076.4－3234.6) | 3154.1 (3076.0－3234.2) | 3153.9 (3075.8－3234.0) | 3156.0 (3077.9－3236.1) |
| 2031 | 3134.3 (3056.7－3213.9) | 3133.8 (3056.3－3213.4) | 3133.6 (3056.0－3213.1) | 3135.8 (3058.2－3215.4) |
| 2032 | 3115.1 (3038.0－3194.2) | 3114.7 (3037.6－3193.8) | 3114.3 (3037.2－3193.3) | 3116.7 (3039.5－3195.8) |
| 2033 | 3096.8 (3020.1－3175.4) | 3096.5 (3019.8－3175.1) | 3096.0 (3019.4－3174.6) | 3098.6 (3021.9－3177.3) |
| 2034 | 3079.5 (3003.3－3157.7) | 3079.3 (3003.1－3157.5) | 3078.7 (3002.5－3156.9) | 3081.4 (3005.2－3159.7) |
| 2035 | 3063.3 (2987.4－3141.0) | 3063.1 (2987.2－3140.8) | 3062.4 (2986.6－3140.1) | 3065.3 (2989.4－3143.1) |
| 2036 | 3047.9 (2972.4－3125.2) | 3047.7 (2972.3－3125.1) | 3046.9 (2971.5－3124.3) | 3049.9 (2974.4－3127.4) |
| 2037 | 3033.6 (2958.4－3110.6) | 3033.2 (2958.1－3110.2) | 3032.3 (2957.2－3109.3) | 3035.5 (2960.3－3112.6) |
| 2038 | 3019.9 (2945.1－3096.6) | 3019.5 (2944.8－3096.2) | 3018.5 (2943.8－3095.2) | 3021.9 (2947.0－3098.6) |
| 2039 | 3007.0 (2932.5－3083.3) | 3006.6 (2932.1－3082.9) | 3005.5 (2931.1－3081.8) | 3009.0 (2934.5－3085.4) |
| 2040 | 2994.8 (2920.6－3070.8) | 2994.4 (2920.3－3070.4) | 2993.2 (2919.1－3069.2) | 2996.8 (2922.6－3072.9) |
| Male, all ages | |  |  |  |
| 2017 | 1259.0 (1243.1－1275.0) | 1259.8 (1243.9－1275.8) | 1260.5 (1244.6－1276.5) | 1260.7 (1244.8－1276.7) |
| 2018 | 1273.5 (1257.5－1289.8) | 1270.4 (1254.4－1286.5) | 1274.2 (1258.2－1290.4) | 1275.5 (1259.5－1291.8) |
| 2019 | 1289.8 (1273.4－1306.5) | 1283.9 (1267.6－1300.5) | 1289.5 (1273.1－1306.1) | 1291.3 (1274.9－1307.9) |
| 2020 | 1305.6 (1284.6－1327.0) | 1297.0 (1276.1－1318.2) | 1305.7 (1284.7－1327.1) | 1308.5 (1287.4－1329.9) |
| 2021 | 1322.0 (1298.7－1345.7) | 1312.1 (1289.0－1335.6) | 1323.3 (1300.0－1347.0) | 1326.8 (1303.4－1350.6) |
| 2022 | 1340.6 (1315.9－1365.7) | 1324.5 (1300.1－1349.3) | 1342.0 (1317.4－1367.1) | 1346.5 (1321.7－1371.7) |
| 2023 | 1358.8 (1333.3－1384.8) | 1338.6 (1313.5－1364.2) | 1362.2 (1336.6－1388.2) | 1367.5 (1341.8－1393.6) |
| 2024 | 1379.4 (1353.3－1406.1) | 1353.5 (1327.9－1379.7) | 1383.6 (1357.3－1410.3) | 1389.9 (1363.5－1416.7) |
| 2025 | 1402.8 (1376.1－1430.1) | 1370.0 (1343.9－1396.6) | 1406.5 (1379.7－1433.8) | 1413.7 (1386.8－1441.2) |
| 2026 | 1427.0 (1399.7－1454.8) | 1387.5 (1360.9－1414.5) | 1430.8 (1403.5－1458.7) | 1439.2 (1411.7－1467.2) |
| 2027 | 1452.6 (1424.8－1480.9) | 1406.4 (1379.5－1433.9) | 1456.7 (1428.8－1485.1) | 1466.2 (1438.1－1494.8) |
| 2028 | 1480.6 (1452.2－1509.5) | 1426.7 (1399.4－1454.5) | 1484.2 (1455.8－1513.2) | 1494.9 (1466.2－1524.1) |
| 2029 | 1508.7 (1479.8－1538.1) | 1448.5 (1420.7－1476.8) | 1513.4 (1484.5－1543.0) | 1525.3 (1496.1－1555.1) |
| 2030 | 1538.4 (1508.9－1568.5) | 1471.7 (1443.5－1500.4) | 1544.5 (1514.9－1574.6) | 1557.6 (1527.8－1588.1) |
| 2031 | 1571.0 (1540.9－1601.7) | 1496.5 (1467.8－1525.7) | 1577.3 (1547.1－1608.2) | 1591.9 (1561.4－1623.0) |
| 2032 | 1605.1 (1574.3－1636.4) | 1522.9 (1493.7－1552.7) | 1612.2 (1581.3－1643.7) | 1628.2 (1597.0－1660.0) |
| 2033 | 1641.3 (1609.9－1673.4) | 1551.1 (1521.3－1581.4) | 1649.2 (1617.6－1681.4) | 1666.7 (1634.8－1699.3) |
| 2034 | 1681.0 (1648.8－1713.8) | 1581.0 (1550.7－1611.9) | 1688.3 (1656.0－1721.3) | 1707.5 (1674.8－1740.9) |
| 2035 | 1722.1 (1689.0－1755.7) | 1612.8 (1581.9－1644.4) | 1729.8 (1696.6－1763.6) | 1750.7 (1717.1－1784.9) |
| 2036 | 1765.1 (1731.3－1799.6) | 1646.7 (1615.1－1678.9) | 1773.7 (1739.7－1808.4) | 1796.5 (1762.0－1831.6) |
| 2037 | 1811.4 (1776.7－1846.8) | 1682.6 (1650.4－1715.5) | 1820.3 (1785.4－1855.9) | 1845.0 (1809.6－1881.0) |
| 2038 | 1859.7 (1824.1－1896.1) | 1720.8 (1687.8－1754.4) | 1869.6 (1833.8－1906.2) | 1896.4 (1860.0－1933.4) |
| 2039 | 1910.7 (1874.1－1948.1) | 1761.3 (1727.5－1795.7) | 1921.9 (1885.0－1959.4) | 1950.8 (1913.4－1988.9) |
| 2040 | 1965.9 (1928.3－2004.4) | 1804.2 (1769.6－1839.5) | 1977.2 (1939.3－2015.9) | 2008.5 (1970.0－2047.8) |
| Female, 20-49y | |  |  |  |
| 2017 | 304.0 (302.6－305.4) | 300.6 (299.2－301.9) | 302.9 (301.6－304.3) | 303.7 (302.4－305.1) |
| 2018 | 305.8 (304.4－307.1) | 298.0 (296.7－299.3) | 303.3 (302.0－304.7) | 305.1 (303.8－306.5) |
| 2019 | 306.8 (304.6－309.0) | 292.7 (290.7－294.8) | 302.3 (300.2－304.5) | 305.7 (303.5－307.8) |
| 2020 | 305.1 (302.9－307.3) | 283.4 (281.4－285.5) | 298.2 (296.0－300.3) | 303.4 (301.2－305.6) |
| 2021 | 305.9 (303.5－308.3) | 277.6 (275.4－279.8) | 296.8 (294.5－299.2) | 303.6 (301.3－306.0) |
| 2022 | 306.8 (304.3－309.4) | 270.5 (268.3－272.8) | 295.6 (293.1－298.1) | 304.0 (301.5－306.6) |
| 2023 | 305.7 (303.1－308.3) | 261.6 (259.4－263.9) | 292.2 (289.7－294.8) | 302.3 (299.7－304.9) |
| 2024 | 307.2 (304.6－309.8) | 256.9 (254.7－259.1) | 292.1 (289.6－294.6) | 303.4 (300.8－306.0) |
| 2025 | 309.1 (306.5－311.8) | 252.7 (250.6－254.9) | 292.4 (289.9－294.9) | 304.9 (302.3－307.5) |
| 2026 | 308.9 (306.1－311.8) | 246.6 (244.4－248.9) | 290.5 (287.8－293.2) | 304.3 (301.5－307.1) |
| 2027 | 311.1 (308.3－314.0) | 243.6 (241.4－245.8) | 291.3 (288.6－294.0) | 306.1 (303.3－309.0) |
| 2028 | 313.6 (310.7－316.5) | 240.6 (238.4－242.9) | 292.3 (289.6－295.0) | 308.3 (305.4－311.1) |
| 2029 | 313.8 (310.8－316.8) | 235.2 (233.0－237.5) | 290.8 (288.0－293.5) | 308.0 (305.1－311.0) |
| 2030 | 316.0 (313.0－319.1) | 232.1 (229.9－234.4) | 291.5 (288.7－294.3) | 309.9 (307.0－312.9) |
| 2031 | 318.6 (315.5－321.7) | 229.0 (226.8－231.3) | 292.3 (289.5－295.2) | 312.0 (309.0－315.1) |
| 2032 | 318.8 (315.7－321.9) | 223.5 (221.3－225.7) | 290.6 (287.8－293.5) | 311.7 (308.7－314.8) |
| 2033 | 321.0 (317.8－324.2) | 220.1 (218.0－222.3) | 291.1 (288.2－293.9) | 313.5 (310.4－316.6) |
| 2034 | 323.6 (320.4－326.9) | 216.9 (214.8－219.1) | 291.8 (288.9－294.7) | 315.7 (312.5－318.8) |
| 2035 | 324.0 (320.7－327.2) | 211.7 (209.5－213.8) | 290.2 (287.3－293.2) | 315.5 (312.3－318.7) |
| 2036 | 326.3 (323.0－329.6) | 208.4 (206.4－210.6) | 290.6 (287.7－293.6) | 317.4 (314.2－320.6) |
| 2037 | 329.2 (325.8－332.5) | 205.6 (203.5－207.7) | 291.6 (288.6－294.6) | 319.8 (316.5－323.1) |
| 2038 | 329.8 (326.4－333.3) | 200.9 (198.8－203.0) | 290.3 (287.3－293.3) | 319.9 (316.6－323.3) |
| 2039 | 332.3 (328.9－335.8) | 198.1 (196.0－200.1) | 290.9 (287.9－293.9) | 322.0 (318.7－325.3) |
| 2040 | 335.5 (332.0－339.1) | 195.6 (193.6－197.7) | 292.1 (289.0－295.2) | 324.7 (321.3－328.1) |
| Female, 50-69y | |  |  |  |
| 2017 | 1111.6 (1105.4－1117.8) | 1110.7 (1104.5－1116.9) | 1113.3 (1107.1－1119.5) | 1114.2 (1108.0－1120.5) |
| 2018 | 1113.0 (1106.8－1119.3) | 1105.6 (1099.5－1111.8) | 1111.0 (1104.8－1117.2) | 1113.1 (1106.9－1119.3) |
| 2019 | 1128.9 (1119.6－1138.2) | 1113.9 (1104.8－1123.1) | 1123.3 (1114.1－1132.5) | 1126.8 (1117.6－1136.1) |
| 2020 | 1134.3 (1125.0－1143.6) | 1114.7 (1105.6－1123.9) | 1129.1 (1119.9－1138.5) | 1134.6 (1125.3－1144.0) |
| 2021 | 1148.3 (1138.5－1158.2) | 1120.0 (1110.4－1129.6) | 1139.2 (1129.4－1149.0) | 1146.4 (1136.6－1156.3) |
| 2022 | 1151.8 (1141.5－1162.1) | 1116.0 (1106.1－1126.0) | 1141.1 (1131.0－1151.3) | 1149.6 (1139.4－1159.9) |
| 2023 | 1166.5 (1156.0－1177.0) | 1127.3 (1117.2－1137.5) | 1157.9 (1147.5－1168.4) | 1167.5 (1157.0－1178.1) |
| 2024 | 1179.2 (1168.1－1190.4) | 1131.4 (1120.7－1142.1) | 1167.8 (1156.9－1178.9) | 1178.7 (1167.6－1189.8) |
| 2025 | 1191.9 (1180.4－1203.4) | 1136.6 (1125.7－1147.6) | 1178.4 (1167.1－1189.8) | 1190.3 (1178.9－1201.8) |
| 2026 | 1204.0 (1192.5－1215.8) | 1145.5 (1134.4－1156.6) | 1192.1 (1180.7－1203.7) | 1204.8 (1193.2－1216.5) |
| 2027 | 1228.3 (1216.4－1240.3) | 1159.3 (1148.1－1170.7) | 1212.4 (1200.6－1224.3) | 1226.5 (1214.6－1238.5) |
| 2028 | 1247.8 (1235.5－1260.3) | 1169.0 (1157.5－1180.7) | 1229.2 (1217.0－1241.5) | 1245.0 (1232.7－1257.4) |
| 2029 | 1264.2 (1251.1－1277.4) | 1179.5 (1167.3－1191.8) | 1246.5 (1233.6－1259.5) | 1263.8 (1250.7－1277.0) |
| 2030 | 1288.4 (1274.9－1302.0) | 1192.9 (1180.4－1205.5) | 1267.2 (1254.0－1280.6) | 1286.4 (1272.9－1300.0) |
| 2031 | 1319.7 (1305.9－1333.7) | 1213.1 (1200.4－1226.0) | 1296.0 (1282.3－1309.7) | 1317.2 (1303.4－1331.2) |
| 2032 | 1338.4 (1324.3－1352.6) | 1224.0 (1211.1－1237.0) | 1315.1 (1301.2－1329.0) | 1338.5 (1324.4－1352.7) |
| 2033 | 1367.4 (1353.0－1382.0) | 1241.1 (1228.0－1254.4) | 1340.0 (1325.9－1354.3) | 1365.3 (1350.9－1379.8) |
| 2034 | 1402.5 (1387.7－1417.4) | 1265.0 (1251.7－1278.5) | 1372.2 (1357.7－1386.8) | 1399.4 (1384.6－1414.3) |
| 2035 | 1432.9 (1417.4－1448.5) | 1285.8 (1271.9－1299.8) | 1402.2 (1387.1－1417.5) | 1431.8 (1416.3－1447.4) |
| 2036 | 1467.0 (1451.2－1483.0) | 1306.7 (1292.6－1321.0) | 1431.9 (1416.5－1447.6) | 1463.7 (1447.8－1479.7) |
| 2037 | 1504.5 (1488.0－1521.3) | 1332.6 (1318.0－1347.4) | 1466.8 (1450.7－1483.1) | 1500.6 (1484.1－1517.3) |
| 2038 | 1544.5 (1527.4－1561.8) | 1361.9 (1346.8－1377.2) | 1506.6 (1489.9－1523.5) | 1543.1 (1526.0－1560.4) |
| 2039 | 1589.8 (1572.1－1607.7) | 1391.1 (1375.6－1406.7) | 1547.0 (1529.8－1564.4) | 1586.4 (1568.7－1604.2) |
| 2040 | 1630.7 (1612.3－1649.2) | 1417.6 (1401.6－1433.7) | 1584.4 (1566.6－1602.4) | 1626.7 (1608.4－1645.2) |
| Female, ≥70y | |  |  |  |
| 2017 | 2717.6 (2672.2－2763.7) | 2720.0 (2674.6－2766.2) | 2719.2 (2673.8－2765.4) | 2718.8 (2673.4－2764.9) |
| 2018 | 2716.4 (2671.0－2762.5) | 2718.9 (2673.5－2765.1) | 2716.7 (2671.3－2762.8) | 2715.4 (2670.1－2761.5) |
| 2019 | 2709.1 (2621.7－2799.4) | 2712.6 (2625.1－2803.1) | 2708.4 (2621.0－2798.6) | 2706.0 (2618.7－2796.2) |
| 2020 | 2697.8 (2572.2－2829.5) | 2704.6 (2578.7－2836.6) | 2697.9 (2572.4－2829.6) | 2694.3 (2568.9－2825.8) |
| 2021 | 2685.3 (2528.0－2852.4) | 2695.1 (2537.3－2862.9) | 2685.8 (2528.5－2853.0) | 2680.7 (2523.6－2847.5) |
| 2022 | 2671.1 (2489.3－2866.2) | 2682.5 (2499.9－2878.4) | 2673.4 (2491.4－2868.7) | 2666.7 (2485.2－2861.5) |
| 2023 | 2656.4 (2457.0－2872.1) | 2670.6 (2470.1－2887.3) | 2661.5 (2461.7－2877.5) | 2653.2 (2454.0－2868.5) |
| 2024 | 2644.2 (2432.9－2873.9) | 2660.0 (2447.4－2891.0) | 2651.0 (2439.1－2881.2) | 2641.0 (2430.0－2870.4) |
| 2025 | 2633.9 (2415.2－2872.3) | 2651.3 (2431.2－2891.3) | 2642.2 (2422.9－2881.4) | 2630.7 (2412.4－2868.9) |
| 2026 | 2626.0 (2403.3－2869.3) | 2644.7 (2420.4－2889.8) | 2635.8 (2412.2－2880.0) | 2622.7 (2400.3－2865.8) |
| 2027 | 2621.4 (2396.8－2867.2) | 2640.5 (2414.2－2888.1) | 2631.8 (2406.2－2878.5) | 2617.3 (2392.9－2862.6) |
| 2028 | 2619.1 (2393.6－2865.8) | 2638.8 (2411.6－2887.4) | 2630.3 (2403.9－2878.1) | 2614.4 (2389.3－2860.7) |
| 2029 | 2619.1 (2393.3－2866.1) | 2639.5 (2412.0－2888.5) | 2631.5 (2404.7－2879.7) | 2614.2 (2388.8－2860.7) |
| 2030 | 2621.7 (2395.7－2869.0) | 2642.6 (2414.8－2891.9) | 2635.1 (2407.9－2883.6) | 2616.5 (2390.9－2863.3) |
| 2031 | 2626.4 (2400.0－2874.2) | 2648.0 (2419.7－2897.8) | 2641.1 (2413.4－2890.2) | 2621.2 (2395.2－2868.4) |
| 2032 | 2633.4 (2406.2－2881.9) | 2655.5 (2426.5－2906.2) | 2649.3 (2420.8－2899.4) | 2628.1 (2401.4－2876.2) |
| 2033 | 2642.8 (2414.6－2892.5) | 2665.0 (2434.9－2916.8) | 2659.5 (2429.9－2910.8) | 2637.1 (2409.4－2886.3) |
| 2034 | 2654.1 (2424.6－2905.2) | 2676.2 (2444.9－2929.5) | 2671.6 (2440.6－2924.4) | 2648.0 (2419.0－2898.5) |
| 2035 | 2667.2 (2436.3－2920.0) | 2689.2 (2456.4－2944.1) | 2685.4 (2452.9－2939.9) | 2660.5 (2430.2－2912.7) |
| 2036 | 2681.9 (2449.5－2936.4) | 2703.6 (2469.3－2960.2) | 2700.7 (2466.6－2957.0) | 2674.6 (2442.8－2928.4) |
| 2037 | 2697.9 (2463.8－2954.1) | 2719.5 (2483.6－2977.9) | 2717.5 (2481.7－2975.6) | 2690.1 (2456.7－2945.7) |
| 2038 | 2715.0 (2479.3－2973.1) | 2736.7 (2499.1－2996.8) | 2735.5 (2498.1－2995.6) | 2706.9 (2471.9－2964.2) |
| 2039 | 2733.4 (2496.0－2993.4) | 2755.1 (2515.8－3017.1) | 2754.8 (2515.6－3016.8) | 2724.9 (2488.2－2984.0) |
| 2040 | 2752.8 (2513.7－3014.7) | 2774.6 (2533.5－3038.5) | 2775.2 (2534.1－3039.2) | 2743.9 (2505.6－3005.0) |
| Female, all ages | |  |  |  |
| 2017 | 1039.6 (1032.5－1046.8) | 1039.5 (1032.3－1046.6) | 1042.7 (1035.5－1049.9) | 1043.9 (1036.8－1051.1) |
| 2018 | 1075.0 (1067.7－1082.4) | 1065.7 (1058.4－1073.1) | 1074.0 (1066.7－1081.5) | 1077.3 (1069.9－1084.7) |
| 2019 | 1105.4 (1095.4－1115.6) | 1086.7 (1076.8－1096.7) | 1101.4 (1091.3－1111.5) | 1107.1 (1097.0－1117.2) |
| 2020 | 1132.2 (1121.3－1143.1) | 1110.3 (1099.7－1121.0) | 1129.3 (1118.5－1140.2) | 1136.5 (1125.7－1147.5) |
| 2021 | 1164.3 (1153.0－1175.7) | 1137.3 (1126.3－1148.4) | 1159.1 (1147.8－1170.5) | 1167.1 (1155.8－1178.6) |
| 2022 | 1191.7 (1178.8－1204.7) | 1158.3 (1145.8－1171.0) | 1185.9 (1173.1－1198.9) | 1194.4 (1181.5－1207.4) |
| 2023 | 1224.3 (1210.4－1238.4) | 1189.3 (1175.7－1202.9) | 1222.5 (1208.6－1236.6) | 1231.2 (1217.2－1245.4) |
| 2024 | 1267.4 (1252.8－1282.2) | 1221.8 (1207.8－1236.0) | 1263.4 (1248.9－1278.1) | 1273.5 (1258.9－1288.3) |
| 2025 | 1314.5 (1299.3－1329.8) | 1256.9 (1242.3－1271.6) | 1308.9 (1293.7－1324.1) | 1321.0 (1305.8－1336.5) |
| 2026 | 1368.6 (1351.8－1385.5) | 1299.9 (1284.0－1316.1) | 1364.0 (1347.3－1380.9) | 1378.8 (1361.9－1395.9) |
| 2027 | 1425.4 (1407.6－1443.4) | 1339.4 (1322.7－1356.3) | 1416.5 (1398.9－1434.4) | 1434.4 (1416.5－1452.5) |
| 2028 | 1485.5 (1466.9－1504.4) | 1385.1 (1367.8－1402.7) | 1474.2 (1455.7－1492.9) | 1494.3 (1475.6－1513.2) |
| 2029 | 1545.5 (1526.1－1565.1) | 1435.1 (1417.2－1453.4) | 1536.0 (1516.8－1555.6) | 1558.2 (1538.7－1578.0) |
| 2030 | 1610.3 (1589.4－1631.5) | 1485.3 (1466.0－1504.9) | 1598.1 (1577.3－1619.1) | 1622.2 (1601.1－1643.6) |
| 2031 | 1686.9 (1664.9－1709.3) | 1547.9 (1527.7－1568.4) | 1673.7 (1651.8－1695.8) | 1699.8 (1677.6－1722.3) |
| 2032 | 1765.8 (1742.7－1789.2) | 1612.1 (1591.0－1633.5) | 1754.2 (1731.2－1777.4) | 1783.6 (1760.2－1807.2) |
| 2033 | 1858.9 (1834.5－1883.6) | 1682.8 (1660.7－1705.1) | 1843.2 (1819.0－1867.6) | 1876.4 (1851.8－1901.3) |
| 2034 | 1964.4 (1938.1－1991.1) | 1763.7 (1740.1－1787.7) | 1945.2 (1919.1－1971.7) | 1983.0 (1956.5－2010.0) |
| 2035 | 2066.9 (2039.2－2095.0) | 1843.6 (1818.8－1868.6) | 2047.7 (2020.2－2075.6) | 2090.6 (2062.5－2119.0) |
| 2036 | 2187.0 (2157.7－2216.8) | 1935.6 (1909.6－1962.0) | 2162.8 (2133.7－2192.2) | 2210.3 (2180.6－2240.4) |
| 2037 | 2313.9 (2282.8－2345.4) | 2034.2 (2006.9－2062.0) | 2286.2 (2255.5－2317.4) | 2338.8 (2307.4－2370.7) |
| 2038 | 2443.1 (2409.7－2476.9) | 2137.9 (2108.7－2167.5) | 2416.2 (2383.2－2449.6) | 2473.9 (2440.2－2508.2) |
| 2039 | 2599.8 (2564.3－2635.8) | 2259.8 (2228.9－2291.1) | 2567.8 (2532.8－2603.4) | 2631.4 (2595.5－2667.9) |
| 2040 | 2765.0 (2727.3－2803.3) | 2386.0 (2353.4－2419.0) | 2728.6 (2691.3－2766.4) | 2799.6 (2761.4－2838.4) |
| Total population, 20-49y | |  |  |  |
| 2017 | 367.7 (367.4－367.9) | 367.4 (367.1－367.6) | 367.1 (366.9－367.4) | 367.1 (366.8－367.3) |
| 2018 | 382.8 (382.6－383.1) | 382.9 (382.6－383.1) | 382.3 (382.1－382.6) | 382.2 (381.9－382.4) |
| 2019 | 397.9 (397.6－398.2) | 398.0 (397.7－398.2) | 397.4 (397.1－397.7) | 397.2 (397.0－397.5) |
| 2020 | 414.1 (413.8－414.4) | 413.4 (413.1－413.7) | 413.1 (412.9－413.4) | 413.2 (412.9－413.5) |
| 2021 | 434.7 (434.4－435.1) | 433.2 (432.8－433.6) | 433.5 (433.2－433.9) | 433.9 (433.5－434.2) |
| 2022 | 451.6 (451.2－452.1) | 449.9 (449.5－450.4) | 450.4 (450.0－450.8) | 451.0 (450.6－451.5) |
| 2023 | 466.9 (466.4－467.4) | 464.4 (463.9－464.9) | 465.0 (464.5－465.5) | 465.9 (465.4－466.4) |
| 2024 | 483.1 (482.6－483.6) | 480.3 (479.8－480.8) | 480.9 (480.4－481.4) | 482.2 (481.7－482.7) |
| 2025 | 491.1 (490.6－491.6) | 488.7 (488.2－489.2) | 488.8 (488.3－489.3) | 490.2 (489.7－490.7) |
| 2026 | 506.4 (505.9－506.9) | 504.0 (503.5－504.6) | 503.6 (503.1－504.1) | 505.0 (504.5－505.5) |
| 2027 | 525.7 (525.1－526.3) | 523.7 (523.1－524.3) | 522.5 (521.9－523.1) | 524.0 (523.4－524.7) |
| 2028 | 544.3 (543.6－544.9) | 542.7 (542.1－543.3) | 541.1 (540.4－541.7) | 542.8 (542.1－543.4) |
| 2029 | 567.1 (566.5－567.8) | 565.4 (564.7－566.0) | 563.3 (562.6－563.9) | 565.1 (564.5－565.8) |
| 2030 | 599.5 (598.8－600.2) | 598.1 (597.4－598.8) | 595.3 (594.6－596.0) | 597.3 (596.6－598.0) |
| 2031 | 631.7 (630.9－632.4) | 631.4 (630.6－632.1) | 627.2 (626.5－628.0) | 629.3 (628.5－630.0) |
| 2032 | 674.9 (674.0－675.7) | 675.0 (674.2－675.8) | 669.7 (668.9－670.5) | 671.8 (671.0－672.6) |
| 2033 | 722.3 (721.4－723.3) | 722.7 (721.8－723.6) | 716.4 (715.5－717.3) | 718.8 (717.9－719.7) |
| 2034 | 762.8 (761.8－763.7) | 763.3 (762.3－764.3) | 756.4 (755.5－757.4) | 759.3 (758.3－760.2) |
| 2035 | 814.7 (813.6－815.8) | 814.3 (813.2－815.4) | 807.3 (806.2－808.3) | 810.7 (809.7－811.8) |
| 2036 | 864.5 (863.3－865.6) | 863.1 (862.0－864.3) | 856.1 (854.9－857.2) | 860.3 (859.2－861.5) |
| 2037 | 906.2 (905.0－907.5) | 904.1 (902.9－905.4) | 897.2 (896.0－898.4) | 902.2 (901.0－903.5) |
| 2038 | 948.8 (947.5－950.1) | 946.2 (944.8－947.5) | 938.8 (937.5－940.1) | 944.4 (943.1－945.7) |
| 2039 | 998.6 (997.2－1000.0) | 996.5 (995.1－997.9) | 987.6 (986.2－989.0) | 993.5 (992.1－994.9) |
| 2040 | 1047.1 (1045.6－1048.6) | 1047.2 (1045.7－1048.7) | 1035.7 (1034.2－1037.1) | 1041.5 (1040.0－1043.0) |
| Total population, 50-69y | |  |  |  |
| 2017 | 1424.0 (1410.7－1437.4) | 1426.8 (1413.5－1440.2) | 1426.5 (1413.2－1439.9) | 1426.4 (1413.1－1439.7) |
| 2018 | 1413.7 (1400.6－1427.0) | 1410.4 (1397.3－1423.7) | 1413.3 (1400.2－1426.6) | 1414.2 (1401.1－1427.5) |
| 2019 | 1427.1 (1413.7－1440.5) | 1422.3 (1409.0－1435.6) | 1425.4 (1412.1－1438.9) | 1426.3 (1413.0－1439.8) |
| 2020 | 1427.3 (1407.4－1447.4) | 1423.6 (1403.8－1443.7) | 1427.7 (1407.8－1447.8) | 1428.7 (1408.8－1448.9) |
| 2021 | 1453.3 (1428.7－1478.2) | 1453.3 (1428.7－1478.2) | 1454.7 (1430.1－1479.7) | 1454.5 (1430.0－1479.5) |
| 2022 | 1467.8 (1442.9－1493.1) | 1468.7 (1443.8－1494.0) | 1471.0 (1446.0－1496.3) | 1469.7 (1444.8－1495.1) |
| 2023 | 1496.3 (1465.7－1527.6) | 1503.7 (1472.9－1535.1) | 1504.7 (1473.9－1536.1) | 1501.5 (1470.8－1532.9) |
| 2024 | 1515.2 (1484.2－1546.9) | 1523.1 (1491.9－1554.9) | 1524.5 (1493.2－1556.4) | 1520.0 (1488.8－1551.8) |
| 2025 | 1545.3 (1512.0－1579.3) | 1554.6 (1521.1－1588.8) | 1555.7 (1522.2－1590.0) | 1549.6 (1516.2－1583.7) |
| 2026 | 1560.9 (1526.4－1596.2) | 1570.6 (1535.9－1606.1) | 1573.7 (1538.9－1609.2) | 1566.7 (1532.1－1602.1) |
| 2027 | 1589.7 (1554.4－1625.7) | 1597.6 (1562.2－1633.8) | 1602.3 (1566.8－1638.6) | 1594.4 (1559.0－1630.5) |
| 2028 | 1609.0 (1572.0－1647.0) | 1614.3 (1577.1－1652.4) | 1622.1 (1584.7－1660.3) | 1613.7 (1576.6－1651.8) |
| 2029 | 1637.3 (1599.6－1675.9) | 1643.9 (1606.0－1682.7) | 1654.0 (1615.8－1693.0) | 1644.8 (1606.9－1683.6) |
| 2030 | 1663.7 (1624.7－1703.5) | 1668.2 (1629.2－1708.2) | 1681.2 (1641.8－1721.5) | 1671.4 (1632.2－1711.4) |
| 2031 | 1700.9 (1660.8－1742.0) | 1705.8 (1665.6－1746.9) | 1720.4 (1679.9－1761.9) | 1709.4 (1669.2－1750.7) |
| 2032 | 1733.4 (1692.4－1775.3) | 1739.6 (1698.5－1781.7) | 1756.4 (1714.9－1798.8) | 1744.3 (1703.1－1786.5) |
| 2033 | 1778.6 (1736.2－1822.1) | 1784.4 (1741.8－1828.0) | 1802.4 (1759.4－1846.5) | 1788.9 (1746.2－1832.6) |
| 2034 | 1819.9 (1776.5－1864.5) | 1825.1 (1781.6－1869.8) | 1845.0 (1801.0－1890.1) | 1830.1 (1786.4－1874.8) |
| 2035 | 1867.0 (1822.1－1912.9) | 1874.3 (1829.3－1920.4) | 1895.7 (1850.2－1942.3) | 1879.2 (1834.0－1925.4) |
| 2036 | 1913.4 (1867.4－1960.6) | 1919.5 (1873.4－1966.8) | 1943.3 (1896.5－1991.1) | 1925.4 (1879.1－1972.8) |
| 2037 | 1965.9 (1918.6－2014.4) | 1972.4 (1924.9－2021.1) | 1998.4 (1950.3－2047.7) | 1979.0 (1931.3－2027.8) |
| 2038 | 2015.1 (1966.5－2065.0) | 2023.1 (1974.2－2073.1) | 2052.0 (2002.5－2102.8) | 2031.3 (1982.2－2081.5) |
| 2039 | 2075.0 (2024.9－2126.4) | 2082.0 (2031.7－2133.6) | 2113.8 (2062.8－2166.1) | 2091.5 (2040.9－2143.2) |
| 2040 | 2134.8 (2083.1－2187.7) | 2140.9 (2089.1－2194.0) | 2176.0 (2123.3－2229.9) | 2152.1 (2100.0－2205.5) |
| Total population, ≥70y | |  |  |  |
| 2017 | 3048.1 (3008.1－3088.7) | 3048.0 (3008.0－3088.6) | 3048.1 (3008.0－3088.6) | 3048.1 (3008.1－3088.6) |
| 2018 | 3050.3 (3010.3－3090.8) | 3048.0 (3008.0－3088.5) | 3049.2 (3009.2－3089.8) | 3049.9 (3009.9－3090.5) |
| 2019 | 3040.7 (3000.7－3081.2) | 3037.5 (2997.6－3077.9) | 3039.9 (3000.0－3080.4) | 3041.3 (3001.3－3081.8) |
| 2020 | 3044.3 (2992.0－3097.5) | 3040.5 (2988.3－3093.7) | 3044.1 (2991.8－3097.3) | 3046.1 (2993.8－3099.3) |
| 2021 | 3038.5 (2986.3－3091.7) | 3033.4 (2981.2－3086.4) | 3038.0 (2985.8－3091.2) | 3040.7 (2988.5－3093.9) |
| 2022 | 3046.0 (2993.6－3099.3) | 3041.2 (2988.9－3094.5) | 3045.3 (2992.9－3098.6) | 3048.6 (2996.2－3102.0) |
| 2023 | 3043.8 (2991.4－3097.1) | 3038.9 (2986.6－3092.1) | 3042.4 (2990.1－3095.7) | 3046.4 (2994.0－3099.8) |
| 2024 | 3055.2 (3002.6－3108.8) | 3049.9 (2997.4－3103.3) | 3052.8 (3000.2－3106.3) | 3057.4 (3004.8－3111.0) |
| 2025 | 3056.2 (3003.5－3109.8) | 3050.8 (2998.2－3104.3) | 3053.1 (3000.5－3106.7) | 3058.4 (3005.7－3112.0) |
| 2026 | 3070.1 (3017.2－3124.0) | 3064.9 (3012.1－3118.7) | 3066.7 (3013.8－3120.5) | 3072.6 (3019.6－3126.5) |
| 2027 | 3074.1 (3021.0－3128.1) | 3069.0 (3016.1－3122.9) | 3070.2 (3017.3－3124.2) | 3076.8 (3023.7－3130.9) |
| 2028 | 3090.9 (3037.5－3145.2) | 3086.5 (3033.2－3140.7) | 3087.1 (3033.8－3141.3) | 3094.3 (3040.9－3148.7) |
| 2029 | 3098.0 (3044.5－3152.5) | 3093.9 (3040.4－3148.3) | 3094.0 (3040.5－3148.4) | 3101.9 (3048.3－3156.4) |
| 2030 | 3118.7 (3064.7－3173.5) | 3114.7 (3060.8－3169.5) | 3114.2 (3060.3－3169.0) | 3122.8 (3068.8－3177.7) |
| 2031 | 3129.5 (3075.3－3184.6) | 3125.5 (3071.4－3180.6) | 3124.5 (3070.4－3179.5) | 3133.8 (3079.5－3188.9) |
| 2032 | 3153.8 (3099.2－3209.4) | 3149.9 (3095.4－3205.4) | 3148.2 (3093.7－3203.7) | 3158.2 (3103.5－3213.8) |
| 2033 | 3168.3 (3113.4－3224.2) | 3164.3 (3109.5－3220.1) | 3162.1 (3107.3－3217.9) | 3172.8 (3117.8－3228.7) |
| 2034 | 3196.2 (3140.8－3252.6) | 3192.4 (3137.0－3248.7) | 3189.6 (3134.3－3245.8) | 3201.0 (3145.5－3257.4) |
| 2035 | 3214.2 (3158.4－3270.9) | 3210.5 (3154.8－3267.2) | 3207.2 (3151.6－3263.8) | 3219.3 (3163.4－3276.1) |
| 2036 | 3245.9 (3189.6－3303.3) | 3242.6 (3186.3－3299.8) | 3238.6 (3182.4－3295.8) | 3251.4 (3195.0－3308.9) |
| 2037 | 3267.8 (3211.1－3325.6) | 3264.7 (3208.0－3322.4) | 3260.2 (3203.5－3317.8) | 3273.7 (3216.9－3331.6) |
| 2038 | 3303.8 (3246.4－3362.3) | 3300.9 (3243.6－3359.3) | 3295.7 (3238.5－3354.0) | 3310.1 (3252.6－3368.7) |
| 2039 | 3330.1 (3272.2－3389.1) | 3327.3 (3269.4－3386.1) | 3321.5 (3263.7－3380.3) | 3336.7 (3278.6－3395.7) |
| 2040 | 3370.7 (3312.1－3430.4) | 3368.0 (3309.4－3427.7) | 3361.6 (3303.1－3421.1) | 3377.6 (3318.8－3437.4) |
| Total population, all ages | |  |  |  |
| 2017 | 1127.2 (1115.8－1138.7) | 1125.7 (1114.3－1137.2) | 1126.7 (1115.4－1138.3) | 1127.2 (1115.8－1138.7) |
| 2018 | 1163.3 (1151.6－1175.0) | 1163.6 (1151.9－1175.4) | 1163.3 (1151.7－1175.1) | 1163.3 (1151.6－1175.0) |
| 2019 | 1194.1 (1182.0－1206.3) | 1195.1 (1183.0－1207.3) | 1194.3 (1182.2－1206.6) | 1194.1 (1182.0－1206.3) |
| 2020 | 1221.1 (1208.5－1233.7) | 1221.0 (1208.4－1233.7) | 1220.9 (1208.4－1233.6) | 1221.0 (1208.5－1233.7) |
| 2021 | 1242.7 (1223.5－1262.2) | 1241.1 (1222.0－1260.6) | 1242.1 (1222.9－1261.5) | 1242.7 (1223.5－1262.2) |
| 2022 | 1264.9 (1245.1－1285.0) | 1261.5 (1241.7－1281.5) | 1263.6 (1243.8－1283.7) | 1264.9 (1245.1－1285.0) |
| 2023 | 1289.5 (1269.1－1310.2) | 1285.9 (1265.6－1306.6) | 1287.7 (1267.4－1308.4) | 1289.5 (1269.1－1310.2) |
| 2024 | 1318.2 (1296.9－1339.7) | 1314.6 (1293.4－1336.1) | 1315.9 (1294.7－1337.4) | 1318.1 (1296.9－1339.7) |
| 2025 | 1349.4 (1327.6－1371.5) | 1346.1 (1324.3－1368.2) | 1346.7 (1325.0－1368.8) | 1349.3 (1327.5－1371.4) |
| 2026 | 1382.5 (1360.1－1405.3) | 1379.4 (1357.0－1402.1) | 1379.4 (1357.0－1402.1) | 1382.4 (1360.0－1405.2) |
| 2027 | 1416.8 (1393.8－1440.1) | 1413.8 (1390.8－1437.0) | 1413.2 (1390.3－1436.5) | 1416.7 (1393.7－1440.0) |
| 2028 | 1452.6 (1429.1－1476.6) | 1449.6 (1426.1－1473.5) | 1448.4 (1425.0－1472.3) | 1452.5 (1428.9－1476.4) |
| 2029 | 1490.3 (1466.1－1514.9) | 1487.3 (1463.1－1511.8) | 1485.5 (1461.4－1510.0) | 1490.1 (1466.0－1514.7) |
| 2030 | 1530.3 (1505.5－1555.6) | 1527.3 (1502.5－1552.5) | 1525.0 (1500.2－1550.1) | 1530.2 (1505.3－1555.4) |
| 2031 | 1572.8 (1547.3－1598.7) | 1569.9 (1544.4－1595.8) | 1566.9 (1541.4－1592.7) | 1572.6 (1547.1－1598.6) |
| 2032 | 1617.9 (1591.6－1644.5) | 1615.0 (1588.8－1641.6) | 1611.3 (1585.1－1637.8) | 1617.6 (1591.4－1644.3) |
| 2033 | 1665.5 (1638.4－1692.9) | 1662.7 (1635.7－1690.1) | 1658.2 (1631.3－1685.5) | 1665.2 (1638.2－1692.7) |
| 2034 | 1715.7 (1687.9－1744.0) | 1713.0 (1685.2－1741.3) | 1707.8 (1680.0－1735.9) | 1715.5 (1687.6－1743.8) |
| 2035 | 1768.8 (1740.1－1798.0) | 1766.2 (1737.5－1795.3) | 1760.1 (1731.5－1789.1) | 1768.5 (1739.8－1797.7) |
| 2036 | 1824.9 (1795.3－1855.0) | 1822.3 (1792.8－1852.4) | 1815.4 (1785.9－1845.3) | 1824.6 (1795.0－1854.7) |
| 2037 | 1884.2 (1853.6－1915.3) | 1881.7 (1851.2－1912.8) | 1873.9 (1843.5－1904.8) | 1883.9 (1853.3－1915.0) |
| 2038 | 1946.9 (1915.4－1979.1) | 1944.6 (1913.0－1976.6) | 1935.7 (1904.3－1967.7) | 1946.6 (1915.0－1978.7) |
| 2039 | 2013.3 (1980.6－2046.5) | 2011.0 (1978.4－2044.2) | 2001.2 (1968.7－2034.2) | 2012.9 (1980.3－2046.1) |
| 2040 | 2083.5 (2049.7－2117.8) | 2081.3 (2047.5－2115.6) | 2070.4 (2036.8－2104.5) | 2083.1 (2049.3－2117.4) |

DALY, disability-adjusted life year; y: year
